# Peptide Amphiphiles as Biodegradable Adjuvants for Efficient Retroviral Gene Delivery

**DOI:** 10.1002/adhm.202301364

**Published:** 2023-11-23

**Authors:** Kübra Kaygisiz, Lena Rauch‐Wirth, Aysenur Iscen, Jan Hartenfels, Kurt Kremer, Jan Münch, Christopher V. Synatschke, Tanja Weil

**Affiliations:** ^1^ Department Synthesis of Macromolecules Max Planck Institute for Polymer Research Ackermannweg 10 55128 Mainz Germany; ^2^ Institute of Molecular Virology Ulm University Medical Center Meyerhofstraße 1 89081 Ulm Germany; ^3^ Polymer Theory Department Max Planck Institute for Polymer Research Ackermannweg 10 55128 Mainz Germany

**Keywords:** biodegradability, gene delivery, nanofibrils, peptide amphiphiles, retroviral transduction enhancer, self‐assembly

## Abstract

Retroviral gene delivery is the key technique for in vitro and ex vivo gene therapy. However, inefficient virion‐cell attachment resulting in low gene transduction efficacy remains a major challenge in clinical applications. Adjuvants for ex vivo therapy settings need to increase transduction efficiency while being easily removed or degraded post‐transduction to prevent the risk of venous embolism after infusing the transduced cells back to the bloodstream of patients, yet no such peptide system have been reported thus far. In this study, peptide amphiphiles (PAs) with a hydrophobic fatty acid and a hydrophilic peptide moiety that reveal enhanced viral transduction efficiency are introduced. The PAs form β‐sheet‐rich fibrils that assemble into positively charged aggregates, promoting virus adhesion to the cell membrane. The block‐type amphiphilic sequence arrangement in the PAs ensures efficient cell‐virus interaction and biodegradability. Good biodegradability is observed for fibrils forming small aggregates and it is shown that via molecular dynamics simulations, the fibril‐fibril interactions of PAs are governed by fibril surface hydrophobicity. These findings establish PAs as additives in retroviral gene transfer, rivalling commercially available transduction enhancers in efficiency and degradability with promising translational options in clinical gene therapy applications.

## Graphical abstract

Peptide amphiphiles as enhancers of retroviral gene delivery through improved cell‐virus interaction and biodegradability are reported. The peptide amphiphiles exhibit comparable efficacy to existing transduction enhancers and hold promise for potential use in ex vivo gene therapy.

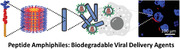

## 1 Introduction

Non‐pathogenic viral particles offer promising perspectives as vectors for the delivery of genetic material into cells in the context of gene therapy^[^ ^1^, ^2^ ^]^ and vaccines.^[^ ^3^, ^4^ ^]^ Lentiviruses and γ‐retroviruses are the vectors of choice in fundamental research as well as most clinical trials that are currently underway.^[^ ^5^, ^6^, ^7^ ^]^ These vectors are superior compared to other viral vectors as they do not replicate and induce only very low inflammatory responses, which is essential for safe, stable and long‐lasting gene expression.^[^ ^8^, ^9^, ^10^ ^]^ For instance, chimeric antigen receptor (CAR) T cell therapy is one of the most promising applications of gene therapy approved for patient use.^[^ ^11^ ^]^ For the generation of CAR T cells, the T cells of the patient are isolated, activated and genetically modified ex vivo to express the CAR. After infusion of CAR T cells back into the patient, the CAR T cells recognize specific tumor surface antigens by their CAR receptor, which mediates the elimination of tumor cells.^[^ ^12^ ^]^ An indispensable but challenging step in the engineering process of CAR T cells is the efficient introduction of the CAR gene via retroviral transduction.^[^ ^13^ ^]^ However, low concentrations of the viral vectors must be used to avoid side effects such as cytotoxicity and immunogenic reactions. Therefore, efficient virion‐cell attachment is essential for gene transduction, which remains a major challenge in retroviral gene delivery.^[^ ^14^, ^15^, ^16^, ^17^ ^]^ In ex vivo clinical applications, it is of utmost importance to prevent exposure of possibly harmful substances to the patient.^[^ ^18^ ^]^ Therefore, any aggregated adjuvants should be removed or degraded after successful transduction.

To overcome these challenges, transduction‐enhancing additives, including polymers, lipids, peptides and others, have been reported.^[^ ^19^ ^]^ Self‐assembling peptides which form nanofibrils with a positive net charge and a high β‐sheet secondary structure, so called amyloids, have been identified previously as efficient transduction enhancers.^[^ ^20^, ^21^ ^]^ These peptides are characterized by the presence of amphiphilic patterns composed of positively charged and lipophilic amino acids. In aqueous media, these peptides form very stable peptide aggregates. Several studies have confirmed that their positive net charge and microscopic aggregation is important for binding to negatively charged viral particles.^[^ ^20^, ^21^, ^22^, ^23^ ^]^ Subsequent attachment of the amyloid‐virus complexes to the cellular membrane leads to increased transduction rates.^[^ ^23^ ^]^ However, due to their intrinsic high stability, the in vitro biodegradation and removal of these often micrometer‐sized amyloid‐like fibrils is challenging. Therefore, introducing these fibrils along with the transduced cells into a patient's bloodstream may lead to undesired side effects, such as venous embolism. This poses significant challenges for their use in ex vivo therapy settings.^[^ ^24^ ^]^ Thus, successful peptide‐based adjuvants for clinical applications have to increase transduction efficiency and be easily removed or degraded after successful transduction. To date, no self‐assembling peptide has been reported that show these features.

Peptide amphiphiles (PAs) constitute a class of chimeric molecules that consist of a hydrophobic tail and a charged, hydrophilic peptide domain.^[^ ^25^, ^26^ ^]^ While different types of hydrophobic tails have been explored, fatty acid residues are most prevalent. The hydrophobic domain triggers self‐assembly and structure formation in water, which is then stabilized by the charged amino acid domains. In addition, certain amino acid sequences can give rise to ordered β‐sheet structures, which results in fibrillar nanostructures with high aspect ratios. Pioneering works by Stupp and coworkers developed and applied this material class for various medicinal applications.^[^ ^27^, ^28^, ^29^, ^30^, ^31^, ^32^ ^]^ In recent decades, the variety of peptide sequences employed in PAs has been greatly expanded,^[^ ^33^ ^]^ as well as their applications ranging from neural tissue regeneration^[^ ^34^ ^]^ to drug delivery,^[^ ^35^, ^36^ ^]^ antimicrobial systems,^[^ ^37^ ^]^ peptide vaccines,^[^ ^38^, ^39^ ^]^ immune‐therapeutic applications^[^ ^40^, ^41^ ^]^ and non‐viral gene delivery.^[^ ^42^, ^43^, ^44^ ^]^ For instance, by attaching a fatty acid residue to antibacterial^[^ ^37^, ^45^ ^]^ and antiviral peptides,^[^ ^46^, ^47^, ^48^, ^49^, ^50^, ^51^ ^]^ their potency was greatly improved. Recent works have shown that PAs are internalized via lipid‐raft mediated endocytosis by a variety of cell lines without affecting cell viability.^[^ ^52^, ^53^, ^54^ ^]^ Interestingly, the morphology of PA‐based nanostructures can be destroyed via enzymatic cleavage of the fatty acid from the peptide, which renders them inherently biodegradable.^[^ ^55^, ^56^, ^57^ ^]^ These unique features make PAs very attractive for ex vivo gene delivery applications. However, to the best of our knowledge, PAs have not been applied as an additive in the context of retroviral transduction.

Herein, for the first time, we propose PAs as highly efficient and biodegradable retroviral transduction enhancers. In our PA design, we disconnect the aggregate forming and membrane‐binding features of amyloid peptides. The PA peptide sequence comprises positively charged amino acids that support the interactions with the viral and cellular membrane required for high transduction efficiency of the retroviral particles, whereas self‐assembly is mainly driven by the palmitic acid residues, which should facilitate the formation of smaller aggregates with high in vitro degradability. Peptides prone to high aggregation are compared with peptides that reveal lower tendency for aggregate formation in view of their in vitro degradability. We report on PA1 (C_16_‐VVVAAAKKK‐NH_2_) and new peptide amphiphiles such as eicosapentaenoic acid‐VVVAAAKKK‐NH_2_, C_16_‐*thioester*‐CVVVAAAKKK‐NH_2_, C_16_‐*ester*‐SVVVAAAKKK‐NH_2_, C_16_‐ALAAGKK‐NH_2_, and C_16_‐AKAVGK‐NH_2_ which demonstrate a combination of in vitro degradability and high transduction efficacy. In fact, their effectiveness as transduction enhancers is comparable to the gold standard transduction enhancers RetroNectin, Vectofusin‐1 or Protransduzin (enhancing factor C, EF‐C).^[^ ^23^, ^58^, ^59^, ^60^ ^]^

## 2 Results and Discussion

### 2.1 Peptide Fibril Design

**Figure 1:**
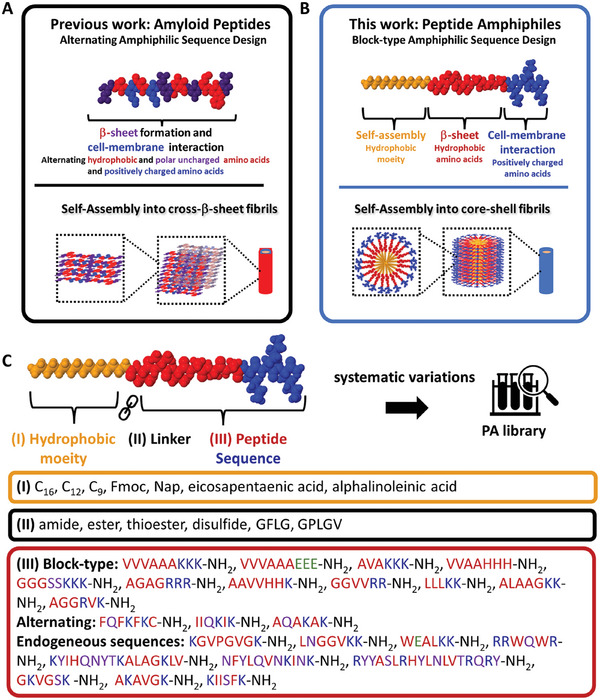
A Schematic illustration showing the sequence design of amyloid peptides (EF‐C, QCKIKQIINMWQ) previously reported for infectivity enhancement^[^ ^23^ ^]^ and B peptide amphiphiles (PA1, C_16_‐VVVAAAKKK‐NH_2_) reported in this study. Amyloid peptides have an alternating amphiphilic sequence that leads to self‐assembly into rigid, stable cross‐β sheet‐rich fibrils. In contrast, PAs self‐assemble based on hydrophobic fatty acids, resulting in core‐shell fibril structures. The β‐sheet secondary order is determined by non‐polar amino acids (red), while the cationic, polar residues (blue) are oriented outwards, making them interactive with virus particles and cell membranes. C Peptide amphiphile library created by systematic variations of the original sequence PA1 to study the effects of the (I) hydrophobic moiety, (II) the linkage chemistry and (III) the peptide sequence on infectivity enhancement and degradability. The peptide sequences were designed either according to a block‐type amphiphilic scheme, alternating amphiphilic, or derived from endogenous peptides not following any design scheme. Color code for sequence representation: Yellow hydrophobic moiety, blue cationic amino acids, red non‐polar amino acids, purple polar amino acids, green anionic amino acids.

Cross β‐sheet rich amyloid forming peptide transduction enhancers based on the enhancing factor C (EF‐C) are usually characterized by alternating amphiphilic patterns composed of hydrophilic (lysine) and aliphatic (isoleucine or valine) or aromatic (phenylalanine, tyrosine, tryptophan) amino acids (**Figure** **1**A).^[^ ^21^ ^]^ In order to design PA based nanofibrils that reveal (I) high viral transduction efficiency and (II) high in vitro degradability, the amphiphilic patterns were changed to a block‐type arrangement as depicted in Figure 1B.

**Figure 2:**
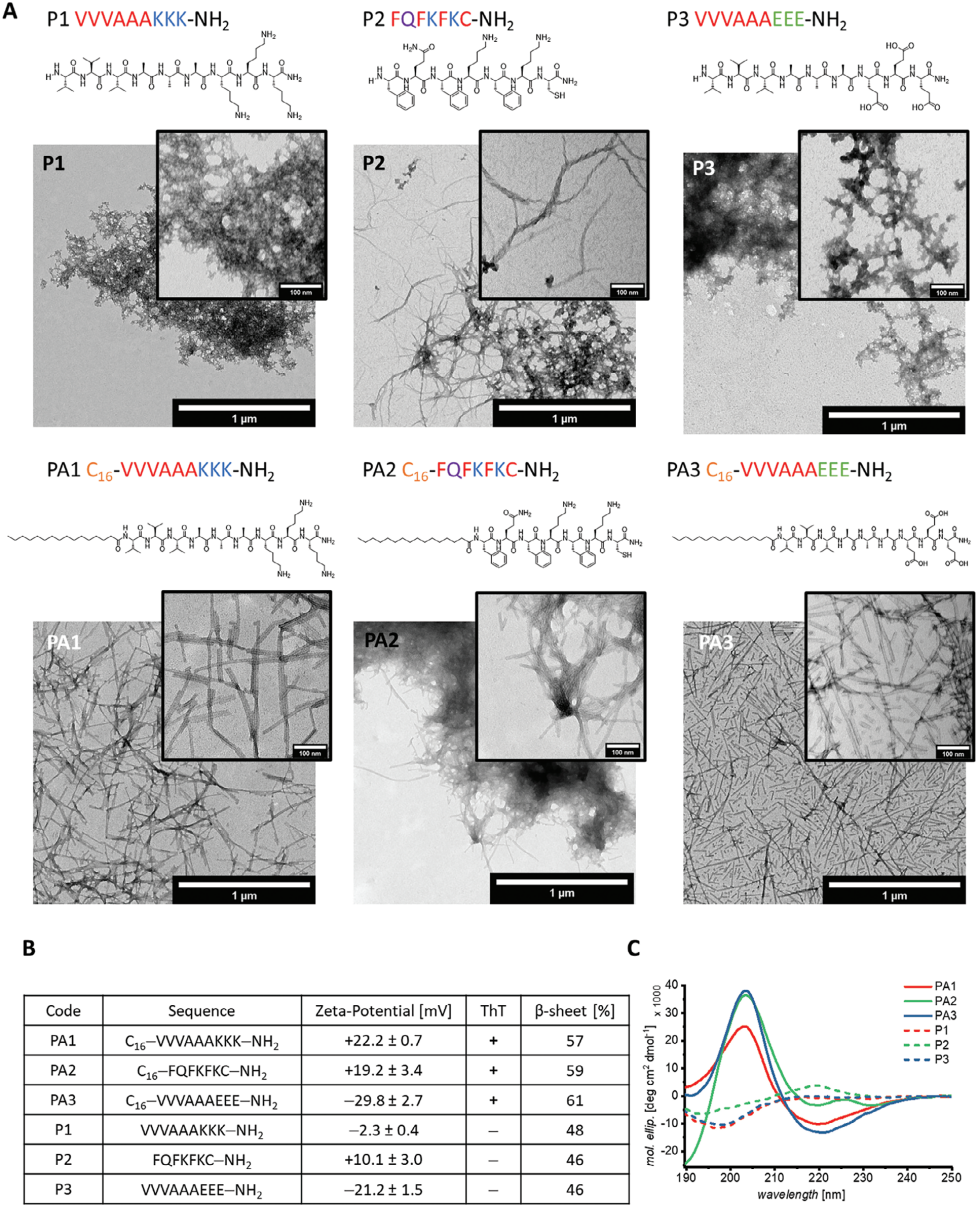
Characterization of PA1–PA3 and P1–P3. Color code for sequence representation: Yellow hydrophobic moiety, blue cationic amino acids, red non‐polar amino acids, purple polar amino acids, green anionic amino acids. A Representative TEM micrographs of P1–P3 and PA1–PA3 reveal the morphologies of peptide assemblies (1 mg mL^‐1^ in PBS). Formation of fibrils are observed for PA1–PA3 and P2, whereas P1 and P3 assemble in amorphous aggregates (scale bars 1 µm, inset: 100 nm). B Tabular summary of physicochemical features of PA1–PA3 and P1–P3 (1mg mL^‐1^, PBS) including zeta‐potential, ThT fluorescence (+ = ThT active, − = no ThT fluorescence observed). The β‐sheet contents were determined via a quantitative analysis by peak integration of parallel β‐sheet at 1630 cm^–1^ in the amide I region of the 2^nd^ derivative of ATR‐IR spectra (Figure S4). C CD spectra display β‐sheet secondary order for PA1–PA3 and random coil conformation for P1 and P3 (all peptides 0.1 mg mL^‐1^ in pure water).

The sequence design for block‐type PAs is derived from the non‐self‐assembling peptides VVVAAAKKK‐NH_2_ (P1) and VVVAAAEEE‐NH_2_ (P3) which contain β‐sheet‐prone hydrophobic amino acids sequence “VVVAAA” as well as positively “KKK” and negatively “EEE” charged residues at physiologic pH, respectively. Combining fatty acid chains with these peptides afford the conjugates C_16_‐VVVAAAKKK‐NH_2_ (PA1) and C_16_‐VVVAAAEEE‐NH_2_ (PA3). These PAs form nanofibers that have been extensively studied by Stupp and coworkers for a variety of applications.^[^ ^32^, ^61^, ^62^ ^]^ For comparison, we designed the self‐assembling peptide FQFKFKC‐NH_2_ (P2) with an alternating amphiphilic pattern based on transduction enhancers previously reported by us.^[^ ^20^ ^]^ Both FQFKFKC‐NH_2_ and C_16_‐FQFKFKC‐NH_2_ (PA2) readily form nanofibrils (**Figure** **2**A).

To cover a wide variety of different PA designs and study its influence on aggregate formation and degradation, we designed a library inspired by PA1 and PA2 consisting of 36 distinct peptide amphiphiles by varying the (I) hydrophobic moiety, (II) the linkage chemistry and (III) the peptide sequence (Figure 1C, SI chapter S7). Peptide synthesis was conducted via standard solid‐phase chemistry using the Fmoc protection strategy (see methods). PAs were accomplished by linking a palmitic acid (C_16_) to the *N*‐terminus according to a protocol described previously.^[^ ^62^, ^63^ ^]^ The purity of the conjugates (> 90%) was determined by LC‐MS or HPLC and the expected *m*/*z* values were determined by MALDI‐TOF for each peptide (Figure S1). After confirming the successful synthesis of the peptides, they were characterized by various methods to assess aggregation and fibril formation as discussed below (zeta‐potential, Thioflavin T (ThT) fluorescence, attenuated total reflection infrared (ATR‐IR) spectroscopy, and transmission electron microscopy (TEM)) (Figure 2, SI chapter S7, see methods).

### 2.2 Coupling Peptides to Palmitic Acid Enables Fibrillar Assembly

In the next sections, we will focus on the representatively selected examples PA1‐PA3 and the parent peptides P1‐P3, to illustrate their assembly, activity, and biodegradability, as we present only a portion of the PAs library for the sake of clarity and brevity. Detailed results and further discussion of the entire library can be found in SI chapter S7.

The block‐type amphiphilic peptides P1 and P3 without the palmitic tail formed non‐fibrillar amorphous aggregates, whereas the alternating amphiphilic sequence P2 assembled into twisted fibrils upon dilution from dimethyl sulfoxide (DMSO) stock solution in phosphate‐buffered saline (PBS), according to TEM images (Figure 2A). The fatty acid conjugates PA1 and PA2 assembled into distinct fibrils in water (Figure S2C‐D), whereas in PBS, micrometer‐sized clusters of fibrils were formed (Figure S3B‐C). Multivalent ions such as phosphate ions are known to interact with basic amino acid residues like lysine causing aggregation, probably through salt bridge formation between negative charged phosphate ions and positively charge lysine residues.^[^ ^64^, ^65^ ^]^ As expected, substituting lysine residues with negatively charged glutamic acid residues in PA3 results in distinct fibrils (Figure 2A), which did not bundle or adopt µm‐sized aggregates in PBS (Figure S3D).

Beside morphologic properties, we have previously identified positive zeta‐potential as an important factor for highly active transduction enhancers.^[^ ^20^ ^]^ The zeta‐potential characterizes the electrokinetic potential at the slipping plane of colloidal particles and can be measured for self‐assembling peptides if they form fibrils or amorphous aggregates. The peptide sequences of PA1 and PA2 contain lysine residues that are positively charged at pH 7.4. PA3 however, provides negatively charged glutamic acids. As expected, PA1 and PA2 displayed a positive zeta‐potential while PA3 showed a negative zeta‐potential (Figure 2B). Compared to PA1–PA3, the parent peptides P1–P3 displayed a less positive zeta‐potential despite having the same peptide sequence. This observation underlines that the zeta‐potential is not only dependent on the charged groups within the peptide sequence, but may also be influenced by the size and morphology of the assembled structure, which was also previously shown for other PAs.^[^ ^66^, ^67^ ^]^

To gain information on the secondary structure of the assembled peptides, ATR‐IR measurements were conducted with a focus on the amide I (1600–1700 cm^‐1^) and amide II (1500–1600 cm^‐1^) regions.^[^ ^68^, ^69^ ^]^ Interestingly, β‐sheet structural elements were identified for all peptides P1–P3 and PA1–PA3 with a main peak around 1630 cm^‐1^, which is assigned to parallel β‐sheets (see SI chapter S3). A quantitative analysis of the amide I region revealed that PA1–PA3 show a 9–15% higher β‐sheet content compared to their respective parent peptides P1–P3 (Figure 2B, Figure S4). The secondary structure of the PAs is further confirmed in solution via circular dichroism (CD) spectroscopy (Figure 2C) and ThT fluorescence (Figure 2B, Figure S5). Interestingly, P1–P3 show CD spectra with characteristics of a random coil structure and no β‐sheet structure, which can be traced back to the preparation in pure water lacking counter‐ions, which are likely facilitating intermolecular interaction via charge screening of charged residues “K” and “E” (SI chapter S2). By introducing palmitic acid in PA1–PA3, a predominant β‐sheet structure is observed (Figure 2C). The CD spectra of PA1 and PA3 are in good agreement with literature reports.^[^ ^62^, ^70^ ^]^ The fluorescence of ThT upon excitation at 488 nm can only be detected if the molecular rotation of the dye is hindered, that is upon binding to highly ordered structures such as amyloid or β‐sheet containing fibrils. As expected, ThT fluorescence could be observed for β‐sheet fibril‐forming PA1–PA3 but not for P1–P3 (Figure 2C and Figure S5). Therefore, only PA1 and PA3 form fibrils with high β‐sheet content in PBS, which was not observed for the parent peptides P1 and P3 without the palmitic acid moiety. In our PA library we found that 21 of 23 tested PAs with newly designed peptide sequences assembled into β‐sheet rich fibrils and conclude that attaching palmitic acid to the *N*‐terminus of non‐assembling peptides is a reliable way to create self‐assembling PAs (Figure S19, Figure S27). Thus, the introduction of the fatty acid residue drives the assembly of the peptides into ordered β ‐sheet rich nanostructures as reported,^[^ ^71^ ^]^ which is required for boosting retroviral gene transduction.

### 2.3 Sequence Hydrophilicity and Arrangement are Important for Aggregation

In general, amyloid fibrils with high β‐sheet content are usually degraded slowly by cells.^[^ ^72^ ^]^ However, PAs can be internalized by cells^[^ ^73^, ^74^ ^]^ which may be facilitated by interactions of the fatty acids with cellular membranes.^[^ ^52^, ^75^ ^]^ As assemblies of PAs are likely degraded by cleavage of the fatty acid residue in cells,^[^ ^52^, ^53^, ^55^, ^76^ ^]^ we hypothesized that the difference in biodegradability can be traced back to (I) the size of the initial aggregates and (II) the different self‐assembling properties and biodegradability of the parent peptides.

**Figure 3:**
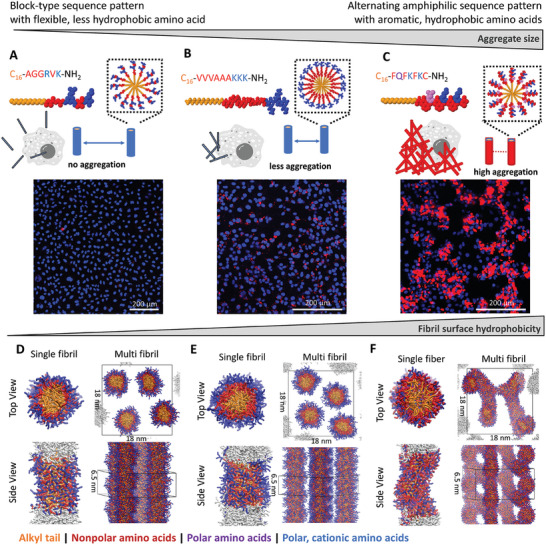
Schematic representation of three selected PAs A C_16_‐AGGRVK‐NH_2_, B PA1, and C PA2, that show distinct aggregation properties. Laser scanning microscopy images of Proteostat stained PAs (red) and HeLa cells (nucleus in blue) reveal formation of µm‐sized PA clusters for PA1 and PA2. The PAs (20 µg mL^‐1^) were added to cells for 30 min, washed and samples were analyzed. PA1 and PA2 revealed association with cellular membranes while C_16_‐AGGRVK‐NH_2_ did not associate with cells, scale bar 200 µm. D – F Coarse grained molecular dynamics simulations. Snapshots show cross‐sectional (top) and side views of selected PAs self‐assembled into single fibers after 15 µs from randomly dispersed PAs in solution in a simulation box with a size 13 nm × 13 nm along the fibril cross‐section (top view) and 6.5 nm along the lateral direction (side view). The snapshots for the interaction of multiple fibers are shown after self‐assembly following an additional 10 µs simulation time in a box with a size 18 nm × 18 nm along the fibril cross‐section (top view) and 6.5 nm along the lateral direction (side view). Color codes represent alkyl chains in yellow, nonpolar amino acids in red, polar amino acids in purple and polar, cationic amino acids in blue. Water and ions in the simulation box are omitted in the snapshots for clarity. For the single fibril system, the molecules in the simulation box are shown in color, while the periodic image is shown in white. The simulation box is shown in black for the multi fibril system. D Simulation of C_16_‐AGGRVK‐NH_2_ and E PA1 reveal stable fibril formation with high‐density of polar groups (blue) on the fibril surface that prevents fibril‐fibril interaction. F In contrast, PA2 displays hydrophobic amino acids (red) on the fibrils surface that is likely the main driving force for multi‐fibril networks formation.

Our observations reveal three different aggregation types in the PA library: fibers that do not aggregate or attach to cells (*e.g*., C_16_‐AGGRVK‐NH_2_, **Figure** **3**A), fibers that form small µm‐sized aggregates that attach to cells (e.g., PA1 Figure 3B), and fibers that form large µm‐sized aggregates that attach to cells (*e.g*., PA2 Figure 3C).

The aggregation of PAs is a complex process that occurs at various length scales, from molecular self‐assembly dynamics to fiber‐fiber interactions and micrometer‐sized colloid formation. This makes it challenging to identify specific molecular design rules for PAs as a driving force behind microscopic aggregation. However, findings from our systematic PA library (SI chapter S7.3) suggest that fibrils that do not aggregate share a common feature: they consist of a high number of less hydrophobic, flexible (A, G) and polar amino acids (R, K, E) close to the fatty acid (Figure 3A). This could increase dynamicity and solvation of the self‐assembling PAs, which results in less aggregating fibers. PAs with a block‐type arrangement of hydrophobic (V, I) and hydrophilic, charged (K, R) amino acids, as found in PA1, form small‐sized aggregates (Figure 3B). In contrast, strongly aggregating fibrils consist of high numbers of hydrophobic aromatic amino acids and an alternating amphiphilic sequence order as found in PA2 (Figure 3C).

From these observations, we hypothesized that surface hydrophobicity of the assembled fibrils could be a key feature that determines their stability and aggregation. To evaluate this hypothesis, we performed coarse‐grained (CG) molecular dynamics simulations on three peptide amphiphiles that show distinct aggregation behavior: C_16_‐AGGRVK‐NH_2_, PA1, and PA2 (Figure 3D – F).

In the first step, we studied the self‐assembly of PAs in solution from randomly dispersed molecules to fibrils using CG Martini model (SI chapter S13).^[^ ^77^, ^78^ ^]^ After attaining stable equilibrium structures in under 15 µs, we further investigated fibril‐fibril interactions by placing fibrils in close proximity in solution to gain insight into the different degrees of aggregation observed in experiments. Although the Martini model has limitations, such as determining the evolution of peptide secondary structure, in this case, we are not interested in the secondary structure of peptide amphiphiles but in the aggregation behavior to make connections with experiments.

In line with experimental findings, our simulations show differences in the arrangement of amino acid residues in the core and on the surface of the fibrils depending on the PA sequence. For example, while the block‐type PA1 forms distinct domains such as the hydrophobic core and the hydrophilic surface of the fibrils (Figure 3E), PA2's alternating sequence allows for less homogeneous distribution of the amino acids on the surface. While the fibrils are stabilized by the hydrophobic tail, we observe the presence of not only hydrophilic but also hydrophobic (phenylalanine and cysteine) residues surface‐exposed and in contact with solvent molecules (Figure 3F). This, in turn, allows for a more attractive interaction between fibrils in solution, which aggregate to form connecting networks of “supramolecular cross‐links” between the fibrils. In contrast, PA1 and C_16_‐AGGRVK‐NH_2_ (Figure 3D) show very little aggregation in solution. This is qualitatively reflected in fiber‐fiber distance (Figure S34) and solvent accessible surface area (SASA, Figure S35A) in SI chapter S13.2.

The aggregation behavior also affects the mobility of the supramolecular components measured by root mean square fluctuations (RMSF) of residues, where increased fibril‐fibril aggregation in PA2 results in less mobile fibrils (Figure S36). Other non‐aggregating fibrils, such as PA1 and C_16_‐AGGRVK‐NH_2_ also remain stable but show larger movements in response to interactions with solvent and other fibrils in solution (SI chapter S13.3). Noteworthy the RMSF changes for PA1 depending on whether single (Figure S36A) or multiple fibers (Figure S36B) are considered, which indicates that it is important to not only model the self‐assembled fiber but also the inter‐fiber interactions for evaluation of fibril properties.

Peptides with an alternating amphiphilic sequence order and high content of hydrophobic amino acids are commonly found in amyloidogenic peptides.^[^ ^21^ ^]^ Our findings suggest that these properties can also promote intermolecular and fiber‐fiber interactions of PAs, leading to increased colloidal aggregation.

### 2.4 PAs – Forming Small Aggregates are Degraded Efficiently

**Figure 4:**
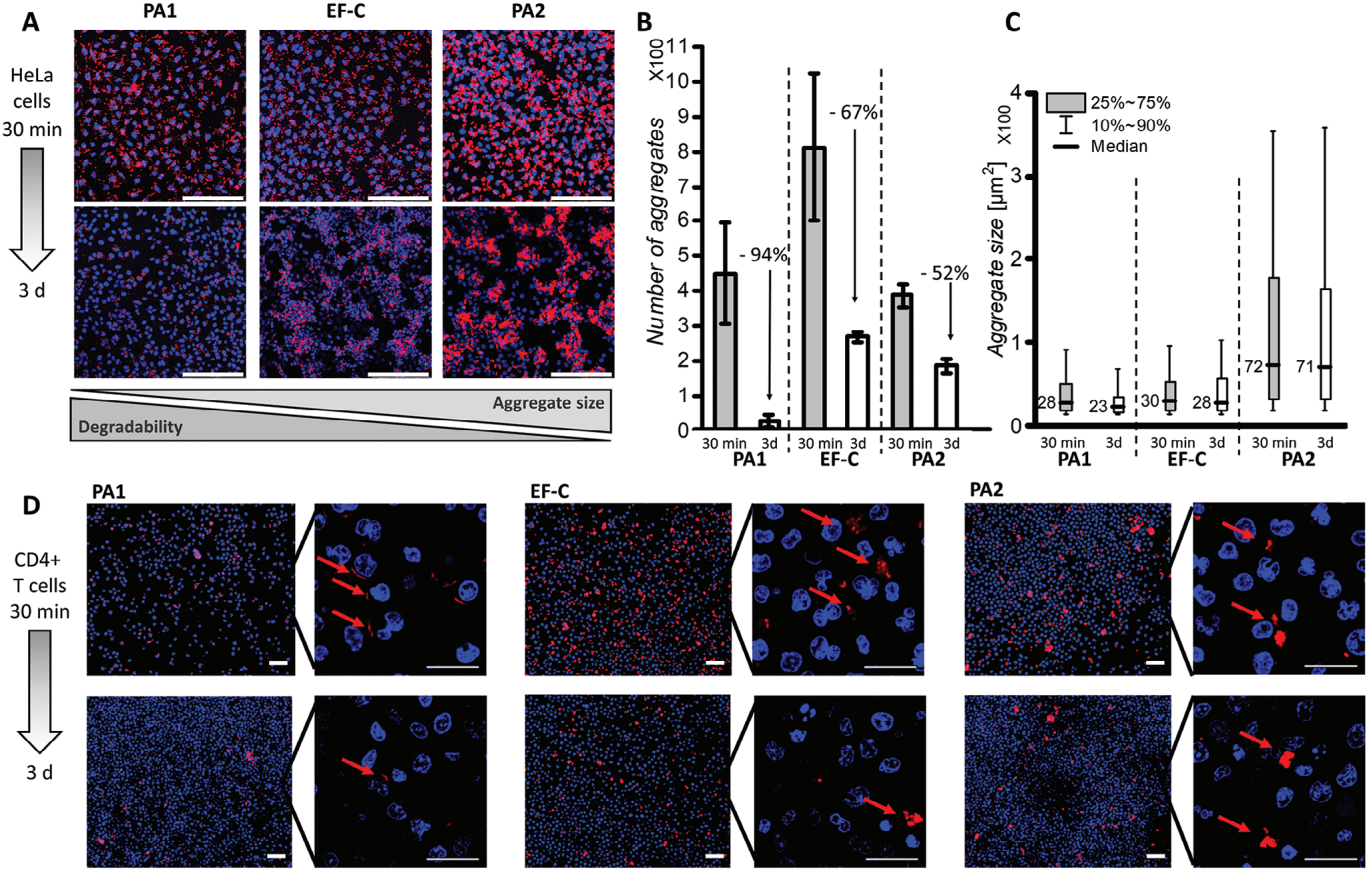
Degradability of µm‐sized fibrillar aggregates formed by EF‐C, PA1 and PA2 after 30 min and 3 d in HeLa and CD4+ T cell culture. **A** Representative laser scanning microscopy images of Proteostat‐labeled EF‐C, PA1 and PA2 (red) incubated with HeLa cells (nucleus in blue). In general, PA1 reveals less and smaller µm‐sized aggregates than PA2 and EF‐C. After 3 days in cell‐culture, almost all small aggregates of PA1 are degraded, whereas aggregates formed by PA2 stay almost unchanged, scale bar 200 µm. **B** Bar plot showing number of aggregates > 10 µm^2^ in an area of 581.25 µm × 581.25 µm in cell culture after 30 min and 3 d incubation (4 µg peptide added to 40,000 cells), error bars indicate standard deviations from six technical replicates. **C** Box plot showing size distribution of aggregates in an area of 581.25 µm × 581.25 µm in cell culture after 30 min and 3 d incubation (4 µg peptide added to 40,000 cells), deviations indicate three technical replicates. **D** Representative laser scanning microscopy images of Proteostat‐labeled EF‐C, PA1 and PA2 (red) incubated with CD4+ T cells (nucleus in blue). PA1 reveals less and smaller µm‐sized aggregates that attach as thin layers to cell membrane compared to PA2 and EF‐C (indicated by red arrows). After 3 days in cell culture, almost all small aggregates of PA1 are degraded by CD4+ T cells (1 µg peptide added to 250,000 cells), scale bar 20 µm.

**Figure 5:**
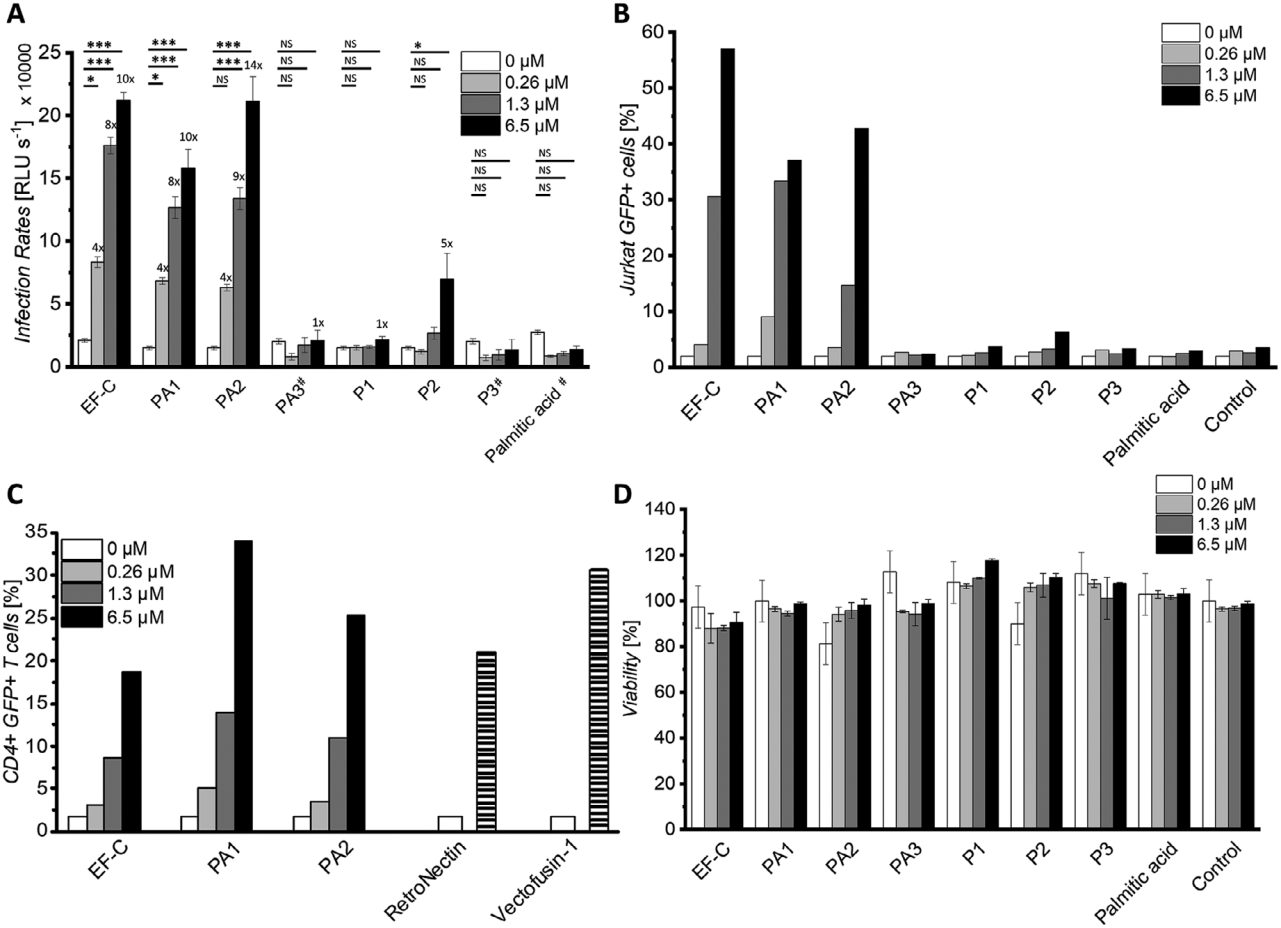
Peptide amphiphiles PA1 and PA2 are potent enhancers of HIV‐1 infection and γ‐retroviral transduction. A Infectivity assay showing HIV‐1 infection rates of TZM‐bl cells observed in the presence of increasing concentrations of peptides PA1–PA3 and P1–P3 (0.26 µM, 1.3 µM, 6.5 µM). The baseline (0 µM) shows infection rate without the addition of peptides. Values above each bar represent the n‐fold enhancement compared to 0 µM. Mean values and standard deviations are derived from three repetitions of triplicate infections. Two repetitions of triplicate infections were used for samples marked with (^#^). P‐values for statistical significance were determined by two‐way ANOVA test with Dunnett's multiple comparison test and are indicated with * p ≤ 0.05, ** p ≤ 0.01, *** p ≤ 0.001, NS not significant, RLU s^‐1^ are relative light units per second. B Percentage of green fluorescent protein (GFP) positive Jurkat cells at indicated concentrations of peptides three days after transduction with a gibbon ape leukemia virus pseudotyped γ‐retroviral vector (GALV‐RV). Experiment has been performed once. C Percentage of GFP positive CD4+ T cells after transduction with GALV‐RV. Comparing the transduction efficiency enhancement of EF‐C, PA1 and PA2 (0.26 µM, 1.3 µM, 6.5 µM, for EF‐C 6.5 µM equals 10 µg mL^‐1^) with benchmark retroviral transduction enhancers RetroNectin (20 µg mL^‐1^, 0.3 µM) and Vectofusin‐1 (10 µg mL^‐1^, 3.3 µM) as recommended by the manufacturers. Experiment has been performed once. D CellTiter‐Glo cell viability assay of Jurkat cells treated with increasing concentrations of the peptides of biological triplicate with standard deviation.

To assess how the aggregate size affects the biodegradability of the PAs, the number of aggregates > 10 µm^2^ were evaluated via confocal microscopy in the presence of HeLa cells after 30 min and 3 d of incubation at 37°C (SI chapter S7). This time interval was selected, because the corresponding infectivity assays are always performed for at least 3 d. Most of the peptide nanostructures such as PA1 and PA2 investigated in this work as well as other commercially available peptides for transduction efficiency enhancement such as EF‐C,^[^ ^23^ ^]^ Vectofusin‐1^[^ ^79^ ^]^ form µm‐sized aggregates (**Figure** **4**A, Figure S6). While aggregate formation can be an obstacle in clinical applications^[^ ^24^ ^]^ it does not appear to affect cell viability (**Figure** **5**D, Figure S7, Figure S25). To avoid possible complications upon re‐administration of, e.g., transduced T‐cells, the reduction of aggregates (> 10 µm^2^) is highly desired for clinical applications.^[^ ^80^, ^81^ ^]^ We found that 16 of the 36 investigated PAs could be degraded by cells (SI chapter S7, Figure S12). For example, PA1 forms less and smaller aggregates compared to EF‐C and PA2, which could be well degraded (94%) over the course of 3 days in the presence of HeLa cells (Figure 4A,B,C, Figure S8) as well as CD4+ T cells (Figure 4D, Figure S9). In contrast, PA2 forms larger µm‐sized aggregates, which remain nearly unchanged in size after 3 days incubation and are not degraded as efficiently (52%) in presence of HeLa cells (Figure 4B, Figure S8) and CD4+ T cells (Figure 4D, Figure S9). The efficient cellular biodegradation for PA1 is further emphasized in control experiments without cells where PA1, PA2 and EF‐C are not significantly changing in size and number over the course of 3 days (Figure S10).

In contrast to P1, P2 forms fibrils (Figure 2A). Thus, after cleavage of the fatty acid, fibril formation and microscopic aggregation of P2 could hinder degradation as observed here. To examine how peptide sequence biodegradability impacts overall PA degradability, we designed pa1, which is the d‐amino acid equivalent of PA1 (Figure S11). All physicochemical properties of pa1 remain similar to those of PA1. However, fibrillar aggregates of pa1 cannot be degraded by HeLa cells within a 3‐day timeframe. Thus, the reduced biodegradability for pa1 can be attributed directly to sequence degradability, specifically the inability of cells to degrade d‐amino acids.

We conclude that PA1 forms smaller aggregates and it is degraded more quickly in vitro, which distinguishes them from amyloid‐like peptide fibrils, e.g. EF‐C or PAs consisting of alternating amphiphilic amyloid‐peptide sequences such as PA2, which show in general higher in vitro stability (Figure 4, Figure S7).^[^ ^82^, ^83^ ^]^ PA1 is much better degraded compared to PA2 because of non‐assembling parent peptide sequence and the small size of aggregated fibrils.

### 2.5 Positively Charged PAs are Potent Enhancers of γ‐Retroviral Transduction

Finally, we evaluated the capacity of the PA library to enhance HIV‐1 infection of TZM‐bl cells, which is a well‐established and frequently applied model to quantifying HIV‐1 infectivity.^[^ ^84^ ^]^ EF‐C (QCKIKQIINMWQ), which has been reported earlier by us,^[^ ^20^ ^]^ served as a reference. EF‐C is a highly potent enhancer on par with other commercially available transduction enhancers such as RetroNectin and Vectofusin‐1 serving as gold standards for transduction enhancers.^[^ ^23^, ^79^ ^]^

Out of the 36 PAs in our library, 21 showed a significant increase in infectivity by at least 2‐fold compared to the virus alone at a concentration of 1.3 µM (Figure S12). This confirms the effectiveness of adding fatty acids to the N‐terminus to transform the inactive parent peptide sequences (Figure S23) into ones that enhance infectivity (Figure S19). Inactive PAs lack the physicochemical properties which were reported earlier by us as important requirements for infectivity enhancement.^[^ ^21^ ^]^ They either cannot self‐assemble into fibrils (e.g., C_16_‐KGVPGVGK‐NH_2_, alpha linolenic acid‐VVVAAAKKK‐NH_2_, C_16_‐WEALKK‐NH_2_), cannot form µm‐sized aggregates (*e.g* Nap‐VVVAAAKKK‐NH_2_, C_16_‐GGGSSKKK‐NH_2_, C_16_‐AGAGRRR‐NH_2_, C_16_‐GGVVRR‐NH_2_, C_16_‐RRWQWR‐NH_2_, C_16_‐GKVGSK ‐NH_2_, SI chapter S7) or have a negative zeta‐potential (PA3, Figure 5A). In accordance with the above demonstrated aggregate analysis, PAs which cannot form fibrillar aggregates contain less hydrophobic amino acids (G, A), more flexible hydrophobic moieties (alpha linolenic acid, naphthalene) or charged amino acids (R, K, E) close to the hydrophobic moiety.

PA1 and PA2 are amongst the most active PAs and show a strong infectivity enhancement of HIV‐1 in the range of EF‐C (Figure 5A). Our data indicate a similar mechanism for the activity as reported previously for EF‐C.^[^ ^23^ ^]^ The positively charged fibrillar aggregates of PA1 and PA2 interact with negatively cellular membranes of HeLa cells as shown by confocal fluorescence microscopy measurements depicted in Figure 4A and Figure S6. Importantly, the parent peptides P1 and P3 without fatty‐acid residues are inactive (Figure 5A) and did not interact with the cellular membrane (Figure S6). These findings demonstrate that even inactive peptides can be rendered active when a fatty acid residue is introduced and induces self‐assembly into fibrils with high β‐sheet content. P2 with the alternating amphiphilic sequence design can assemble into moderately positive charged fibrils (Figure 2A,B) and thus enhances infectivity even without the fatty acid residue (Figure 5A). The addition of a fatty acid moiety to peptides can promote the formation of β‐sheet rich fibrils, which in turn increases their transduction enhancing activity, given that the overall charge is positive.

To investigate the potential use for gene therapy applications we evaluated the effect of P1–P3 and PA1–PA3 on the transduction of Jurkat cells and clinically relevant primary T cells. First, we tested Jurkat cells, a T‐cell line frequently applied for studying leukemia,^[^ ^85^ ^]^ with a γ‐retroviral vector pseudotyped with the glycoprotein derived from gibbon ape leukemia virus (GALV‐RV). Without a PA additive, almost no transduced GFP+ cells were detected (Figure 5B). However, with an increasing concentration of PA1 and PA2 strong transduction enhancement similar to EF‐C was achieved. Next, PA1 and PA2 were tested in a more clinically relevant transduction setting. To this end, primary CD4+ T cells were isolated, activated and transduced with GALV‐RV in presence of different transduction enhancers. Strikingly, PA1 and PA2 showed transduction efficacies in the range of commercially available gold‐standard enhancers RetroNectin and Vectofusin‐1 (Figure 5C), which were utilized as recommended by the manufacturers, i.e., time‐consuming coating and washing steps in the case of RetroNectin. Thus, PAs are powerful transduction enhancers with similar transduction enhancing efficacies as commercially available systems. Furthermore, PA1 and PA2 did not reduce the viability of Jurkat cells (Figure 5D), or CD4+ T cells (Figure S7), which is important in terms of CAR‐T cell therapy.

**Figure 6:**
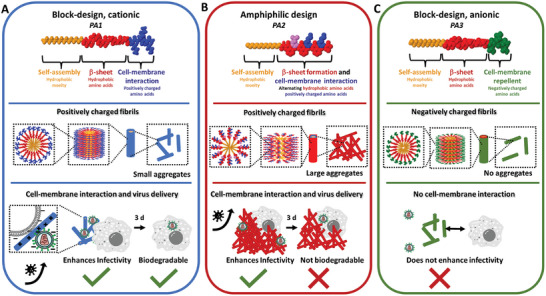
Schematic illustration of main findings in this study. A PAs with block‐type design of hydrophobic and cationic amino acids (e.g. PA1, C_16_‐VVVAAAKKK‐NH_2_) form moderately sized aggregates that can attach to cellular membranes, deliver viruses and are biodegradable in the timeframe of 3 d. B PAs with an alternating amphiphilic sequence design with hydrophobic amino acids (e.g. PA2, C_16_‐FQFKFKC‐NH_2_) form large aggregates (> 50 µm^2^) that can deliver viruses but cannot be efficiently biodegraded. C PAs containing anionic amino acids (e.g. PA3, C_16_‐VVVAAAEEE‐NH_2_) hinder cellular attachment and are not suitable for retroviral gene delivery.

In summary, PAs that have block‐type sequence design with hydrophobic and cationic charged amino acids, i.e., in PA1 form moderately sized aggregates that are attaching to cellular membranes, deliver viruses and are biodegradable (**Figure** **6**A). Replacing cationic amino acids with anionic amino acids, i.e., in PA3 prevent cellular attachment and are not suitable for retroviral gene delivery (Figure 6C). Changing the sequence design from block‐type to an alternating amphiphilic sequence design with aromatic, hydrophobic amino acids results in PAs that form large aggregates (> 50 µm^2^, i.e., PA2), which can increase virus uptake but that cannot be fast and efficiently degraded by the cells (Figure 6B). While a certain degree of aggregation is required (SI chapter S7) to efficiently interact with cells and viruses, the size of the aggregates is crucial to facilitate biodegradability.

## 3 Conclusion

In this study, we present peptide amphiphiles (PAs) as biodegradable additives for promoting retroviral transduction. The conjugation of fatty acid residues can render inactive peptide sequences active as demonstrated by evaluating 36 different PAs in a virus infection assay representing a promising approach to discover new PAs for enhancing retroviral transduction on a par with established materials like RetroNectin, Vectofusin‐1 and EF‐C. Active PAs showed the characteristic ability to form β‐sheet rich fibrous aggregates with a positive zeta‐potential making them highly potent transduction enhancers. The arrangement and hydrophilicity of amino acids in the peptide sequence plays a crucial role in controlling the aggregate morphologies and biodegradability of the peptide amphiphiles (PAs). We could demonstrate via molecular dynamics simulations that fibril‐fibril aggregation of PAs could be traced back to the fibril surface hydrophobicity and self‐assembly dynamics, with fibrils consisting of more mobile PAs causing less aggregation. Specifically, block‐type amphiphilic peptide motifs afford PAs forming small aggregates that can be efficiently degraded in the presence of cells, while alternating amphiphilic peptide sequences yield PAs with high tendency to form large aggregates that are not degraded by cells within the relevant time of the transduction experiment.

In summary, we have introduced PAs with a block‐type sequence arrangement as a novel class of transduction enhancers. These PAs share key physicochemical traits with previously reported amyloid‐like self‐assembling peptides, but they reveal excellent biocompatibility and degradability. We envision that this class of transduction enhancers could outperform conventional amyloid‐like peptides in future ex vivo clinical gene transfer applications.

## 4 Experimental Section

### Materials

For peptide synthesis, Rink Amide AM resin LL (100‐200 mesh), OxymaPure, (2‐(1*H*‐benzotriazol‐1yl)−1,1,3,3‐tetramethyluronium hexafluorophosphate (HBTU), Fmoc‐Cys(Trt)‐OH, Fmoc‐Gln(Trt)‐OH, Fmoc‐Val‐OH, Fmoc‐Ala‐OH and Fmoc‐Glu(OtBu)‐OH were purchased from Novabiochem, Merck. Fmoc‐Lys(Boc)‐OH, Fmoc‐Phe‐OH, Piperidine, Trifluoroacetic acid, *N,N*‐Di*iso*propylethylamine and *N,N*‐Diisopropylcarbodiimide were purchased from Carl Roth GmbH + Co. KG. Palmitic acid and Triisopropylsilane were purchased from TCI Tokyo Chemical Industry. Peptide‐synthesis‐grade solvents (Dichloromethane, Dimethylformamide) were acquired from Acros Organics. Solvents were purchased in HPLC grade purity or higher from Sigma‐Aldrich (Diethyl ether, Dimethylsulfoxide (DMSO)), Fisher Scientific (Dichloromethane, Dimethylformamide, Acetonitrile). Peptide EF‐C (>95% purity) were purchased from Zhengzhou Phtd Peptides industrial Co. Vectofusin‐1 was purchased from Miltenyi Biotech and RetroNectin was purchased from Takara Bio. Proteostat was purchased from Enzo Life Sciences. Thioflavin‐T, Uranyl acetate and Dulbecco's Phosphate Buffered Saline without Magnesium and Calcium (DPBS) were purchased from Sigma‐Aldrich. Dulbecco's Modified Eagle Medium (DMEM), Roswell Park Memorial Institute (RPMI) 1640 Medium, fetal calf serum (FCS), Dynabeads Human T‐Activator CD3/CD28 were purchased from Gibco (Germany) was purchased from Sigma‐Aldrich. Penicillin‐Streptomycin and L‐Glutamine (200 mM) were purchased from PAN‐Biotech. Human interleukin‐2 (IL‐2), interleukin‐7 (IL‐7) and interleukin‐15 (IL‐15) were purchased from Miltenyi Biotech. RosetteSep Human CD4+ Enrichment Cocktail was purchased from STEMCELL Technologies.

### Instruments

Peptides were synthesized with an automated microwave peptide synthesizer (CEM, Liberty BlueTM). High pressure liquid chromatography (HPLC) on preparative scale was conducted with a Shimadzu system equipped with the following modules DGU‐20A5R, LC‐20AP, CBM‐20A, SPD‐M20A, SIL‐10AP, FRC‐18A with a reverse phase column Phenomenex Gemini (5 µm NX‐C18, 110 Å, 150×30 mm). Analytical scale HPLC was performed using a Shimadzu system with modules DGU‐20A5R, LC‐20AT, CBM‐20A, SPD‐M20A, SIL‐10ACHT, CTO‐20AC, column Zorbax XDB‐C18, 9.4×250 mm, 5 µm pore size. LC‐MS was conducted on a Shimadzu LC‐2020 Single Quadrupole MS instrument with the modules LC‐20AD, SIL‐20ACHT, SPD‐20A, CTO‐using a Kinetex EVO and C18 100 Å LC 50 × 2.1 mm column with 2.6 µm pore size. MALDI‐TOF‐MS was measured on a Bruker rapifleX MALDI‐TOF/TOF and a Waters MALDI Synapt G2‐SI instrument. TEM measurements were performed on a JEOL 1400 instrument with 120 kV acceleration voltage equipped with a CCD camera. Fluorescence intensity was measured using a Spark 20 M microplate reader by the company Tecan Group, Ltd. Zeta‐potential measurements were conducted with a Zetasizer Nano ZS (Malvern Instruments) with 1 mL disposable folded capillary cells. FT‐IR spectra of solid samples using a Bruker Tensor II spectrometer equipped with a diamond crystal as ATR element with a spectral resolution of 2 cm–1, each spectrum was an average of 64 scans. Circular dichroism measurements were conducted on a JASCO 1500 instrument. Fluorescence microscopy measurements were recorded on a Leica DMi8 microscope with 10x air objective and equipped with a Leica MC170 HD camera. Confocal laser scanning microscopy was performed on a Stellaris 8 confocal laser scanning microscope (Leica) equipped with a 20x air objective or on a Zeiss LSM 710 confocal microscope equipped with a 20x and 63x objective. Luminescence measurement for the evaluation of infection efficiency was conducted on Orion microplate luminometer (Berthold). Flow cytometry was conducted on CytoFLEX LX Flow Cytometer (Beckman Coulter) and flow cytometry data were analyzed using FlowJo (BD Bioscience).

### Synthesis and Purification of Peptides

Peptides (PA1‐3 and P1‐3) were synthesized using an automated microwave peptide synthesizer (CEM, Liberty BlueTM) from C to N‐terminus according to fluorenylmethyloxycarbonyl (Fmoc) solid phase peptide synthesis (SPPS) strategy by Merrifield using Rink Amide AM Resin LL (100‐200 mesh, Novabiochem). The beads were swollen in DMF while shaking the reaction vessel for 1 h. The Fmoc‐protecting group was cleaved off by a piperidine solution (20% in DMF) at 155 W, 75°C for 15 s and at 30 W, 90°C for 50 s. The beads were washed three times with DMF before the addition of the Fmoc‐protected amino‐acid (2 M, 5 equiv. relative to the resin loading capacity) in DMF, DIC (5 equiv.) in DMF and Oxyma Pure (10 equiv) in DMF. The coupling was conducted by microwaving at 170 W, 75°C for 15 s and at 30 W, 90°C for 110 s, the solution was removed, and the beads were washed with DMF. This procedure was repeated for each amino acid. Coupling of the fatty acid chain was conducted according to a previous reported literature procedure^[^ ^62^, ^63^ ^]^ by adding palmitic acid (4 molar equiv), DIPEA (6 molar equiv), HBTU (4 molar equiv) in DMF/DCM (5/1, v/v) at rt and shaking for 2 h. Cleavage from the resin was carried out in TFA/water/tri*iso*propylsilane (95/2.5/2.5, v/v/v) for 2 h at room temperature. This solution was precipitated in cold diethyl ether (40 mL) and centrifuged 3 times at 4°C, 4000 rpm for 15 min. The precipitate was dissolved in water with 0.1% TFA for peptides with lysine residue and with 0.1 % NH_4_OH for peptides with glutamic acid residues yielding PAs as colourless powders (PA1 34%, PA2 25%, PA3 46% yield). The purification was conducted via HPLC (reverse phase column Phenomenex Gemini 5 µm NX‐C18, 110 Å, 150×30 mm) using a gradient of water and acetonitrile containing 0.1 % TFA for peptides with lysine residues or 0.1 % NH_4_OH for peptides with glutamic acid residues as the mobile phase at a flow rate of 25 mL min^−1^. Chromatography was monitored with an UV absorption detector at 190 and 254 nm. The peptides were identified via MALDI‐TOF‐MS and purity was confirmed via LC‐MS or analytical HPLC as described in an previous report.^[^ ^86^ ^]^ The data was processed with Shimadzu LabSolutions and OriginLab. The PA and peptide library (SI chapter S7–12) were commercially obtained from GenScript Inc. > 95% purity except of C_16_‐*ester*‐SVVVAAAKKK‐NH_2,_ C_16_‐*disulfide*‐CVVVAAAKKK‐NH_2,_ C_16_‐*thioester‐*CVVVAAAKKK‐NH_2, and_ Eicosapentaenoic acid – VVVAAAKKK‐NH_2_ which were purchased from Bachem AG > 87% purity.

### Fibril Formation

The peptides were predissolved in DMSO to a concentration of 10 mg mL^−1^. The fibril formation was induced by further diluting the solution to a final incubation concentration of 650 µM or 1 mg mL^−1^ in PBS as indicated in the text and incubated for 1 d at RT. Other concentrations described in the text were achieved by further dilution of preformed fibrils.

### Characterization of Fibrils

Transmission electron microscopy (TEM) measurements, Thioflavin (ThT), zeta‐potential measurements for fibril characterization, ATR‐IR spectra acquisition and the calculation of β−sheet were prepared and conducted analogous to a previous report.^[^ ^20^, ^86^ ^]^ The morphology of the peptide assemblies was evaluated via TEM to determine the self‐assembly at the nanoscale into fibrillar structures or amorphous aggregates. Amorphous aggregates lack a fibrillar structure and have less internal order.^[^ ^87^, ^88^, ^89^ ^]^ Fluorescence microscopy of dye‐stained peptides was applied to distinguish between microscopically aggregating or distinct fibrils. Fibrils were defined as distinct, if no visible structures could be identified in fluorescence and brightfield microscopy. A peptide fibril was considered as ThT‐active if the fluorescence intensity was at least twice as strong compared to the control (PBS containing 10% DMSO). The derived count rate of the zeta‐potential measurement was used as information on the light scattering intensity and turbidity of the sample as a further indicator for microscopic aggregation.^[^ ^90^ ^]^

The experimental procedure is briefly described below.

TEM: 5 µL of fibrils (1 mg mL^−1^) were placed on 300 mesh, Formvar layered copper grids. After 10 min incubation time, the grids were staining with 4% uranyl acetate solution for 2.5 min. After

three washing steps with dd H_2_O excess solvent was removed by filter paper. Measurements

were performed on a Jeol 1400 electron microscope with 120 kV acceleration voltage.

ThT‐fluorescence: 20 µL 50 µM of ThT solution in PBS was pipetted in a black 384 well‐plate and 4 µL of the nanofibril solution 1 mg mL^−1^ were added. For reference PBS with 10% DMSO (4 µL) instead of fibril solution was added. Subsequently, fluorescence emission was recorded λem = 488 nm upon excitation at λex = 440 nm with 10 nm bandwidth and multiple reads per well (3 × 3) using a Spark 20 M microplate reader (Tecan Group, Ltd). Data processing was performed with Origin software. A peptide fibril was considered as ThT‐active if the fluorescence intensity was at least twice as strong compared to the control (PBS containing 10% DMSO).

Proteostat fluorescence: 1 µL of a Proteostat stock solution (Enzo Life Science) were diluted in 100 µL PBS. 5 µL of the Proteostat solution was added to 10 µL of the peptide fibrils (1 mg mL^−1^) in a 384 well plate. Fluorescence intensity was determined at λem = 600 nm upon excitation at λex = 550 nm with 10 nm bandwidth and multiple reads per well (3 × 3) using a Spark 20 M microplate reader (Tecan Group, Ltd). Data processing was performed with Origin software.

Zeta‐potential: 60 µL of the peptides (1 mg mL^−1^) were diluted in 600 µL, 1 mM KCl solution and placed in a disposable folded capillary cuvette. All zeta‐potential measurements (Zetasizer, Malvern Instruments) were conducted in triplicates for each sample.

ATR‐FT‐IR: ATR‐IR spectra acquisition and the calculation of β−sheet content were conducted according to a previous report.^[^ ^20^ ^]^ Briefly, FT‐IR spectra of solid samples were recorded after lyophilization of fibril solutions using a Bruker Tensor II spectrometer equipped with a diamond crystal as ATR element with a spectral resolution of 2 cm–1, each spectrum was an average of 64 scans. The data was processed with Origin software. β−sheet amount was determined by calculating the integral of the peak at 1630 cm^−1^ (β‐sheet) relative to the integral between 1650–1700 cm^−1^ (native structure) from the 2^nd^ derivative of the FT‐IR spectra.

CD: Circular dichroism measurements were performed on a JASCO J‐1500 instrument in a 1 mm path length quartz cuvette (HellmaAnalytics) from 190 – 250 nm with peptides directly dissolved in MilliQ water to 1 mg mL^−1^ and subsequently pH adjusted to pH 7.4 with 0.1 M NaOH (aq.) or HCl (aq.) and incubated for 1 d to induce assembly. CD measurements were conducted in pure water to avoid aggregate formation which occurs in presence of phosphate ions from PB buffer and disturbs the measurement. It was previously shown that CD measurements in PB or MilliQ yield comparable results for other PA.^[^ ^91^ ^]^ The samples were incubated for 1 d and diluted to 0.1 mg mL^−1^ before measurement. Three accumulations were averaged for each measurement with a wavelength step size of 2 nm and scanning speed of 20 nm min^−1^. For each measurement, the corresponding background measurements were conducted and subtracted from the original spectrum.

Fluorescence microscopy: 1 µL Fibrils (1 mg mL^−1^) were stained with 9 µL, 50 µM ThT. 10 µL of the stained fibrils (0.1 mg mL^−1^) were placed on a microscopy glass. Fluorescence microscopy measurements were performed on a Leica DMi8 microscope with 10x air objective and equipped with a Leica MC170 HD camera with a filter setting λ_em_ = 527/30 nm upon excitation at λ_ex_ = 480/40 nm) and processed with the Leica LASX software.

### Cell culture

TZM‐bl and HeLa cells were cultured in Dulbecco´s Modified Eagle Medium (DMEM) supplemented with 10% (v/v) inactivated fetal calf serum (FCS), L‐glutamine (2 mM), penicillin (100 units mL^−1^) and streptomycin (100 µg mL^−1^). Jurkat cells were cultured in Roswell Park Memorial Institute (RPMI) 1640 Medium supplemented with 10% (v/v) fetal calf serum (FCS), L‐glutamine (2 mM), penicillin (100 units mL^−1^) and streptomycin (100 µg mL^−1^). CD4+ T cells were cultured in supplemented RPMI medium in presence of interleukin‐2 (IL‐2) or interleukin‐7 (IL‐7) and interleukin‐15 (IL‐15) as described below.

### Virus—Peptide Fibril—Cell Interaction

Infectious HIV‐1 stocks, GALV‐pseudotyped γ‐retroviral vector (GALV‐RV) were prepared analogous to a previous report and briefly described in the following sections.^[^ ^20^ ^]^

**Virus stocks**: For generating virus stocks 800000 HEK293T cells were seeded. Next day, transfection using different plasmids and TransIT‐LT1 Transfection Reagent (Mirus Bio, Cat: Mir 2305) according to manufacturer's protocol was performed. Two days after transfection virus stocks were harvested, centrifuged (3 min at 1300 rpm) and supernatants were stored at – 80°C. Infectious HIV‐1 stocks were prepared by transfection of HEK293T cells using pBRNL4.39‐92TH14 (5 µg), a plasmid containing a CCR5 tropic molecular HIV‐1 clone.^[^ ^92^ ^]^ GALV retroviral vector (GALV‐RV) was produced by cotransfection of HEK293T cells with following plasmids: GFP expressing Murine leukemia virus (MLV) vector E200 pcmE26‐gfp (2.3 µg), E848 pCsGPpA‐ed (1.9 µg) and glycoprotein derived from Gibbon ape Leukemia Virus (GALV, 0.77 µg).^[^ ^20^ ^]^

**HIV‐1 Infection Assay**: The effect of peptide fibrils on HIV‐1 infection was studied via a luminescence assay for β‐galactosidase, which was reported by TZM‐bl cells upon infection. 10000 TZM‐bl cells were seeded in 180 µl supplemented DMEM one day prior infection. The cells were inoculated with 20 µl PNF solution 1:1 (v/v) mixed with virus (1:500 dilution) after an incubation time of 10 min. The HIV‐1 infection assay was conducted in three technical replicates and repeated in three independent experiments unless stated otherwise. The assay was conducted as described before.^[^ ^20^ ^]^

γ**‐Retroviral Infection Assay of Jurkat cells**: 50000 Jurkat cells were seeded in 180 µl supplemented RPMI. The cells were inoculated with 20 µl PNF solution 1:1 (v/v) mixed with virus (1:3.2 dilution) after an incubation time of 10 min. The effect of peptide fibrils on retroviral transduction of Jurkat cells was analyzed 3 days post infection by determining the percentage of GFP + cells by flow cytometry. All assays were conducted as described before.^[^ ^20^ ^]^

γ**‐Retroviral transduction Assay CD4+ T cells**: Human CD4+ T cells were isolated from a Buffy coat using RosetteSep Human CD4+ Enrichment Cocktail (STEMCELL Technologies) according to manufacturer's protocol. Isolated T cells were activated using Dynabeads Human T‐Activator CD3/CD28 (GibcoTM) according to manufacturer's protocol in presence of 10 ng mL^−1^ IL‐2 (Miltenyi Biotec) for three days. After 3 days the magnetic Dynabeads were removed using a magnet and T cells were cultured in presence of 9 ng mL^−1^ IL‐7 (Miltenyi Biotec) and 10 ng mL^−1^ IL‐15 (Miltenyi Biotec). After 1 day 5 × 10^5^ T cells were transduced with GALV‐γ‐RV in absence and presence of PA1, PA2, EF‐C (0.26, 1.3 and 6.5 µM) and Vectofusin−1 (10 µg mL^−1^; Miltenyi Biotec), or RetroNectin (20 µg mL^−1^; Takara Bio). The concentration of the transduction enhancers corresponds to the concentration on cells and in the case for RetroNectin it corresponds to the concentration that was used to precoat the 24‐well plate. The 24‐well plate was coated with RetroNectin and incubated overnight at 4°C. Wells were blocked with PBS containing 2% BSA for 30 min and washed twice with PBS. Virus was centrifuged onto coated wells at 2,000 g at 32°C for 2 h. Viral supernatant was removed and CD4+ T cells were added and subsequently spin‐infected (300 g for 10 min) with virus again. 1 day after transduction the medium was exchanged. After 3 days GFP+ T cells were analyzed by flow cytometry. Viability was determined using LIVE/DEAD Fixable Aqua Dead Cell Stain Kit (ThermoFisher Scientific) according to manufacturer's protocol. For the investigation of the transduction enhancing effect and viability of peptides on CD4+ T cells the gating strategy shown in Figure S8A was applied.

**Cell Viability with TZM‐bl and Jurkat cells**: The cell viability after addition of peptides to TZM‐bl cells and Jurkat was studied via the CellTiter‐Glo assay. To this end, 20000 cells TZM‐bl or 50000 Jurkat cells were incubated with serial diluted peptides. After 3 days the supernatant was removed and 100 µL CellTiter‐Glo Reagent 1:1 diluted in PBS was added. After 10 min 50 µL was transferred to white microplate and luminescence was recorded by Orion microplate luminometer.

**Confocal laser scanning microscopy and degradation study with PA1‐3 and HeLa cells**: HeLa cells were seeded one day prior to conducting the assay (40000/well) in an 8‐well IBIDI slide. 4 µL of the preformed peptide fibrils (1 mg mL^−1^) were diluted with 4 µL Proteostat (Enzo Life Science, 1 µL stock in 999 µL PBS) and incubated for 10 min and further diluted with medium to receive a final peptide concentration of 20 µg mL^−1^. The nucleus of the HeLa cells was stained with Hoechst 33 342 (NucBlue, Thermo Fisher Scientific). The peptide solution was transferred to the HeLa cells and incubated for 30 min at 37°C before washing three times with PBS. The interaction of fibril clusters with cells was monitored after 30 min incubation time on a Stellaris 8 confocal laser scanning microscope (Leica) equipped with a 20x air objective and laser excitation wavelengths of 405 nm (Hoechst) and 561 nm (Proteostat). For the evaluation of aggregate degradation, the samples were further incubated at 37°C for 3 d and analyzed without any additional washing step. The evaluation of aggregate size and number was conducted by analyzing Proteostat stained aggregates via the 3D objects counter function of ImageJ 1.53f51 for 3 individual measurements (area 581.25 µm × 581.25 µm). The size distribution shows aggregates sizes larger than the threshold 10 µm^2^ in an area of 581.25 µm × 581.25 µm and repeated in three technical triplicates. The number of aggregates larger than 10 µm^2^ were counted via the 3D objects counter function of ImageJ 1.53f51. The mean number and standard deviations were determined by averaging at least three technical triplicates.

**Confocal laser scanning microscopy and degradation study with PA1‐3 and CD4+ T cells**: The degradation of the peptide assemblies on CD4+ T cells was determined by analyzing fluorescence microscopy images. To this end, 4 µL of the preassembled peptide fibrils EF‐C, PA1 and PA2 (1 mg mL^−1^, 1 d in PBS) were mixed with 4 µL of Proteostat (Enzo Life Science, 1 µL stock in 99 µL PBS) and incubated for 15 min. 2 µL of the stained fibrils were added to 198 µL preactivated and nucleus stained (Hoechst 33 342) CD4+ T cells (200 µL, 0.25 × 10^6^) resulting in a final 5 µg mL^−1^concentration of the peptide. After incubation for 30 min at 37°C the cells were washed with 3x with PBS and incubated in medium (supplemented RPMI containing IL‐15 and 7). Microscopy was conducted on a Zeiss LSM 710 confocal microscope with 20x and 63x objective and laser excitation wavelengths of 405 nm (Hoechst) and 561 nm (Proteostat). The measurements were performed after 30 min and 3 d of incubation at 37°C without any washing step in between. Control experiments to analyze changes of fibril aggregation were conducted analogously without the presence of cells. The evaluation of aggregate size and number was conducted by analyzing Proteostat stained aggregates via the 3D objects counter function of ImageJ 1.53f51 for 5 individual measurements (area 708.5 µm × 708.5 µm) of three individually repeated experiments.

**Confocal laser scanning microscopy and degradation study with PA library and HeLa cells**: Confocal laser scanning microscopy studies were performed for the visualization of the cell‐PA interaction. HeLa cells were seeded one day prior to conducting the assay (20000/well) in an 8‐well IBIDI slide. 2 µL of the preformed peptide fibrils (1 mg mL^−1^) were diluted with 2 µL Proteostat (Enzo Life Science, 1 µL stock in 99 µL PBS) and incubated for 10 min and further diluted with medium to receive a final peptide concentration of 10 µg mL^−1^. The nucleus of the HeLa cells was stained with Hoechst 33 342 (NucBlue, Thermo Fisher Scientific). The peptide solution mixture was transferred to the HeLa cells and incubated for 30 min at 37°C before washing three times with PBS. The interaction of fibril clusters with cells was monitored after 30 min incubation time on a Stellaris 8 confocal laser scanning microscope (Leica) equipped with a 20x air objective and laser excitation wavelengths of 405 nm (Hoechst), and 561 nm (Proteostat). For the evaluation of aggregate degradation, the samples were further incubated at 37°C for 3 d and analyzed without any additional washing step. The evaluation of aggregate size and number was conducted by analyzing Proteostat stained aggregates via the 3D objects counter function of ImageJ 1.53f51 for 3 individual measurements (area 1008 µm × 1008 µm). The size distribution shows aggregates sizes larger than the threshold 10 µm^2^ in an area of 1008 µm × 1008 µm. The number of aggregates were determined by counting aggregates larger than the threshold 10 µm^2^ in an area of 1008 µm × 1008 µm and repeated in three technical triplicates.

**Control experiments degradation analysis**: To check changes of fibril aggregation without cells fluorescence microscopy measurements (Leica DMi8 microscope, 10x air objective, Leica MC170 HD camera, λem = 527/30 nm, λex = 480/40 nm) were performed analogously to the cell‐experiments with PA1‐3. Briefly, 4 µL of the preformed peptide fibrils (1 mg mL^−1^) were diluted with 4 µL Proteostat (Enzo Life Science, 1 µL stock in 999 µL PBS) and incubated for 10 min and further diluted with medium to receive a final peptide concentration of 20 µg mL^−1^. The fibril aggregates were monitored after 30 min incubation at 37°C and for the evaluation of aggregate degradation, the samples were further incubated at 37°C for 3 d and analyzed without any additional washing step. The evaluation of aggregate size and number was conducted by analyzing Proteostat stained aggregates via the 3D objects counter function of ImageJ 1.53f51 for 3 individual measurements (area 1331.2 µm × 1331.2 µm). The evaluation of aggregate size and number was conducted by analyzing Proteostat stained aggregates via the 3D objects counter function of ImageJ 1.53f51 for at least three individual measurements (area 1331.2 µm × 1331.2 µm) of three individually repeated experiments.

**Statistics**: Statistical analysis were performed using GraphPad Prism version 9.5.1. P‐values for statistical significance were determined by two‐way ANOVA with Dunnett's multiple comparison test and are indicated with * p ≤ 0.05, ** p ≤ 0.01, ** p ≤ 0.001, NS not significant.

## Conflict of Interest

J.M. and L.R. are inventors of granted and filed patents applying peptide nanofibrils as promoter of retroviral gene transfer. T.W., J.M., C.V.S., J.H., L.R. and K.K., filed a patent application for the usage of peptide amphiphiles as degradable enhancers.

## Author Contributions

K.Kaygisiz conceived the idea, conceptualized, designed, and carried out the experiments, analyzed data, performed the formal analysis, and wrote the original draft. L.R.W. designed, and carried out the experiments related to virus transduction and cell‐viability, analyzed data, performed the formal analysis, and revised the manuscript. A.I. performed molecular dynamics simulation, analyzed data and revised the manuscript. J.H. analyzed data and carried out experiments related to peptide synthesis. K.Kremer, J.M., C.V.S and T.W conceptualized and supervised, provided the funding resources, and revised the manuscript. The final manuscript was approved by all authors.

## Supporting information

Supporting Information

## Data Availability

The data that support the findings of this study are available from the corresponding author upon reasonable request.
