# Supplementary material for: Peptide Amphiphiles as Biodegradable Adjuvants for Efficient Retroviral Gene Delivery
Source: Adv Healthc Mater. 2023 Nov 23;13(4):2301364. doi: 10.1002/adhm.202301364 (PMC11468294; doi:10.1002/adhm.202301364)
Supplement: Supplementary file 1 — Supporting Information [file ADHM-13-2301364-s001.pdf]

ADVANCED HEALTHCARE MATERIALS

Supporting Information

for *Adv. Healthcare Mater.*, DOI 10.1002/adhm.202301364

Peptide Amphiphiles as Biodegradable Adjuvants for Efficient Retroviral Gene Delivery

Kübra Kaygisiz, Lena Rauch-Wirth, Aysenur Iscen, Jan Hartenfels, Kurt Kremer, Jan Münch, Christopher V. Synatschke* and Tanja Weil**

Supporting Information for

Peptide amphiphiles as biodegradable adjuvants for efficient retroviral gene delivery

Kübra Kaygisiz[1], Lena Rauch-Wirth[2], Aysenur Iscen[3], Jan Hartenfels[1], Kurt Kremer[3], Jan Münch[2]*, Christopher V. Synatschke[1]*, Tanja Weil[1]*

1 Department Synthesis of Macromolecules, Max Planck Institute for Polymer Research, Ackermannweg 10, 55128 Mainz, Germany

2 Institute of Molecular Virology, Ulm University Medical Center, Meyerhofstraße 1, 89081 Ulm, Germany

3 Polymer Theory Department, Max Planck Institute for Polymer Research, Ackermannweg 10, 55128 Mainz, Germany

Correspondence to be addressed to: Jan.Muench@uni-ulm.de, synatschke@mpip-mainz.mpg.de; weil@mpip-mainz.mpg.de

Content

1 Purity and Characterization of PA1-3 and P1-3 3
2 Aggregation of PA1-3 and P2 fibrils in ultrapure water and phosphate buffer 4
3 ATR FT-IR analysis of PA1-PA3, P1-P3 5
4 ThT fluorescence of PA1-3 and P1-3 7
5 Cell-PA interaction 8
6 D-Amino acids substitution of PA1 13
7 Effects of PA modifications on properties and activity 14
7.1 Effects of hydrophobic moiety exchange on properties and activity 15
7.2 Effects of linker exchange on properties and activity 19
7.3 Effects of sequence exchange on properties and activity 23
8 Cell-Viability of PA library 31
9 Proteostat Assay of PA library 32
10 TEM of PA library 33
11 ATR-IR of PA library 35
12 Chemical structures of PA library 37
13 Computational Methods 41
14 References 47

1 Purity and Characterization of PA1-3 and P1-3

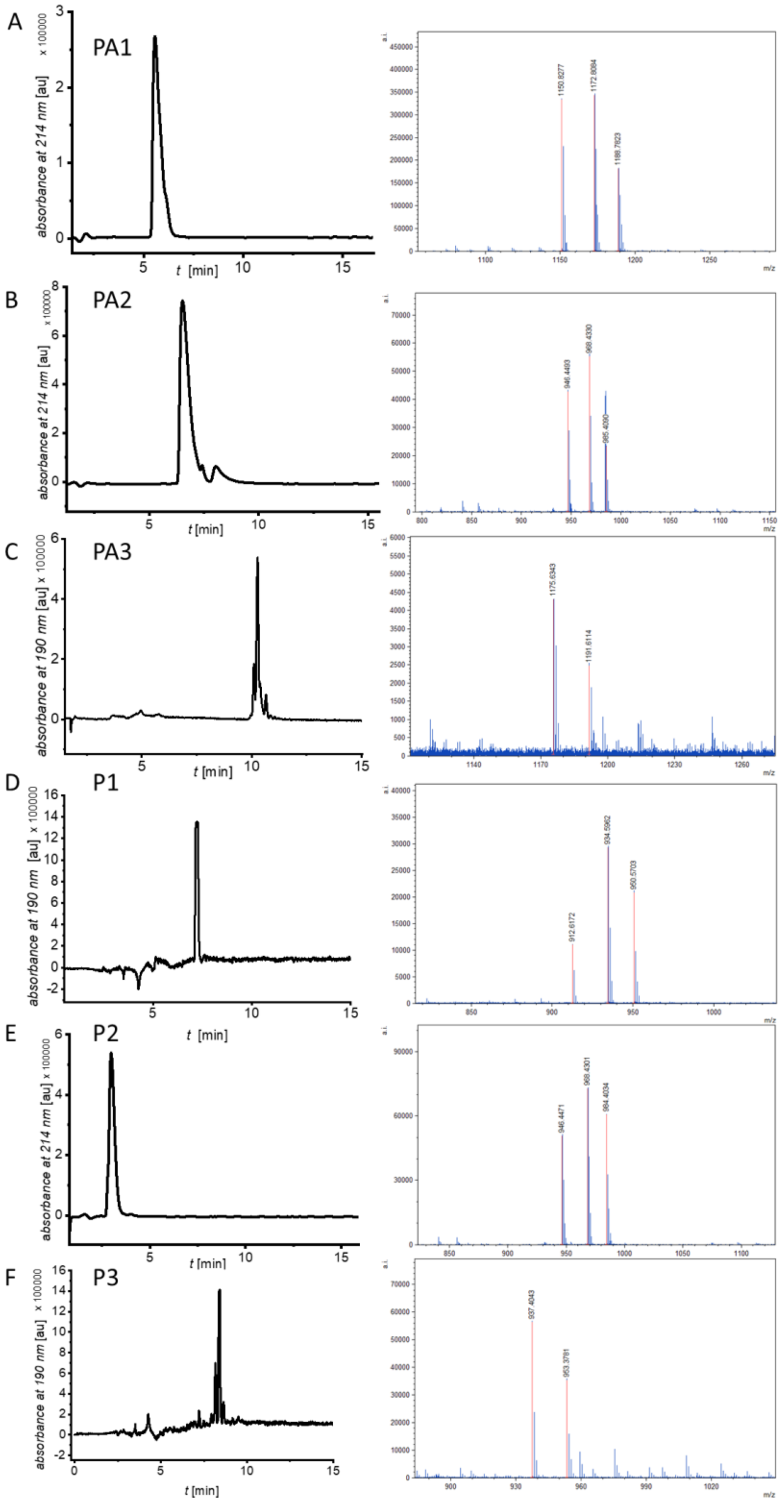

Figure S1 Mass determination and characterization of PA1-3 and P1-3. High performance liquid chromatography traces (left) and mass spectrometry (right). **A** PA1, M = $C_{58}H_{111}N_{13}O_{10}$, LC-MS (m/z) calc. for $[M+H]^+$ 1150.87; found: 1150.95, purity >95%. MALDI-ToF MS (m/z) calc. for $[M+H]^+$, 1150.8650; found: 1150.8277. **B** PA2, M = $C_{63}H_{97}N_{11}O_9S$, LC-MS (m/z) calc. for $[M+H]^+$ 1184.73; found: 1184.62, purity >90%. MALDI-ToF MS (m/z) calc. for $[M+H]^+$, 1184.7264; found: 1184.6454. **C** PA3, M = $C_{58}H_{111}N_{13}O_{10}$, HPLC purity >90%. MALDI-ToF MS (m/z) calc. for $[M+Na]^+$, 1150.6903; found: 1175.6343. **D** P1, M = $C_{42}H_{81}N_{13}O_9$, HPLC purity >95% and MALDI-ToF MS (m/z) calc. for $[M+H]^+$, 912.6353; found: 912.6172. **E** P2, M = $C_{47}H_{67}N_{11}O_8S$, LC-MS (m/z) calc. for $[M+H]^+$ 946.50; found: 946.59, purity >95%. MALDI-ToF MS (m/z) calc. for $[M+H]^+$, 946.4973; found: 946.4471. **F** P3, M = $C_{39}H_{66}N_{10}O_{15}$, HPLC purity >70%. MALDI-ToF MS (m/z) calc. for $[M+Na]^+$, 937.4607; found: 937.4043.

2 Aggregation of PA1-3 and P2 fibrils in ultrapure water and phosphate buffer

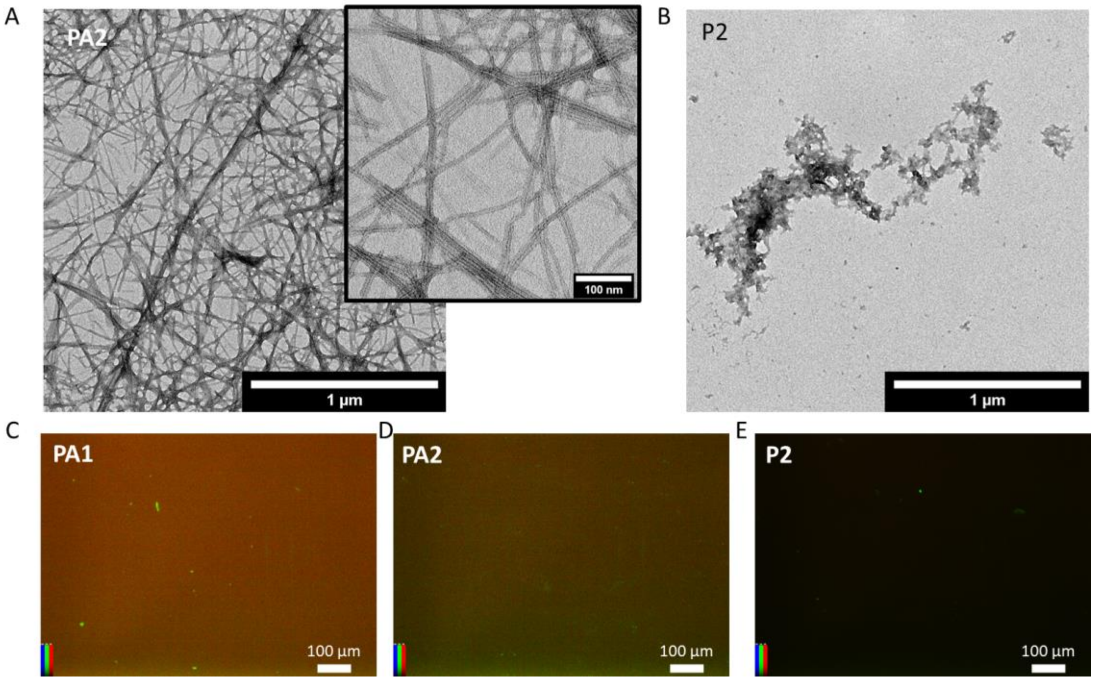

Figure S2 Characterization of PA2 and P2 incubated 1 d in pure MilliQ water. **A** TEM image of PA2, scale bar 1 µm, detail 100 nm. **B** TEM image of P2, scale bar 1 µm. **C, D, E** Fluorescence microscopy images of PA1, PA2 and P2, respectively. Samples were diluted from 1 mg mL^{-1} to 0.1 mg mL^{-1} with ThT-solution in MilliQ, final concentration 45 µM, contrast setting 0-150 for all images, scale bar 100 µm.

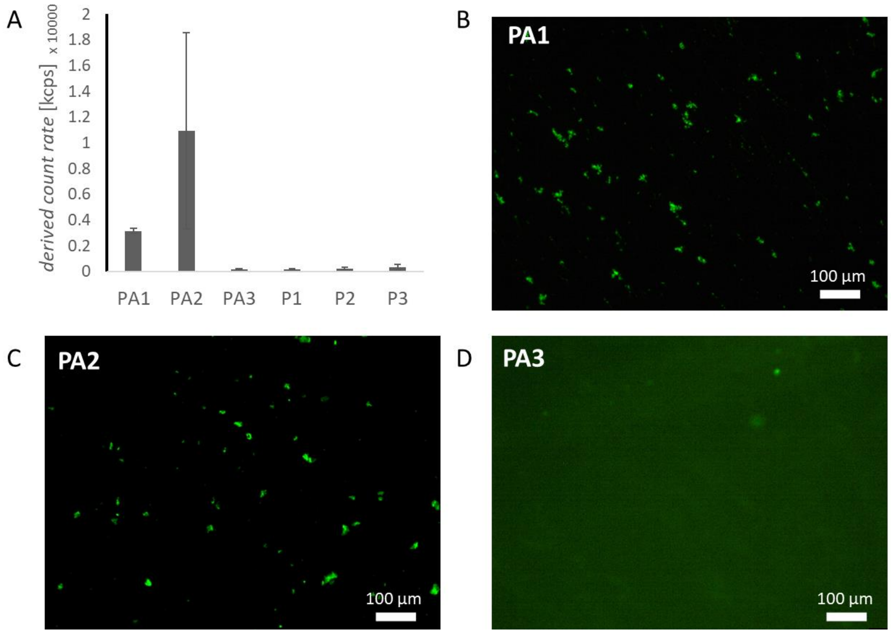

Figure S3 Formation of µm-sized aggregates of PA1 and PA2 in PBS. **A** Scattering intensity of light (633 nm) during zeta-potential measurements for PA1-3 and P1-3. **B, C, D** Fluorescence microscopy images of PA1, PA2 and PA3, respectively. Samples were diluted from 1 mg mL^{-1} to 0.1 mg mL^{-1} with ThT-solution in PBS, final concentration 45 µM, scale bar 100 µm.

3 ATR FT-IR analysis of PA1-PA3, P1-P3

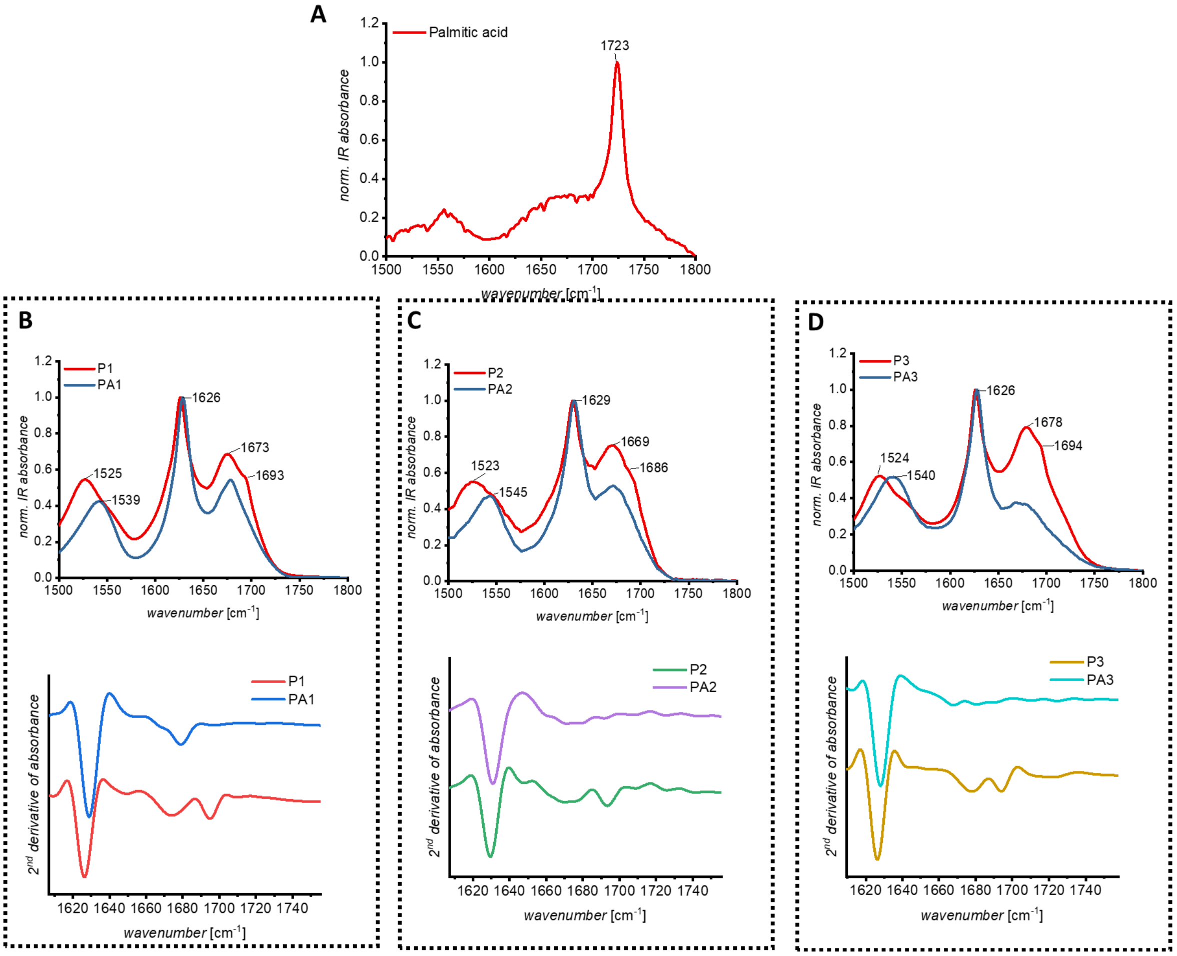

Figure S4 ATR-IR measurements. **A** ATR-IR spectrum of palmitic acid. **B-D Top** ATR-IR spectra of the amide I and II regions of PA1–PA3 and P1–P3 (1 mg mL^{-1} in PBS lyophilized). Chemically similar peptides are displayed in one diagram: PA1 and P1 (**B**), PA2 and P2 (**C**), PA3 and P3 (**D**). **B-D Bottom** 2nd derivation of IR absorbance of PA1 (C_{16}-VVVAAAKKK-NH_2) and P1 (VVVAAAKKK-NH_2) **(B), ** 2nd derivation of IR absorbance of PA2 (C_{16}-FQFKFKC -NH_2) (**C**), P2 (FQFKFKC-NH_2) and 2nd derivation of IR absorbance of PA3 (C_{16}-VVVAAAEEE-NH_2) and P3 (VVVAAAEEE-NH_2) (**D**).

All peptides P1–P3 and PA1–PA3 show a main peak around 1630 cm^{-1} which is assigned to parallel β-sheets (**Figure S4B-D**). Another intense, broad peak in the amide I region was found at approx. 1660-80 cm^{-1} indicating β-turn structural elements.[1,2] A small peak at 1694 cm^{-1} can be assigned to antiparallel β-sheets and was found only for P1–P3, but not in the PAs.[3,4] The amide II peak maximum for P1–P3 is located at approx. 1520 cm^{-1}, whereas for PA1–PA3, it occurs at approx. 1540 cm^{-1}. The changes in the amide I and amide II region indicate the transformation of amorphous aggregates (P1, P3) to fibrillar assemblies (PA1, PA3) in accordance with TEM measurements.[5] The decrease of the peak at 1694 cm^{-1} and the shift of the amide II band to higher wavenumbers for PA1–PA3 can be assigned to an increase of parallel β-sheet structures and decrease of antiparallel β-sheet. Similar peak shifts are reported for observations regarding the transition of amorphous aggregates to fibrillar assemblies of Aβ(1–40)[6–8] and lysozyme[9] for time-dependent assembly processes. Further, amorphous aggregates show less β-sheet content compared to fibrillar assemblies.[6,10] The relative amount of β-sheet secondary structural elements was quantified by integration of the IR absorption bands in the amide I region.[11] Since amino acid side chains can also contribute to the IR absorption in the amide I region, each peptide was only compared to its fatty acid conjugate. The palmitic acid

residue does not contribute to the IR absorbance in the amide I region (**Figure S4**). A quantitative analysis of the amide I region was executed by integration of the 2nd derivative peak area of the β-sheet (1630 cm^{-1}) and non-ordered or other structural elements (1640–1690 cm^{-1}, **Figure S4**).[11,12]

4 ThT fluorescence of PA1-3 and P1-3

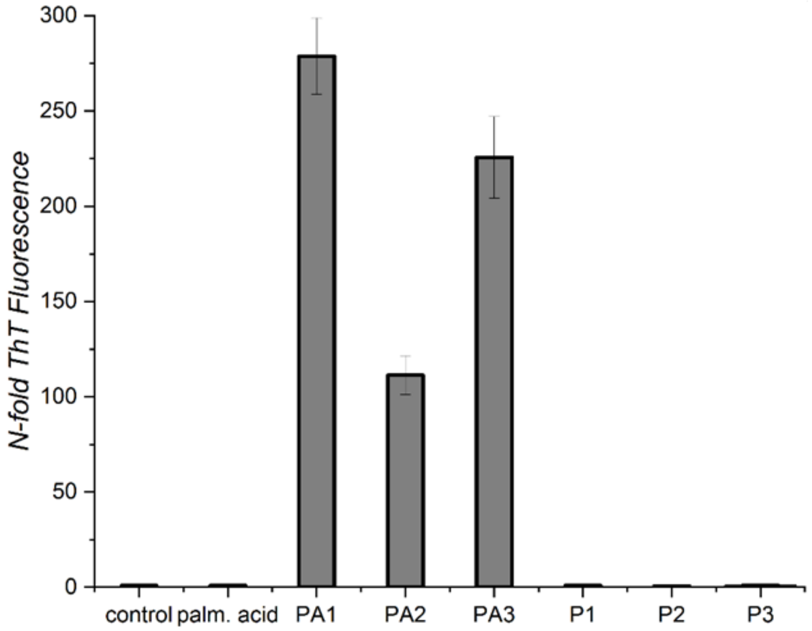

Figure S5 N-fold Thioflavin-T fluorescence intensity at 488 nm excitation for palmitic acid, PA1-3 and P1-3 relative to PBS (control).

5 Cell-PA interaction

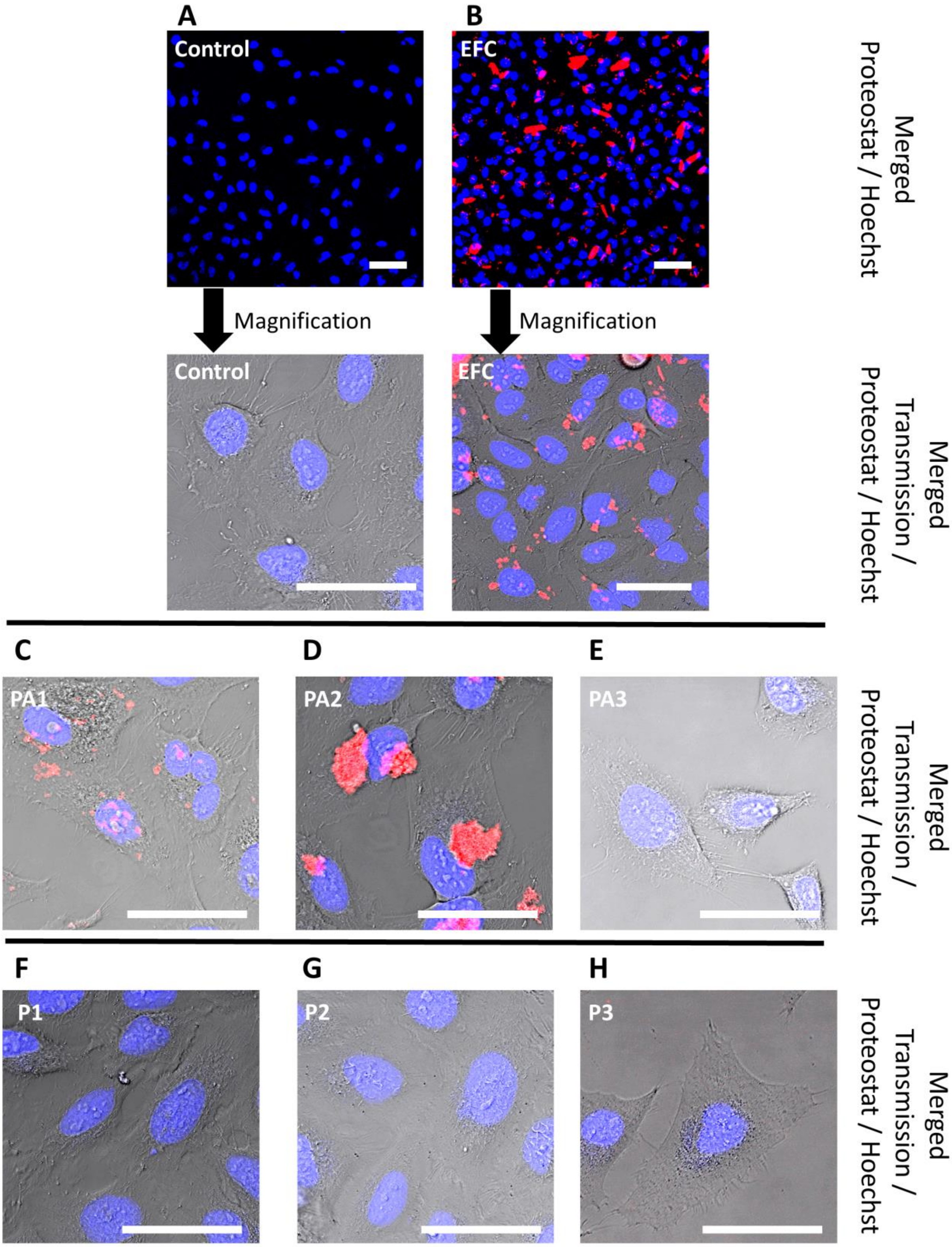

Figure S6 Laser scanning microscopy micrographs of **A** cells only (control), and Proteostat-stained (red) peptides **B** EF-C (QCKIKQIINMWQ), **C** PA1, **D** PA2, **E** PA3, **F** P1, **G** P2, **H** P3 added to HeLa cells (nucleus in blue). The peptides (20 µg mL^{-1}) were added to cells for 30 min, washed and analyzed. EF-C, PA1, PA2 revealed association with cellular membranes while PA3, P1-3 does not associate with cells, scale bar 50 µm.

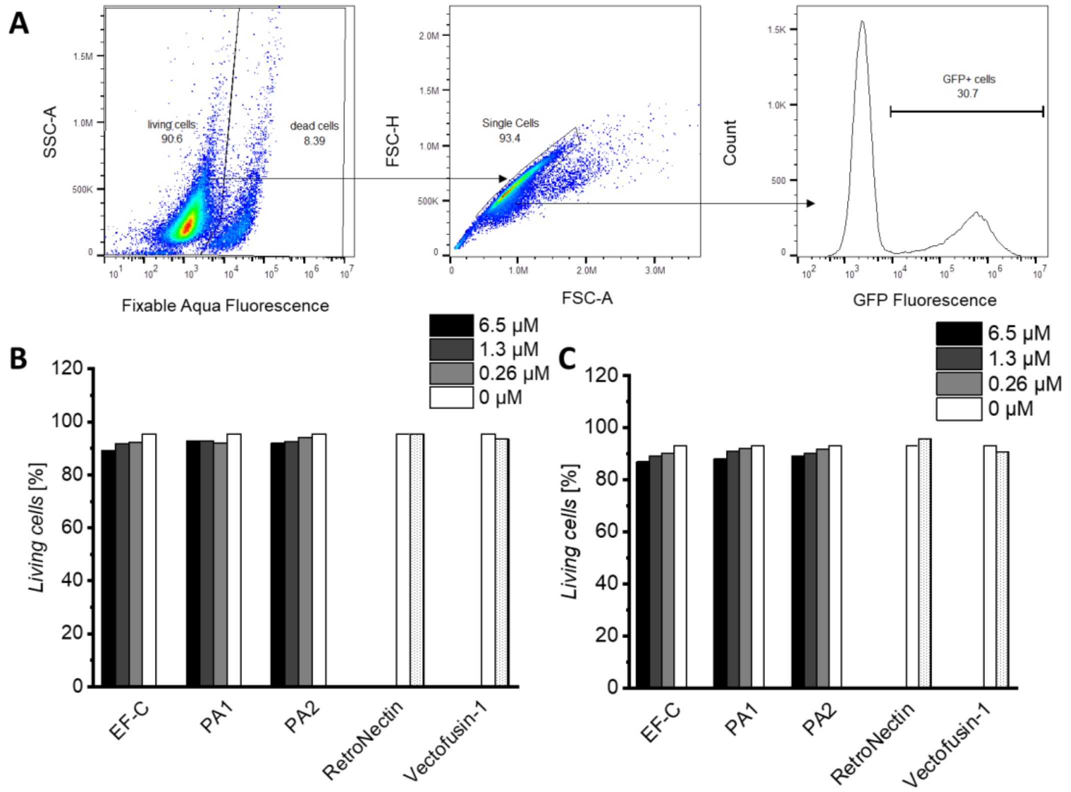

Figure S7 Viability of CD4+ T cells. **A** Gating strategy for analysis of living GFP+ cells. Cells were stained with LIVE/DEAD™ Fixable Aqua Dead Cell Stain Kit. Dead cells were initially eliminated by gating on viability dye-negative cells (SSC-A vs. Fixable Aqua Fluorescence) and afterwards for singlets (FSC-H vs. FSC-A). GFP+ cells were measured using the FITC channel. **B** Viability of CD4+ T cells after transduction with GALV-RV and treatment with different concentrations of transduction enhancing peptides EF-C, PA1 and PA2 (0, 0.26, 1.3, 6.5 µM) and 10 µg mL^{-1} (Vectofusin-1), 20 µg mL^{-1} (RetroNectin) after 3 days. **C** Control experiments with non-transduced CD4+ T cells to determine viability after treatment with transduction enhancers for 3 days.

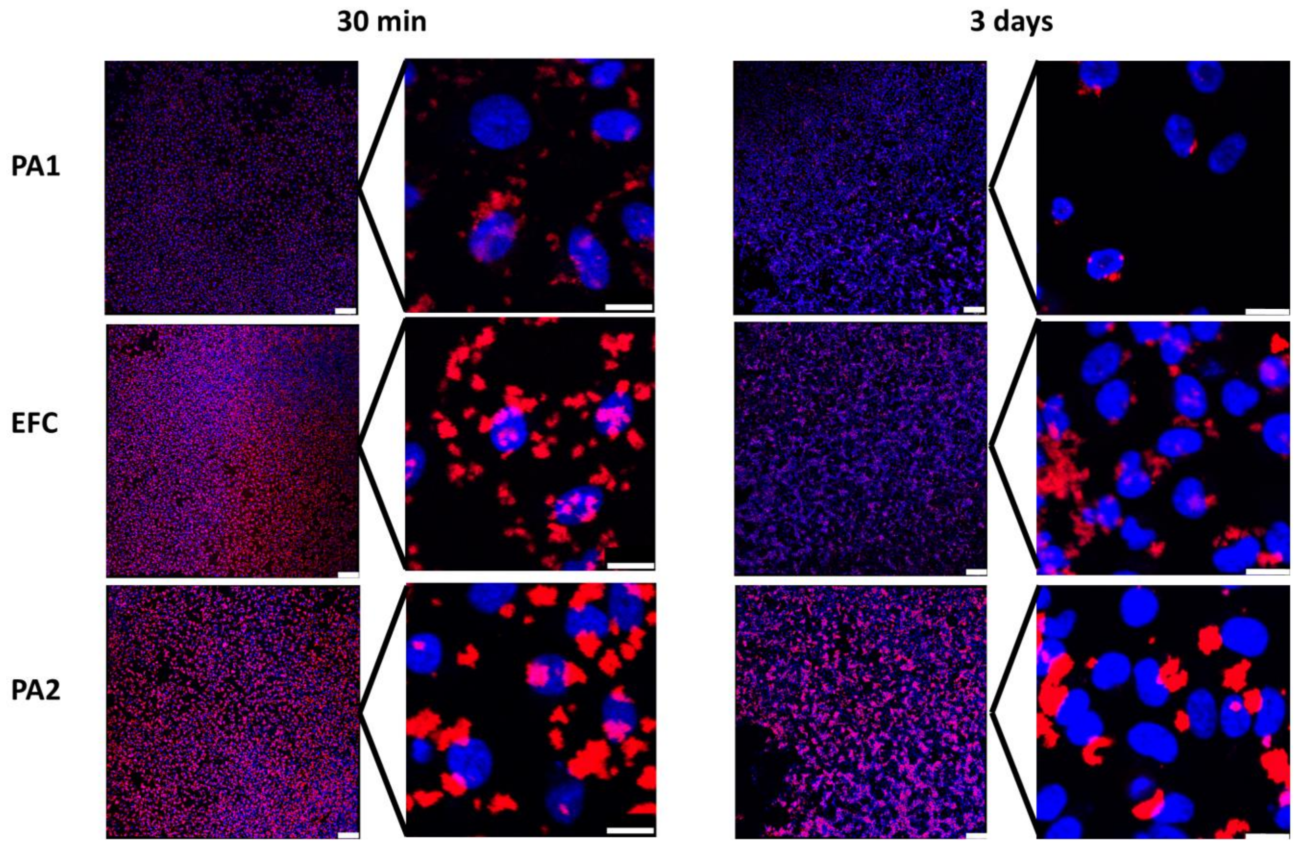

Figure S8 Degradability of µm-sized fibrillar aggregates formed by PA1 and PA2 after 30 min and 3 d in cell culture shown via laser scanning microscopy images of Proteostat-labeled EF-C, PA1 and PA2 (red) incubated with HeLa cells (nucleus in blue). In general, PA1 reveals smaller µm-sized aggregates than PA2. After 3 days in cell culture, almost all small aggregates of PA1 are degraded, whereas aggregates formed by PA2 stay almost unchanged, scale bar 200 µm for overview (left) and 20 µm for detail (right).

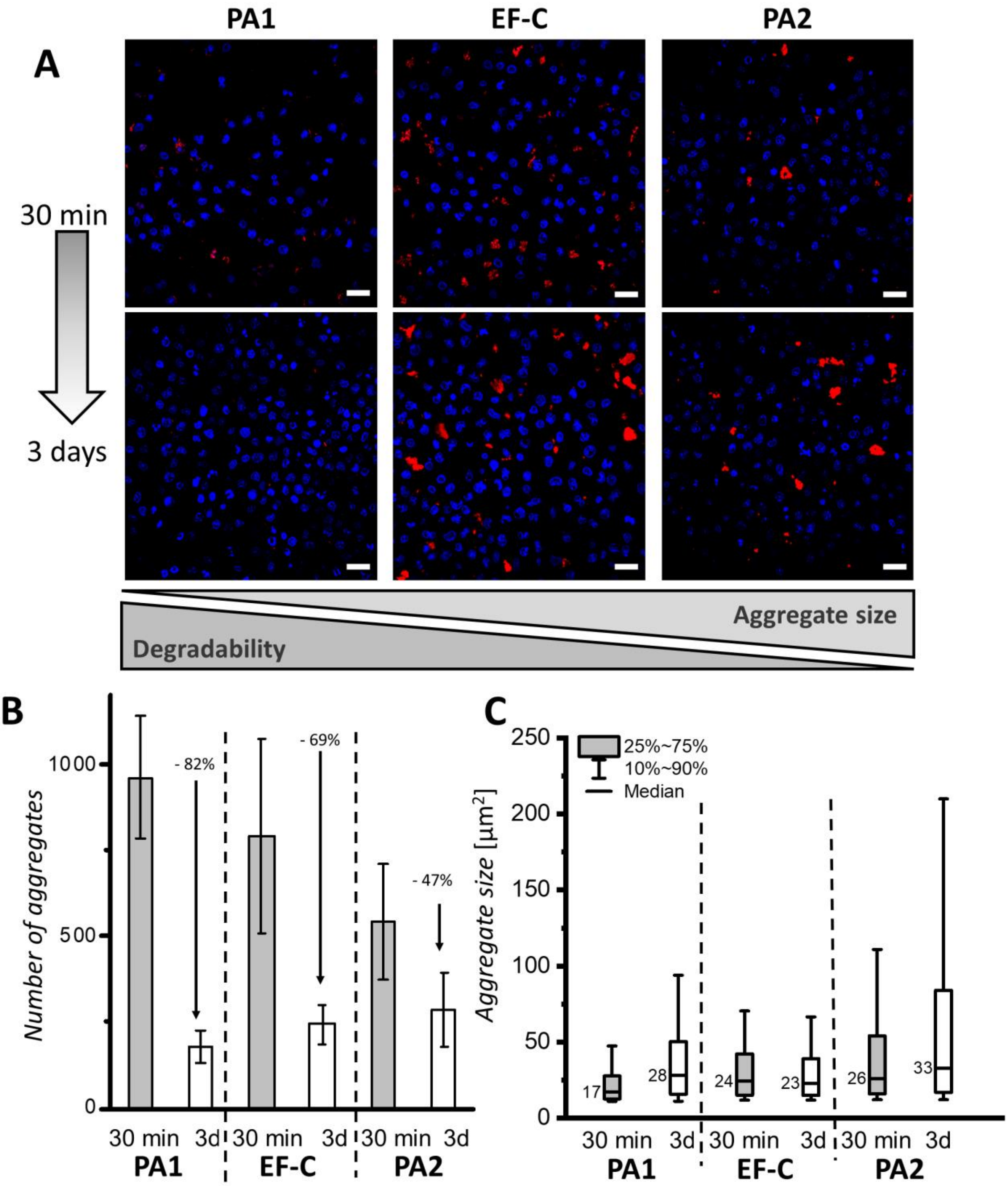

Figure S9 Degradability of µm-sized fibrillar aggregates formed by EF-C, PA1 and PA2 after 30 min and 3 d in CD4+ T cell culture. **A** Representative laser scanning microscopy images of Proteostat-labeled EF-C, PA1 and PA2 (red) incubated with CD4+ T cells (nucleus in blue). In general, PA1 reveals smaller µm-sized aggregates than PA2 and EF-C. After 3 days, almost all small aggregates of PA1 are degraded, scale bar 20 µm. **B** Bar plot showing number of aggregates > 10 µm^2 in an area of 708.5 µm × 708.5 µm in cell culture after 30 min and 3 d incubation (1 µg peptide added to 250,000 cells), error bars indicate standard deviations from five technical replicates. **C** Box plot showing size distribution and in an area of 3543 µm × 3543 µm in cell culture after 30 min and 3 d incubation (1 µg peptide added to 250,000 cells).

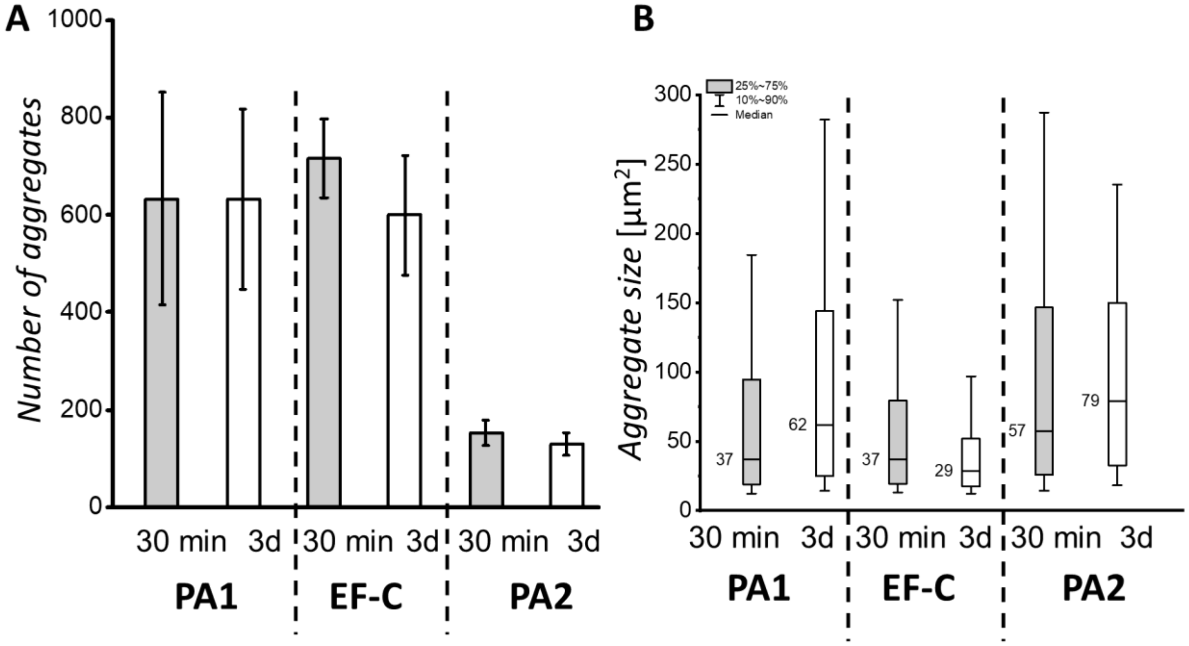

Figure S10 Stability test of Proteostat stained fibrils without the presence of cells. **A** Bar plot showing number of aggregates > 10 µm^2 in an area of 1331.2 µm × 1331.2 µm at 0.1 mg mL^{-1} incubated at 37°C, 250 rpm in PBS after 30 min and 3 d incubation, error bars indicate standard deviations from five technical replicates. **B** Box plot show size of peptide fibrils > 10µm^2 in 3991.65 µm × 3991.65 µm at 0.1 mg mL^{-1} incubated at 37°C, 250 rpm in PBS. Aggregate analysis was conducted via fluorescence microscopy by cumulating triplicate measurements. There is no significant change in aggregate size and number over the course of 3 days. Under these conditions PA2 forms less aggregates, which are larger in size compared to PA1 and EF-C.

6 D-Amino acids substitution of PA1

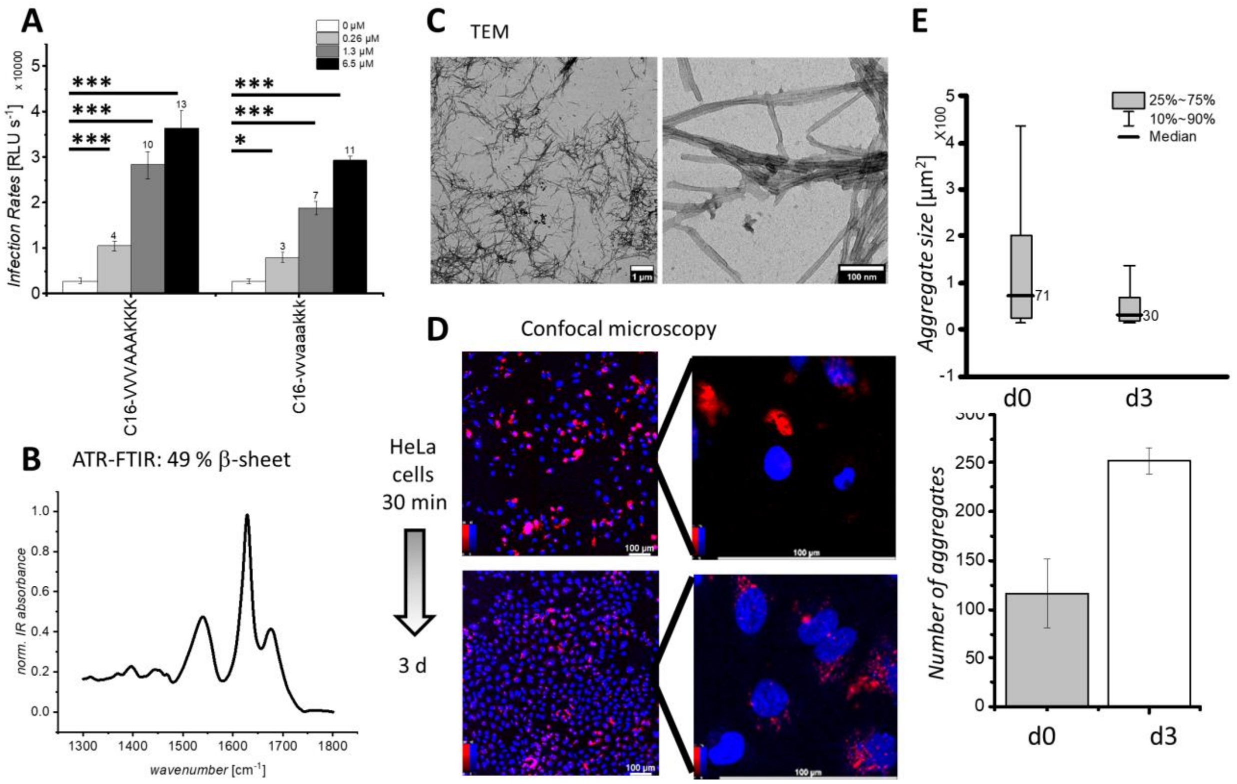

Figure S11 A Infectivity assay showing HIV-1 infection rates of TZM-bl cells observed in the presence of increasing concentrations of PA1 and pa1, the D-amino acid pendant of PA1 (6.5 µM, 1.3 µM, 0.26 µM). The baseline (0 µM) shows infection rate without the addition of peptides. Values above each bar represent the n-fold enhancement compared to 0 µM. Mean values and standard deviations are derived from three repetitions of triplicate infections. P-values for statistical significance were determined by two-way ANOVA test and are indicated with * $p \leq 0.05$, ** $p \leq 0.01$, *** $p \leq 0.001$, NS not significant. RLU s^{-1} are relative light units per second. **B** The β-sheet content of pa1 is 49% and is calculated from the FT-IR spectra. **C** pa1 assembling into fibrils as determined by TEM, scale bars 1 µm (left) and 100 nm (right). **D** Representative confocal laser scanning microscopy images of Proteostat-labeled pa1 (red) incubated with HeLa cells (nucleus in blue), scale bar 100 µm. **E** Bar plot showing number of aggregates > 10 μm^2 and box plot showing size distribution in an area of 1008 µm × 1008 µm in cell culture after 30 min and 3 d incubation (2 µg peptide added to 20,000 cells), deviations indicate three technical replicates. In general, pa1 reveals larger µm-sized aggregates than PA1. After 3 days in cell-culture, aggregates of pa1 are incompletely degraded into smaller aggregates (>10 μm^2), which causes an increase of number of aggregates.

C_{16}-vvvaaakkk-NH_2 (pa1) is analogous to PA1 but contains only D-amino acids, which cannot be degraded from cells (**Figure S11**). As expected C_{16}-vvvaaakkk-NH_2 is not degraded and forms large aggregates (71 μm^2) which are further decreasing in size (71 μm^2 at d0 → 30 μm^2 at d3) but not in number (increase in 116%), which indicates that aggregates are not degraded but rather breaking up to smaller parts.

7 Effects of PA modifications on properties and activity

To investigate the effects of modification in the PA sequence, we created various peptide amphiphiles derived from PA1 by varying the (I) hydrophobic moiety, (II) the linkage chemistry and (III) the peptide sequence (**Figure S12, SI chapter 7.1 – 7.3**). The color code for sequence representation is yellow for the hydrophobic moiety, blue for cationic amino acids, red for non-polar amino acids, purple for polar uncharged amino acids, and green for anionic amino acids. To be concise, only the summarized information will be discussed, all original data for the peptides are summarized in the (**Figure S26 - Figure S32**). The tested peptides were biocompatible and not cytotoxic at the tested concentrations on TZM-bl cells (**Figure S25**).

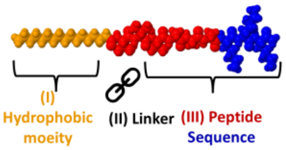

Original sequence		N-fold infectivity at 1.3 µM	Degradability / %
PA1	C_{16} VVVAAAKKK-NH_2	10±3	94%

(I)

Hydrophobic moeity	Replace C16	N-fold infectivity at 1.3 µM	Degradability / %
Fatty acid length	C12	6±1	-32%
	C9	2±0	78%
Aromatic residues	Fmoc	5±1	28%
	Nap	1±1	ND
Unsaturated fatty acids	Eicosapentaene acid	12±1	99%
	Alphalinoleinic acid	1±0	ND

(II)

Linker	Replace **Amide**:	N-fold infectivity at 1.3 µM	Degradability / %
Functional group	Ester	17±5	31%
	Thioester	11±4	87%
	Disulfide	4±2	ND
Enzyme responsive sequence	GFLG (Cathespin B)	11±4	4%
	GPLGV (MMP)	8±3	28%

(III)

Peptide sequence	Replace VVVAAAKKK-NH_2	N-fold infectivity at 1.3 µM	Degradability /%
PA1 inspired	VVVAAAEEE-NH_2	1±0	ND
	AVAKKK-NH_2	2±0	94%
	VVAAHHH-NH_2	2±0	26%
	GGGSSKKK-NH_2	2±1	ND
	AGAGRRR-NH_2	1±0	ND
	AAVVHHK-NH_2	6±3	-123%
	GGVVRR-NH_2	1±1	ND
β-sheet prone sequences	FQFKFKC-NH_2	9±1	53%
	IIQKIK-NH_2	4±1	28%
	AQAKAK-NH_2	4±1	-83%
	LLLKK-NH_2	3±0	27%
	KGVPGVGK-NH_2	1±0	ND
Endogeneous sequences	AGGRVK-NH_2	3±1	ND
	LNGGVKK-NH_2	6±1	48%
	ALAAGKK-NH_2	5±1	87%
	WEALKK-NH_2	1±0	ND
	RRWQWR-NH_2	0±0	ND
	KYIHQNYTKALAGKLV-NH_2	5±2	-110%
	NFYLQVNKINK-NH_2	5±1	-123%
	RYYASLRHYLNLVTRQRY-NH_2	5±1	43%
	GKVGSK -NH_2	2±1	ND
	AKAVGK-NH_2	6±3	89%
	KIISFK-NH_2	2±1	-84%

Figure S12 Summarized overview of peptide amphiphile library in which systematic variations the original sequence PA1 (C_{16}-VVVAAAKKK-NH_2) were conducted to study the effects of the (I) hydrophobic moiety, (II) the linkage chemistry and (III) the peptide sequence on infectivity enhancement and degradability (see **SI chapter 7.1 – 7.3**).

In the following section a compound is considered infectivity enhancing if n-fold infectivity at 1.3 µM is larger than 2. A compound is considered to form µm-sized aggregates if visible particles were observed in widefield microscopy (brightfield or ThT-staining filter, objective 10x) or confocal microscopy (stained with proteostat, objective 20 x) and samples showed a derived count rate larger than 1000 kcps in Zeta-Potential measurements. A compound is considered ThT active if a ThT-fluorescence larger than 2 relative to a control samples (PBS with 10% DMSO) is observed.

For the degradability only the PAs which could be stained with Proteostat (**Figure S26**) and were visible in microscopy (**Figure S15**, **Figure S18**, **Figure S21**, and **Figure S22**) were studied in detail. To determine

whether a PA is degraded or not, Proteostat stained PAs which are added to Hela cells (2 µg peptide added to 20.000 cells) and studied via confocal laser scanning microscopy. Microscopy is conducted at day 0 (d0, after 30 min of incubation) and at day 3 (d3, after 72 h of incubation). The number of aggregates >10 μm^2 is counted via 3D object counter in ImageJ in an area of 1008 µm × 1008 µm.

A PA is considered as degradable if the number and the size of aggregates are reduced after 3 days. The efficiency is determined by the percentual degradation (aggregate number at d3 / aggregate number at d0). Three technical replicates were evaluated for each sample. A large deviation in number and size of aggregates represents a nonhomogeneous distribution and polymorphism of aggregates.

In the following, we will distinguish between three aggregation types for fibrillar structures: Fibrils which were not aggregating (below resolution limit of the microscope, approx. 650 nm), moderately aggregating (< 50 μm^2) and aggregating to large particles (> 50 μm^2).

7.1 Effects of hydrophobic moiety exchange on properties and activity

To investigate the effects of varying hydrophobic moieties on assembly, aggregation and activity, we attached different lengths of fatty acids, various unsaturated fatty acids and different size of aromatic groups to the sequence VVVAAAKKK-NH_2 (**Figure S29**).

Combining the VVVAAAKKK-NH_2 peptide sequence with different hydrophobic residues can maintain infectivity enhancement, if the resulting structures are able to form fibrils, aggregate into µm-sized particles and show a positive zeta-potential (**Figure S13**). The hydrophobic residues naphthalene (Nap), pelargonic acid (C_9) and alpha linolenic acid are not able to maintain activity because they either don't form fibrils (alpha linolenic acid) or do not aggregate (Nap, C_9, alpha linolenic acid) or show almost neutral zeta-potential (nap, alpha linolenic acid). We find a minimum alkyl chain length of lauric acid (C_{12}) necessary to observe efficient infectivity enhancement. However, modifications with C_{12} result in large aggregate formation, which even increases the number of aggregates after 3 days, because the aggregates are partially degraded – most likely due to their initial size. C_9 modified PA is reduced in number of aggregates, but increases in size after 3 days in presence of HeLa cells, which indicates that beside degradation also aggregation into larger clusters takes place.

Combining VVVAAAKKK-NH_2 with long, polyunsaturated fatty acid residues like eicosapentaenoic acids increases infectivity enhancement strongly. Eicosapentaenoic acid-VVVAAAKKK-NH_2 forms spherical structures composed of fibrillar structures (**Figure S27**), which aggregate into a high number of equally distributed small aggregates with average size of 6-7 μm^2. The infectivity enhancement of eicosapentaenoic acid-VVVAAAKKK-NH_2 is similar to EF-C or PA1, which makes it a clinically interesting degradable PA. Interestingly, alpha linolenic acid is a fatty acid, which has 2 less unsaturated sites than eicosapentaenoic acid, and is neither forming fibrils, nor it is aggregating or active (**Figure S13**). In contrast the active PA Fmoc-VVVAAAKKK is forming fibrils leading to very large µm-sized aggregates, which cannot be degraded by cells (**Figure S14**).

We therefore conclude that the capacity to form fibrils, which can aggregate can be traced back to the flexibility of the hydrophobic moiety, *i.e.* the increased flexibility for the less unsaturated alpha linolenic acid is limiting self-assembly and aggregation.

A

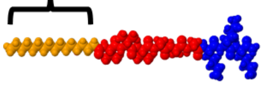

Sequence	Infectivity enhancing n-fold at 1.3 µM	Fibril Formation	Degradability / % (rel. to initial particles > 10 µm²)
C_{16}-VVVAAAKKK-NH_2	10±3	Yes	94%
C_{12}-VVVAAAKKK-NH_2	6±1	Yes	-32%
C_9-VVVAAAKKK-NH_2	2±0	Yes	78%
alpha linolenic acid-VVVAAAKKK-NH_2	1±0	No	ND
eicosapentaene acid -VVVAAAKKK-NH_2	12±1	Yes	99%
Fmoc-VVVAAAKKK-NH_2	5±1	Yes	28%
Nap-VVVAAAKKK-NH_2	1±1	Yes	ND

B

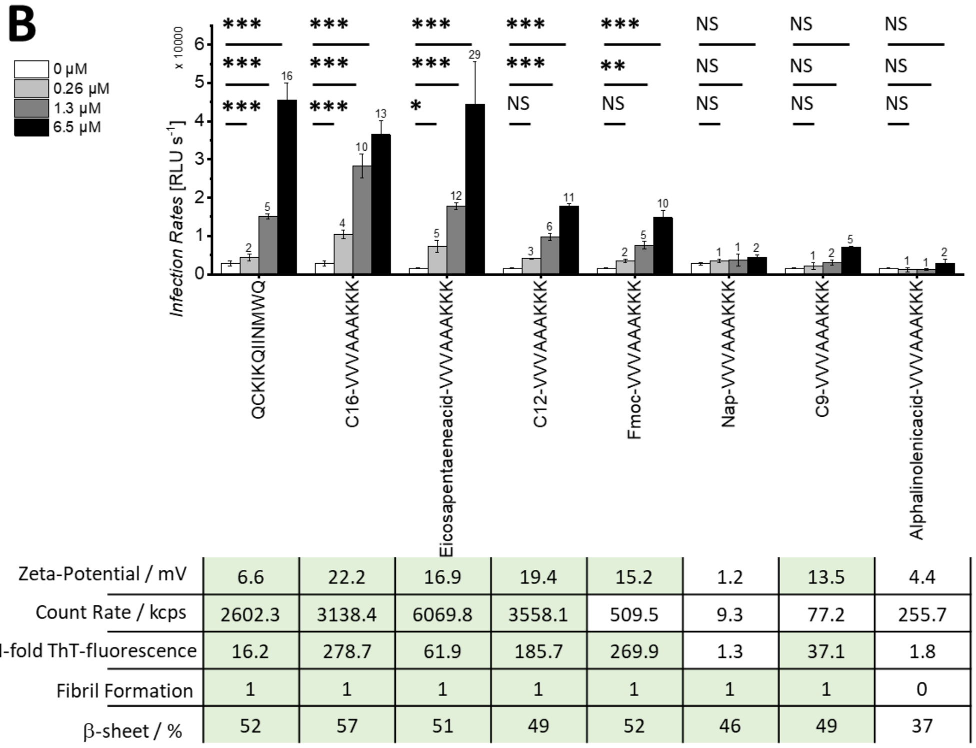

	QCKIKQIINMWQ	C16-VVVAAAKKK	Eicosapentaeneacid-VVVAAAKKK	C12-VVVAAAKKK	Fmoc-VVVAAAKKK	Nap-VVVAAAKKK	C9-VVVAAAKKK	Alphalinolenicacid-VVVAAAKKK
Zeta-Potential / mV	6.6	22.2	16.9	19.4	15.2	1.2	13.5	4.4
Count Rate / kcps	2602.3	3138.4	6069.8	3558.1	509.5	9.3	77.2	255.7
N-fold ThT-fluorescence	16.2	278.7	61.9	185.7	269.9	1.3	37.1	1.8
Fibril Formation	1	1	1	1	1	1	1	0
β-sheet / %	52	57	51	49	52	46	49	37

Figure S13 A Summary of sequence, infectivity enhancement at 1.3 µM concentration relative to virus only (n-fold), assembly into fibrils determined by TEM, **Figure S27** and degradability determined via confocal microscopy aggregate analysis of particles > 10 µm². Color code for sequence representation: Yellow hydrophobic moiety, blue cationic amino acids, red non-polar amino acids, purple polar amino acids, green anionic amino acids. **B** Infectivity assay showing HIV-1 infection rates of TZM-bl cells observed in the presence of increasing concentrations of peptides EF-C and newly designed PAs (6.5 µM, 1.3 µM, 0.26 µM). The baseline (0 µM) shows infection rate without the addition of peptides. Values above each bar represent the n-fold enhancement compared to 0 µM. Mean values and standard deviations are derived from three repetitions of triplicate infections. P-values for statistical significance were determined by two-way ANOVA test and are indicated with * $p \leq 0.05$, ** $p \leq 0.01$, *** $p \leq 0.001$, NS not significant. RLU s^{-1} are relative light units per second. The table summarizes the respective physicochemical properties of the PAs. PAs assembling into fibrils as determined by TEM (**Figure S27**) are indicated with "1". The β-sheet content is calculated from the FT-IR spectra (**Figure S28**). Properties which were previously found to favor infectivity are colored green.

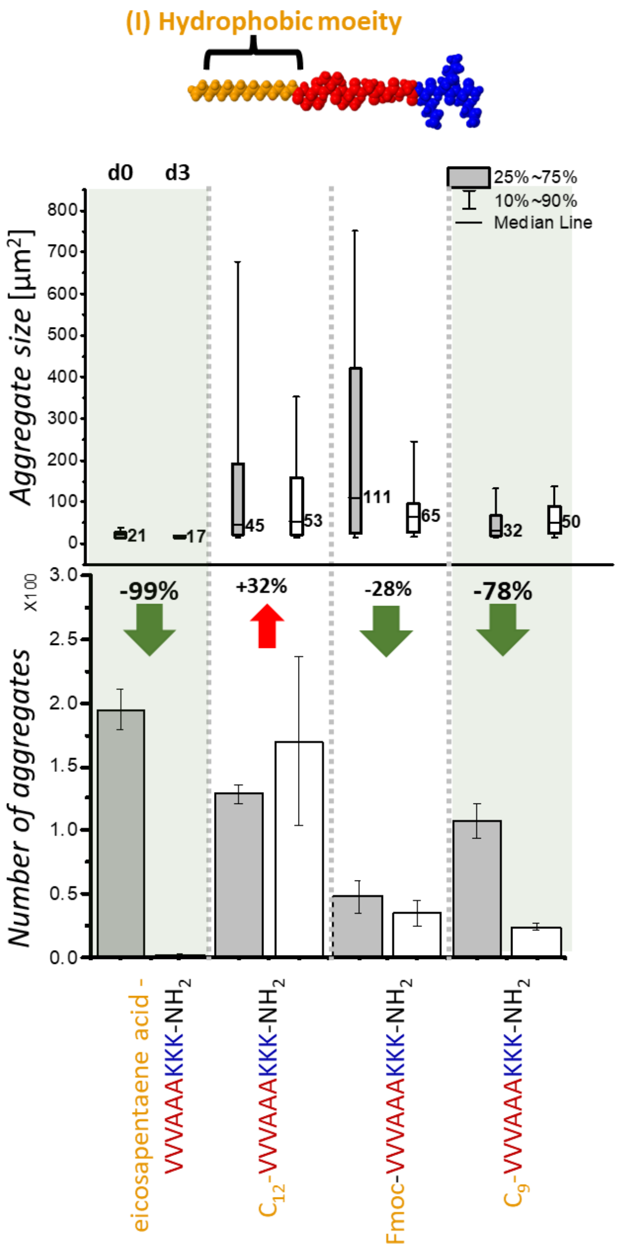

Figure S14 Degradability of PAs investigated via the aggregate size and number of aggregates larger than 10 µm^2 in an area of 1008 µm × 1008 µm. Error bars in aggregate number depict technical triplicate measurements. The percentual degradability was determined by number of aggregates after 3 days (d3, white boxes and bars) relative to the initial number of aggregates after 30 min incubation (d0 black boxes and bars). Box plots show aggregate size distribution with 10-90% shown as whiskers, 25-75% as boxes and the median line. The arrows give a qualitative evaluation of size reduce (green) or increase (red), number reduce (green) or number increase (red). Green highlighted section are pointing out best performing candidates for degradability.

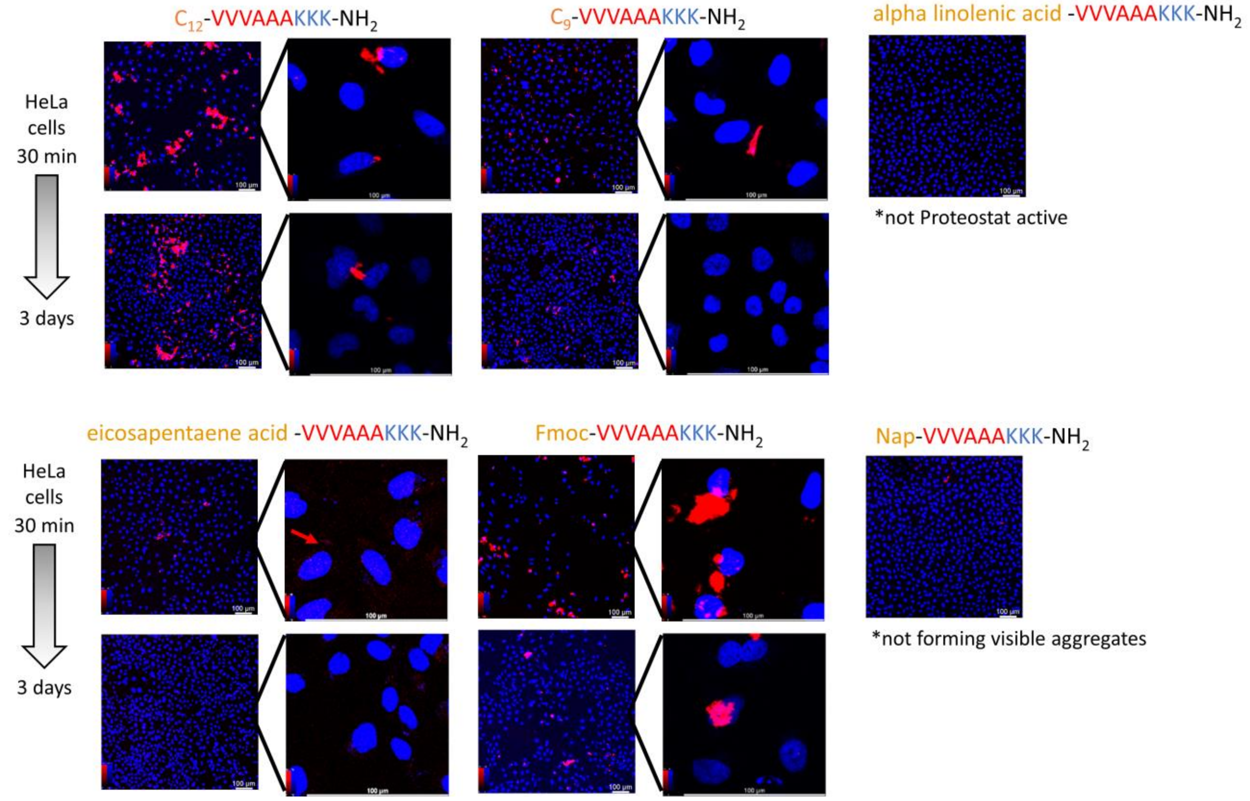

Figure S15 Representative confocal laser scanning microscopy images of Proteostat-labeled PAs (red) incubated with HeLa cells (nucleus in blue) after 30 minutes of incubation (top rows) and 3 days of incubation (bottom rows), scale bar 100 µm.

7.2 Effects of linker exchange on properties and activity

The linker is the functional group connecting the hydrophobic moiety with the peptide sequence. In the original sequence the linker is an amide bond. To investigate the effects of the linker on infectivity enhancement and degradability, we tested ester, thioester, disulfide and enzyme degradable peptide sequences GPLGV[13] (matrix metalloproteinase sensitive) and GFLG[14] (cathepsin B sensitive) (**Figure S30**) to investigate potential chemical and enzymatic degradation pathways.

Changing the linker does not significantly affect the infectivity enhancement for all tested functional groups except in the case of disulfide. The infectivity enhancement of the disulfide linker-containing PA is reduced approx. 60 % (n-fold at 1.3 µM reduced from 10 to 4). This decline in activity can be traced back to the missing fibril aggregation of the disulfide linked PA (C_{16}-*disulfide*-CVVVAAAKKK-NH_2) as is evidenced by low light scattering count rate (**Figure S16**). C_{16}-*disulfide*-CVVVAAAKKK-NH_2 can be stained with the imaging agent Proteostat (**Figure S26**), but not observed via microscopy (**Figure S18**), because it is not forming visible aggregates.

All investigated linker-modified PAs are degraded in the presence of cells (**Figure S17**). The best performing linker exchange is observed for the thioester modification, which shows a degradation of 87% and small, well distributed aggregates (**Figure S18**). The ester modified PA is also forming small aggregates and degraded well, but not as efficient as the thioester modified or PA1. The PAs with enzymatically degradable peptide sequences (MMP and cathepsin B) did not perform better than other linkers in terms of degradability (**Figure S17**). This may be because the peptide sequences are not sterically not accessible to the relevant enzymes.

A

(II) Linker

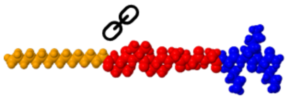

**Sequence**	**Infectivity enhancing n-fold at 1.3 µM**	**Fibril Formation**	**Degradability / % (rel. to initial particles > 10 µm²)**
C_{16}-VVVAAAKKK-NH_2	10±3	Yes	94%
C_{16}-*ester*-SVVVAAAKKK-NH_2	17±5	Yes	31%
C_{16}-*disulfide*-CVVVAAAKKK-NH_2	4±2	Yes	ND
C_{16}-*thioester*-CVVVAAAKKK-NH_2	11±4	Yes	**87**%
C_{16} -GPLGV-VVVAAAKKK-NH_2	8±3	Yes	28%
C_{16} –GFLG- VVVAAAKKK-NH_2	11±4	Yes	4%

B

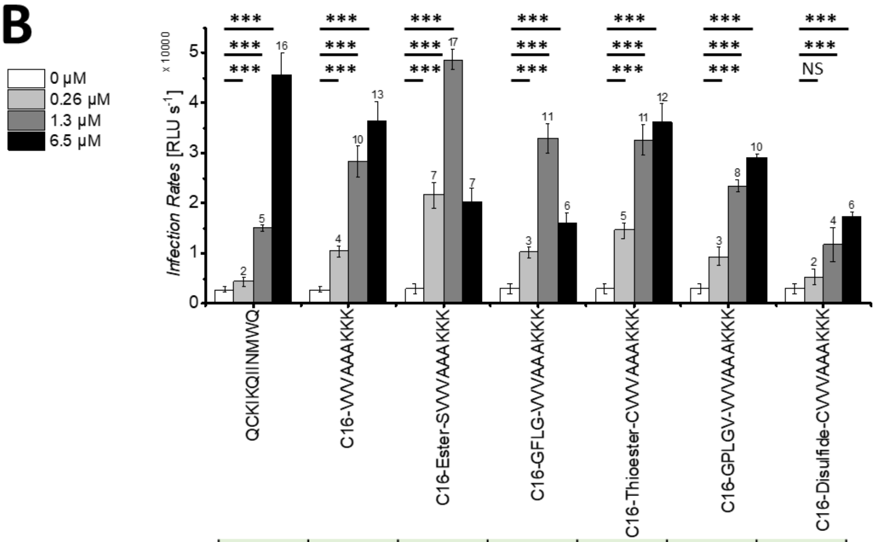

Zeta-Potential / mV	6.6	22.2	20.5	18.8	18.3	17.3	19.8
Count Rate / kcps	2602.3	3138.4	3217.3	5544.1	2058.5	5058.7	926.1
N-fold ThT-fluorescence	16.2	278.7	277.1	340.8	166.1	395.5	21.4
Fibril Formation	1	1	1	1	1	1	1
β-sheet / %	52	57	43	57	50	64	57

Figure S16 A Summary of sequence, infectivity enhancement at 1.3 µM concentration relative to virus only (n-fold), assembly into fibrils determined by TEM (**Figure S27**), and degradability determined via confocal microscopy aggregate analysis of particles > 10 µm^2. Color code for sequence representation: Yellow hydrophobic moiety, blue cationic amino acids, red non-polar amino acids, purple polar amino acids, green anionic amino acids. **B** Infectivity assay showing HIV-1 infection rates of TZM-bl cells observed in the presence of increasing concentrations of peptides EF-C and newly designed PAs (6.5 µM, 1.3 µM, 0.26 µM). The baseline (0 µM) shows infection rate without the addition of peptides. Values above each bar represent the n-fold enhancement compared to 0 µM. Mean values and standard deviations are derived from three repetitions of triplicate infections. P-values for statistical significance were determined by two-way ANOVA test and are indicated with * $p \leq 0.05$, ** $p \leq 0.01$, *** $p \leq 0.001$, NS not significant. RLU s^{-1} are relative light units per second. The table summarizes the respective physicochemical properties of the PAs. PAs assembling into fibrils as determined by TEM (**Figure S27**) are indicated with "1". The β-sheet content is calculated from the FT-IR spectra (**Figure S28**). Properties which were previously found to favor infectivity are colored green.

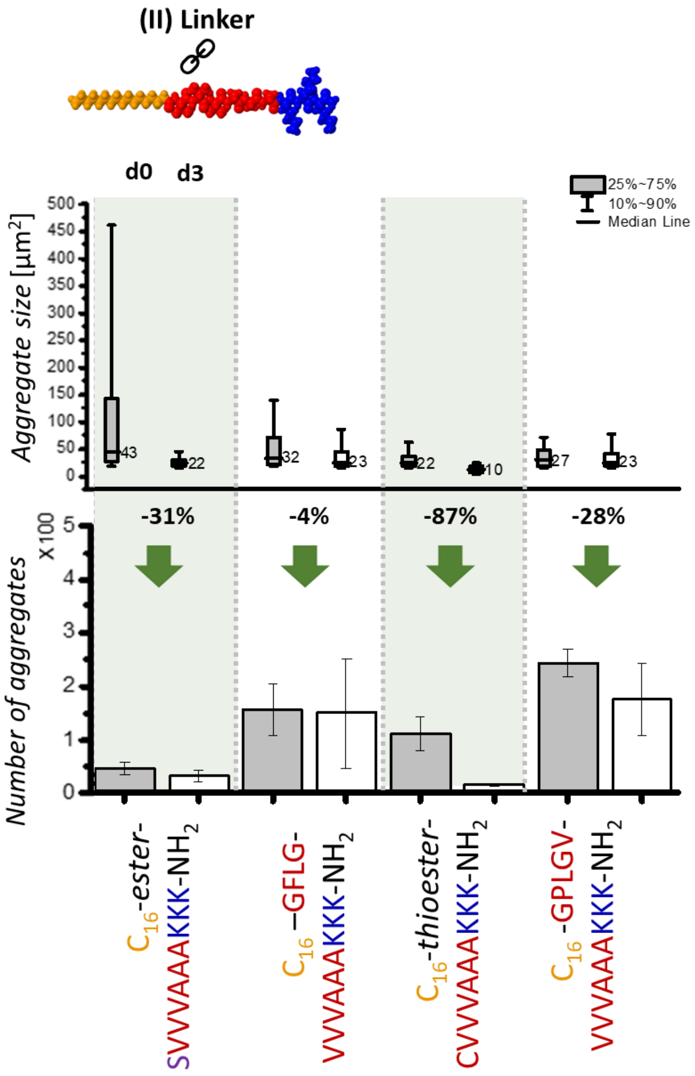

Figure S17 Degradability of PAs investigated via the aggregate size and number of aggregates larger than 10 µm^2 in an area of 1008 µm × 1008 µm. Error bars in aggregate number depict technical triplicate measurements. The percentual degradability was determined by number of aggregates after 3 days (d3, white boxes and bars) relative to the initial number of aggregates after 30 min incubation (d0 black boxes and bars). Box plots show aggregate size distribution with 10-90% shown as whiskers, 25-75% as boxes and the median line. The arrows give a qualitative evaluation of size reduce (green) or increase (red), number reduce (green) or number increase (red). Yellow circle means no great change from initial state. Green highlighted section is pointing out best performing candidates for degradability.

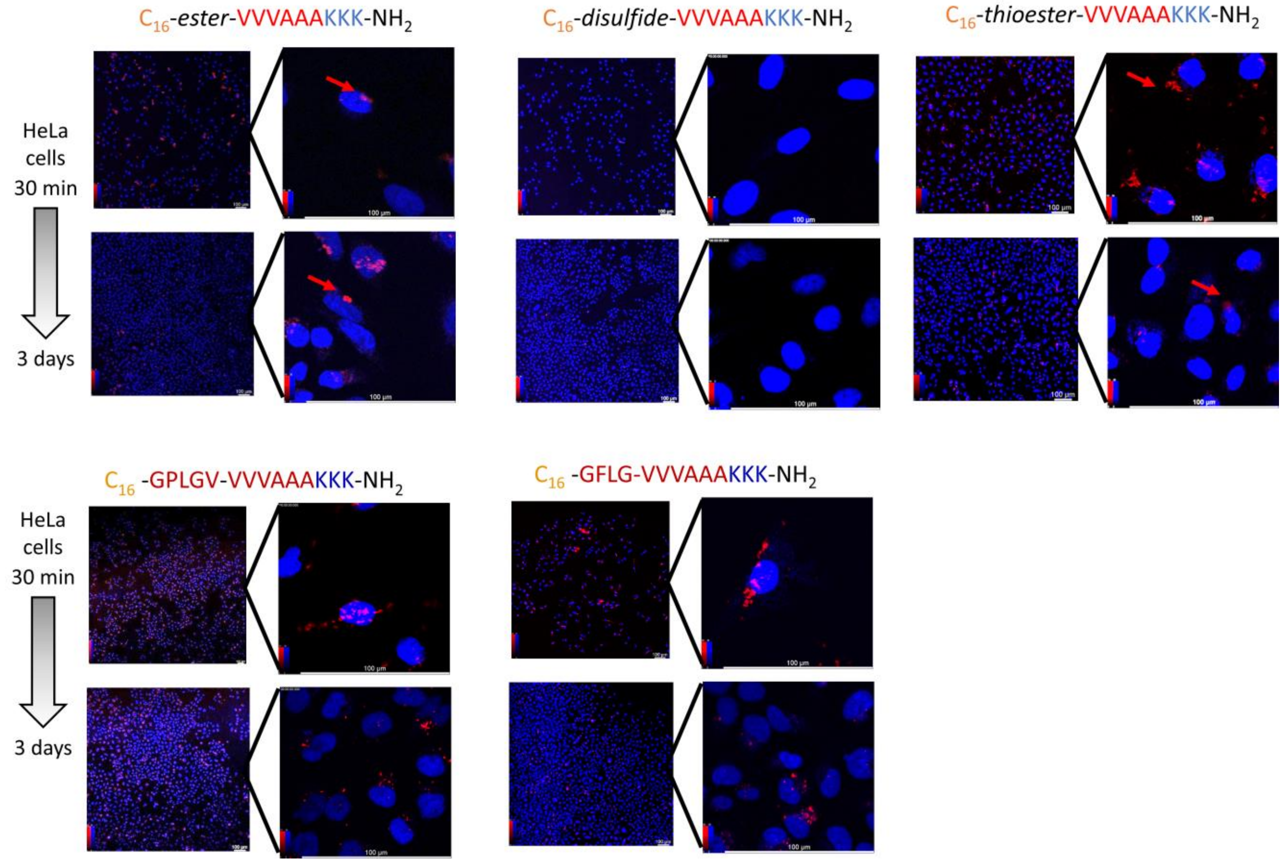

Figure S18 Representative confocal laser scanning microscopy images of Proteostat-labeled PAs (red) incubated with HeLa cells (nucleus in blue) after 30 minutes of incubation (top rows) and 3 days of incubation (bottom rows), scale bar 100 µm.

7.3 Effects of sequence exchange on properties and activity

Next, we evaluated if attaching a hydrophobic moiety to non-assembling peptides is a reliable way to achieve assembly into fibrils and infectivity enhancement, and what design rules for the sequence are important to obtain infectivity enhancing PAs (**Figure S19** – **Figure S22**). To this end, we attached palmitic acid to peptides (**Figure S31**, **Figure S32**) which are

(I) inspired from the original sequence design of PA1 (hydrophobic amino acids are close to palmitic acid residue, polar amino acids are mostly found at the C-terminus)

(II) known to have β-sheet prone sequences but are not assembling into fibrils without fatty acid at the investigated conditions. We selected peptide sequences which follow the design rule of PA1 (hydrophobic amino acids are close to palmitic acid residue, polar amino acids are mostly found at the C-terminus).
or

(III) endogenous peptide sequences which are found in β-sheet rich regions of proteins but are sequence-wise non-related to PA1.

The respective peptide sequences without palmitic acid were analyzed analogously (**Figure S23**, **Figure S24**).

We found that attaching palmitic acid to the *N*-terminus of non-assembling peptides is a very reliable way to create peptide amphiphiles which form fibrils (90%, 19 of 21 tested compounds assembled into fibrils, **Figure S19**, **Figure S27**). The only peptide sequence which are not forming fibrils after functionalization with palmitic acid are C_{16}-KGVPGVGK-NH_2 and C_{16}-WEALKK-NH_2. Both sequences include charged amino acids (K or E) close to the hydrophobic moiety, which might disturb self-assembly. Moreover, all the PAs except of C_{16}-KGVPGVGK showed a positive Zeta-potential, while the corresponding peptides without fatty acid moiety showed negative or almost neutral (< 5 mV) zeta-potential. Attaching palmitic acid to net positive charged peptide sequences is therefore a reliable way to obtain nanostructures with positive Zeta-potential.

Strikingly, 11 of the 21 newly created PAs with different sequences show infectivity enhancement (n fold > 2 at 1.3 µM) with similar or less activity compared to PA1. No significant difference regarding to design principles (I-III) could be found, but all the non-active (n-fold at 1.3 µM < 2) and weakly active (n-fold at 1.3 µM 2–3) PAs had in common that they are not forming µm-sized aggregates and forming too few/less aggregates, respectively (**Figure S21** - **Figure S22**: C_{16}-WEALKK-NH_2, C_{16}-AGGRVK-NH_2, C_{16}-AVAKKK-NH_2, C_{16}-RRWQWR-NH_2, C_{16}-KGVPGVGK-NH_2, C_{16}-GGGSSKKK-NH_2, C_{16}-GGVVRR-NH_2, C_{16}-AGAGRRR-NH_2).

PAs that form no or too small aggregates have in common that they contain a high number of flexible amino acids (G, A) and polar amino acids (R, K, E) close to the fatty acid. This could increase dynamicity and solvation of the self-assembling PAs, which result in less aggregating fibers. Further, sterically demanding amino acids (W) close the fatty acid moiety and kinked amino acids (P) in the sequence hinder efficient assembly, which can increase dynamicity of the fibers and result in less aggregation.

On the other side having few, large aggregates (approximately less than 100 aggregates per 1008 µm × 1008 µm with more than 10% larger than 200 µm) reduces infectivity enhancement and

degradability. PAs that are forming these large aggregates as observed via confocal microscopy aggregate size analysis (**Figure S21**- **Figure S22**) are C16-AQAKAK, C_{16}-NFYLQVNKINK-NH_2, C_{16}-KYIHQNYTKALAGKLV-NH_2, C_{16}-KIISFK-NH_2, C_{16}-AAVVHHK-NH_2, and C_{16}-VVAAHHH-NH_2 form few but very large aggregates.

Most of the PAs that form very large aggregates have in common, that their sequences are composed of alternating, amphiphilic amino acids, comparable to PA2, that also forms large aggregates that cannot degraded well (**Figure 4**). C_{16}-VVAAHHH-NH_2 does not contain any charged amino acid at pH 7.4 and C_{16}-AAVVHHK-NH contains only one cationic charged residue. Aggregation is likely caused due to the hydrophobic, aromatic character of histidine (H) at the C-terminus, which decreases solubility and favors fiber-fiber aggregation.

Further, most of the PAs that form very large aggregates are degraded incompletely as evidenced by decrease of aggregate size but increase in aggregate number for C_{16}-AAVVHHK-NH_2, C_{16}-NFYLQVNKINK-NH_2, C_{16}-KYIHQNYTKALAGKLV-NH_2, C_{16}-KIISFK-NH_2, C16-AQAKAK. This observation could indicate a fragmentation of large aggregates that occurs simultaneously to the degradation. The opposite case (increase in size, decrease in number) indicates an aggregation of small particles to larger particles, and can be observed for C_{16}-VVAAHHH-NH_2 and C_{16}-RYYASLRHYLNLVTRQRY-NH_2. Both cases are not desired in *in vitro* applications, thus these PAs are not well suited as degradable additives to enhance viral infectivity.

The best performing candidates in terms of infectivity and degradability are C_{16}-AKAVGK-NH_2 (infect *n* fold = 6, degradability 89%), then C_{16}-LNGGVKK-NH_2 (infect *n* fold = 6, degradability 48%) and finally C_{16}-ALAAGKK-NH_2 (infect *n* fold = 5, degradability 87%).

Noteworthy the peptides NFYLQVNKINK-NH_2, RYYASLRHYLNLVTRQRY-NH_2 and KGVPGVGK-NH_2 are forming fibrils even without palmitic acid. With the exception of NFYLQVNKINK-NH_2 these peptides were found to be non-aggregating and inactive (**Figure S23**, **Figure S24**). The sole active sequence (NFYLQVNKINK-NH_2) also showed the required physicochemical properties but no good degradability (**Figure S24**).

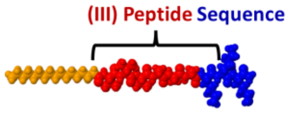

Sequence	Infectivity enhancing n-fold at 1.3 µM	Fibril Formation	Degradability / % (rel. to initial particles > 10 µm²)	Sequence Design Information
C_{16}-VVVAAAKKK-NH_2	10±3	Yes	94%	Original Sequence
C_{16}-AVAKKK-NH_2	2±0	Yes	94%	inspired **from original sequence**
C_{16}-VVAAHHH-NH_2	2±0	Yes	26%	inspired **from original sequence**
C_{16}-GGGSSKKK-NH_2	2±1	Yes	ND	inspired **from original sequence**
C_{16}-AGAGRRR-NH_2	1±0	Yes	ND	inspired **from original sequence**
C_{16}-AAVVHHK-NH_2	6±3	Yes	-123%	inspired **from original sequence**
C_{16}-GGVVRR-NH_2	1±1	Yes	ND	inspired **from original sequence**
C_{16}-IIQKIK-NH_2	4±1	Yes	28%	**non-assembling**, β-sheet prone sequence
C_{16}-AQAKAK-NH_2	4±1	Yes	-83%	**non-assembling**, β-sheet prone sequence
C_{16}-LLLKK-NH_2	3±0	Yes	27%	peptide **not assembling** by its own, but related derivatives (β–sheet prone)
C_{16}-KGVPGVGK-NH_2	1±0	No	ND	peptide **not assembling** by its own, but related derivatives (β-sheet prone)
C_{16}-AGGRVK-NH_2	3±1	Yes	ND	**Endogeneous**<br>Inspired from AGGRV<br>(138-142 T-cell receptor on myeloid cells)
C_{16}-LNGGVKK-NH_2	6±1	Yes	48%	**Endogeneous**<br>Inspired from 116-121 EGFR factor
C_{16}-ALAAGKK-NH_2	5±1	Yes	87%	**Endogeneous**<br>Reverse seq of KGAALA: 105-110 Linoleate 9S-lipoxygenase
C_{16}-WEALKK-NH_2	1±0	No	ND	**Endogeneous**<br>Reverse sequence of KKLAEW: 155-160 Protein phosphatase 1D
C_{16}-RRWQWR-NH_2	0±0	Yes	ND	**Endogeneous**<br>Derived from cell-penetrating peptide of bovine lactoferrin
C_{16}-KYIHQNYTKALAGKLV-NH_2	5±2	Yes	-110%	**Endogeneous**<br>532 - 548 sequence from procollagen I
C_{16}-NFYLQVNKINK-NH_2	5±1	Yes	-123%	**Endogeneous**<br>282- 293 sequence from lumican
C_{16}-RYYASLRHYLNLVTRQRY-NH_2	5±1	Yes	43%	**Endogeneous**<br>46 - 63 PYY gut hormone truncated
C_{16}-GKVGSK -NH_2	2±1	Yes	ND	**Endogeneous**<br>Serumalbumin derived
C_{16}-AKAVGK-NH_2	6±3	Yes	89%	**Endogeneous**<br>GAPDH derived
C_{16}-KIISFK-NH_2	2±1	Yes	-84%	**Endogeneous**<br>IL-18 derived

Figure S19 Summary of sequence, infectivity enhancement at 1.3 µM concentration relative to virus only (n-fold), assembly into fibrils determined by TEM (**Figure S27**), and degradability determined via confocal microscopy aggregate analysis of particles > 10 µm^2. Additionally, sequence design information describes the rationally behind the sequence selection. Color code for sequence representation: Yellow hydrophobic moiety, blue cationic amino acids, red non-polar amino acids, purple polar amino acids, green anionic amino acids.

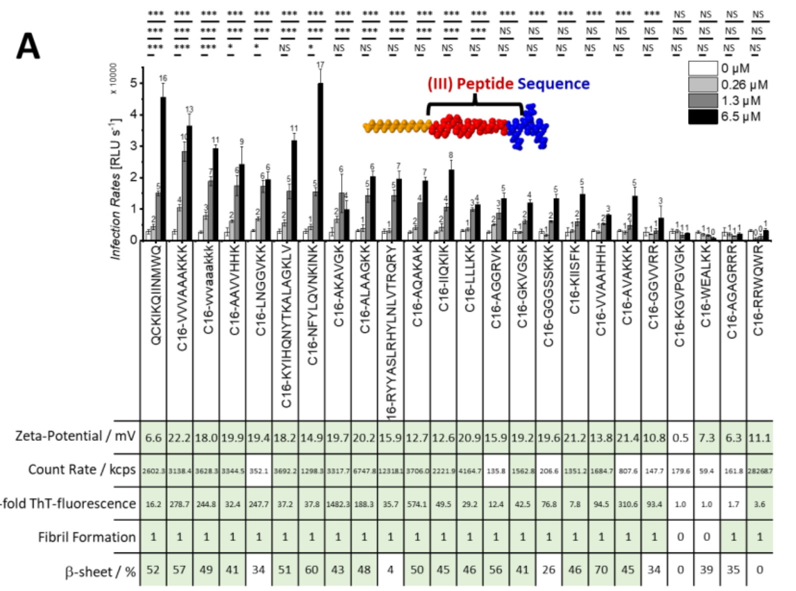

	QCKIKQIINMWQ	C16-VVVAAAKKK	C16-vvvaaakkk	C16-AAVVHHK	C16-LNGGVKK	C16-KYIHQNYTKALAGKLV	C16-NFYLQVNKINK	C16-AKAVGK	C16-ALAAGKK	16-RYYASLRHYLNLVTRQRY	C16-AQAKAK	C16-IIQKIK
Zeta-Potential / mV	6.6	22.2	18.0	19.9	19.4	18.2	14.9	19.7	20.2	15.9	12.7	12.6
Count Rate / kcps	2602.3	3138.4	3628.3	3344.5	352.1	3692.2	1298.3	3317.7	6747.8	12318.1	3706.0	2221.9
N-fold ThT-fluorescence	16.2	278.7	244.8	32.4	247.7	37.2	37.8	1482.3	188.3	35.7	574.1	49.5
Fibril Formation	1	1	1	1	1	1	1	1	1	1	1	1
β-sheet / %	52	57	49	41	34	51	60	43	48	4	50	45

	C16-LLLKK	C16-AGGRVK	C16-GKVGSK	C16-GGGSSKKK	C16-KIISFK	C16-VVAAHHH	C16-AVAKKK	C16-GGVVRR	C16-KGVPGVGK	C16-WEALKK	C16-AGAGRRR	C16-RRWQWR
Zeta-Potential / mV	20.9	15.9	19.2	19.6	21.2	13.8	21.4	10.8	0.5	7.3	6.3	11.1
Count Rate / kcps	4164.7	135.8	1562.8	206.6	1351.2	1684.7	807.6	147.7	179.6	59.4	161.8	28268.7
N-fold ThT-fluorescence	29.2	12.4	42.5	76.8	7.8	94.5	310.6	93.4	1.0	1.0	1.7	3.6
Fibril Formation	1	1	1	1	1	1	1	1	0	0	1	1
β-sheet / %	46	56	41	26	46	70	45	34	0	39	35	0

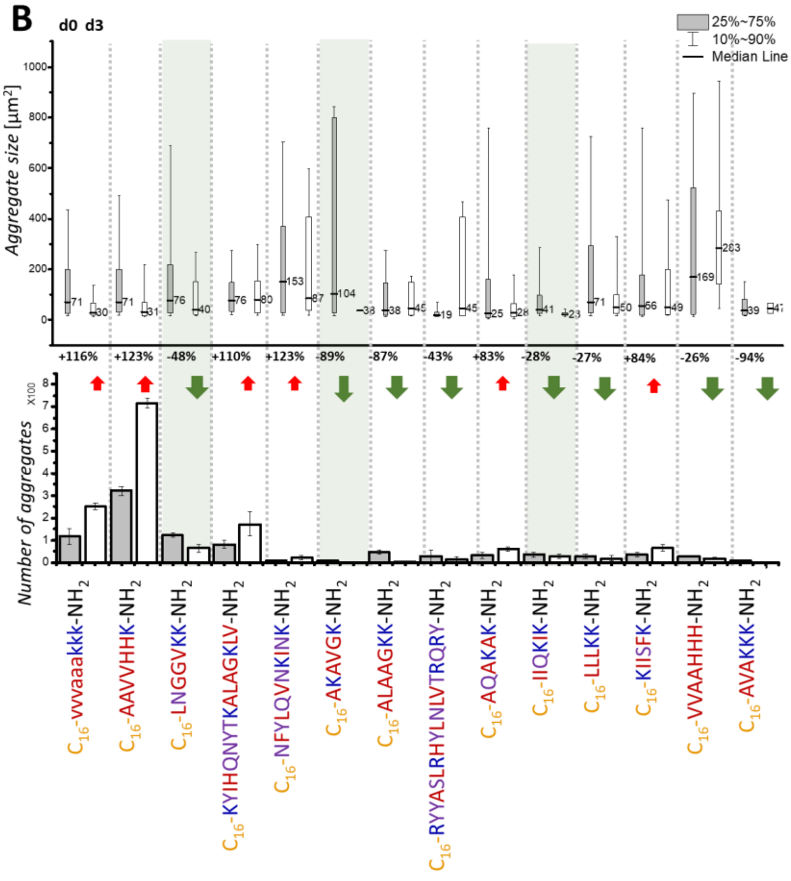

Figure S20 A Infectivity assay showing HIV-1 infection rates of TZM-bl cells observed in the presence of increasing concentrations of peptides EF-C and newly designed PAs (6.5 µM, 1.3 µM, 0.26 µM). The baseline (0 µM) shows infection rate without the addition of peptides. Values above each bar represent the n-fold enhancement compared to 0 µM. Mean values and standard deviations are derived from three repetitions of triplicate infections. P-values for statistical significance were determined by two-way ANOVA test and are indicated with * $p \leq 0.05$, ** $p \leq 0.01$, *** $p \leq 0.001$, NS not significant.RLU s^{-1} are relative light units per second. The table summarizes the respective physicochemical properties of the PAs. PAs assembling into fibrils as determined by TEM (**Figure S27**) are indicated with “1”. The β-sheet content is calculated from the FT-IR spectra (**Figure S28**). Properties which were previously found to favor infectivity are colored green. **B** Degradability of PAs investigated via the aggregate size and number of aggregates larger than 10 µm^2 in an area of 1008 µm × 1008 µm. Error bars in aggregate number depict technical triplicate measurements. The percentual degradability was determined by number of aggregates after 3 days (d3, white boxes and bars) relative to the initial number of aggregates after 30 min incubation (d0 black boxes and bars). Box plots show aggregate size distribution with 10-90% shown as whiskers, 25-75% as boxes and the median line. The arrows give a qualitative evaluation of size reduce (green) or increase (red), number reduce (green) or number increase (red). Green highlighted section is pointing out best performing candidates for degradability.

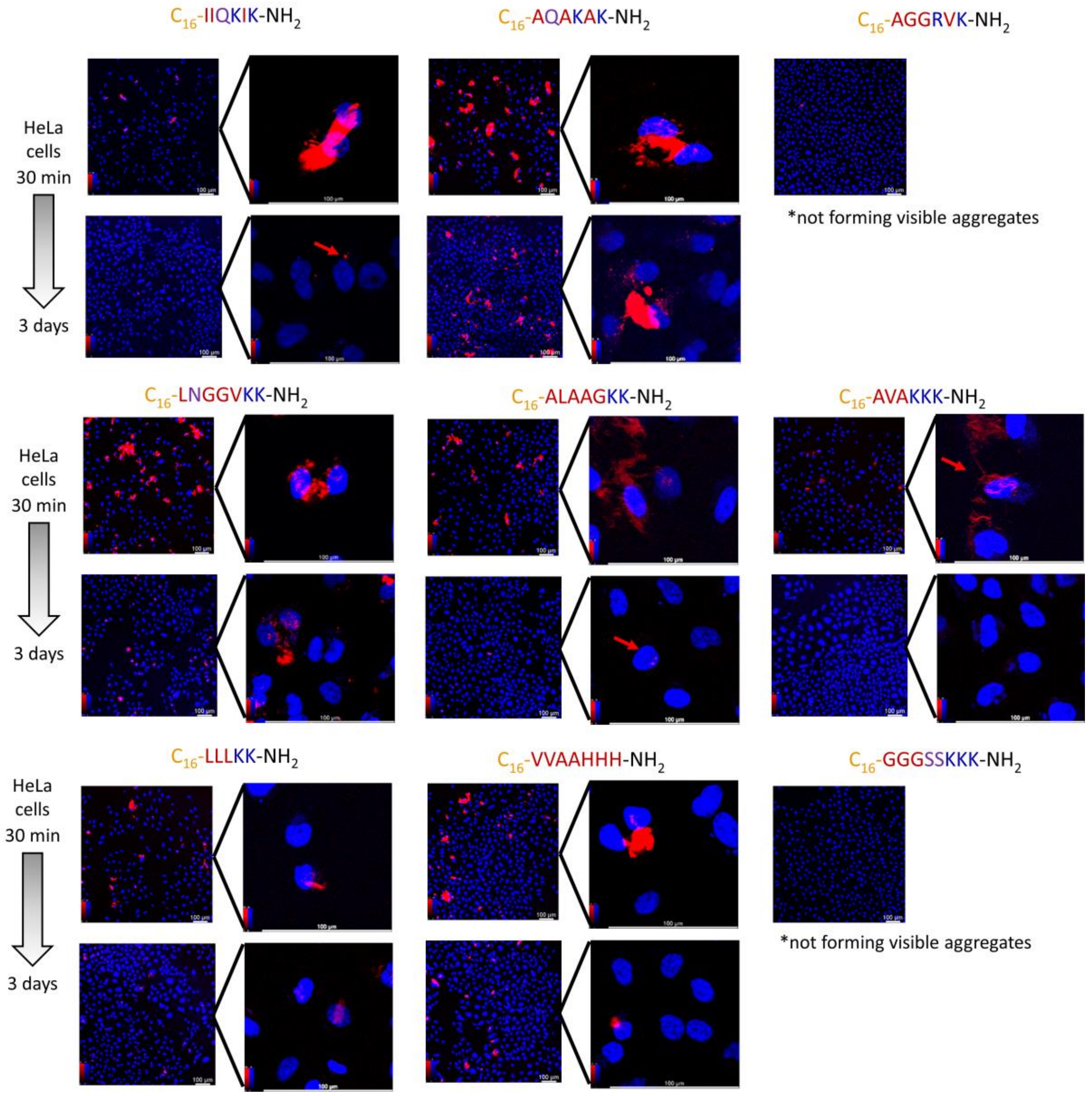

Figure S21 Representative confocal laser scanning microscopy images of Proteostat-labeled PAs (red) incubated with HeLa cells (nucleus in blue) after 30 minutes of incubation (top rows) and 3 days of incubation (bottom rows), scale bar 100 µm.

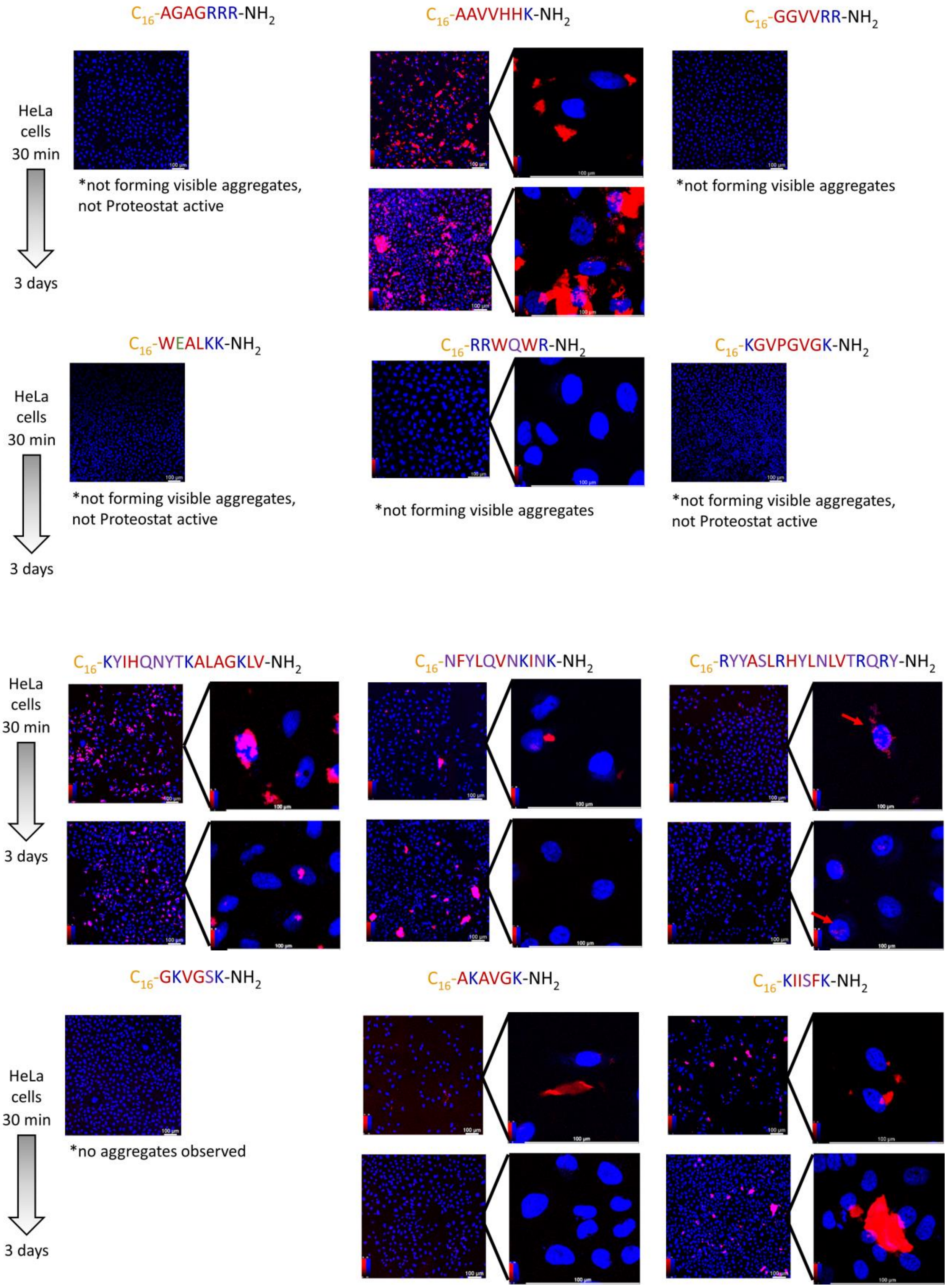

Figure S22 Representative confocal laser scanning microscopy images of Proteostat-labeled PAs (red) incubated with HeLa cells (nucleus in blue) after 30 minutes of incubation (top rows) and 3 days of incubation (bottom rows), scale bar 100 µm.

(III) Peptide Sequence control without fatty acid

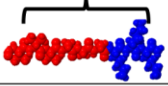

Sequence	Infectivity enhancing n-fold at 1.3 µM	Fibril Formation	Degradability / % (rel. to initial particles > 10 µm²)	Sequence Design Information
C_{16}-VVVAAAKKK-NH_2	10±3	Yes	94%	Original Sequence
VVVAAAKKK-NH_2	0.5±0.3	No	ND	
AVAKKK-NH_2	1.2±0.2	No	ND	inspired **from original sequence**
VVAAHHH-NH_2	0.7±0.1	No	ND	inspired **from original sequence**
GGGSSKKK-NH_2	1.0±0.3	No	ND	inspired **from original sequence**
AGAGRRR-NH_2	0.3±0.1	No	ND	inspired **from original sequence**
AAVVHHK-NH_2	0.8±0.3	No	ND	inspired **from original sequence**
GGVVRR-NH_2	0.8±0.6	No	ND	inspired **from original sequence**
IIQKIK-NH_2	1.2±0.4	No	ND	**non-assembling**, β-sheet prone sequence
AQAKAK-NH_2	0.6±0.2	No	ND	**non-assembling**, β-sheet prone sequence
LLLKK-NH_2	0.8±0.3	No	ND	peptide **not assembling** by its own, but related derivatives (β-sheet prone)
KGVPGVGK-NH_2	0.9±0.1	No	ND	peptide **not assembling** by its own, but related derivatives (β-sheet prone)
AGGRVK-NH_2	0.7±0.2	No	ND	**Endogeneous**<br>Inspired from AGGRV<br>(138-142 T-cell receptor on myeloid cells)
LNGGVKK-NH_2	0.8±0.2	No	ND	**Endogeneous**<br>Inspired from 116-121 EGFR factor
ALAAGKK-NH_2	1.0±0.3	No	ND	**Endogeneous**<br>Reverse seq of KGAALA: 105-110 Linoleate 9S-lipoxygenase
WEALKK-NH_2	0.8±0.2	No	ND	**Endogeneous**<br>Reverse sequence of KKLAEW: 155-160 Protein phosphatase 1D
RRWQWR-NH_2	0.8±0.3	No	ND	**Endogeneous**<br>Derived from cell-penetrating peptide of bovine lactoferrin
KYIHQNYTKALAGKLV-NH_2	1.2±0.5	No	ND	**Endogeneous**<br>532 - 548 sequence from procollagen I
NFYLQVNKINK-NH_2	7.9±1.7	Yes	10%	**Endogeneous**<br>282- 293 sequence from lumican
RYYASLRHYLNLVTRQRY-NH_2	1.9±1.0	Yes	ND	**Endogeneous**<br>46 - 63 PYY gut hormone truncated
GKVGSK -NH_2	0.8±0.3	No	ND	**Endogeneous**<br>Serumalbumin derived
AKAVGK-NH_2	0.9±0.3	No	ND	**Endogeneous**<br>GAPDH derived
KIISFK-NH_2	0.9±0.3	No	ND	**Endogeneous**<br>IL-18 derived

Figure S23 Summary of sequence, infectivity enhancement at 1.3 µM concentration relative to virus only (n-fold), assembly into fibrils determined by TEM (**Figure S27**), and degradability determined via confocal microscopy aggregate analysis of particles > 10 µm². Additionally, sequence design information describes the rationally behind the sequence selection. Color code for sequence representation: Yellow hydrophobic moiety, blue cationic amino acids, red non-polar amino acids, purple polar amino acids, green anionic amino acids.

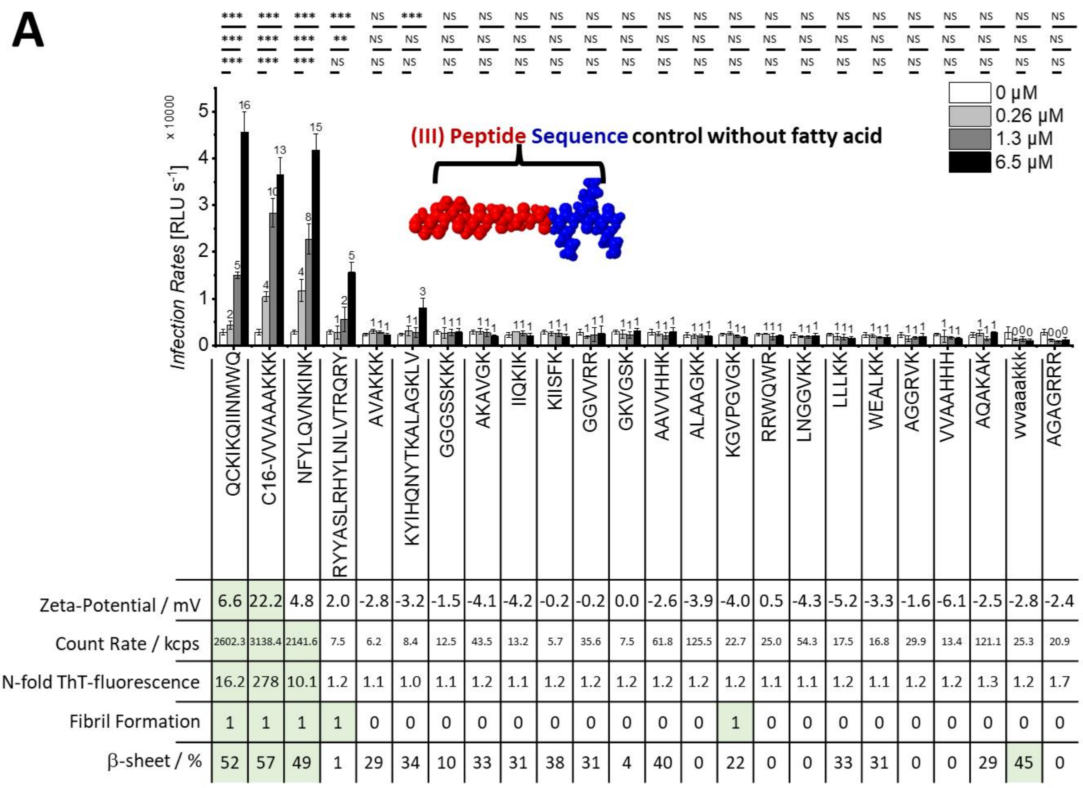

	QCKIKQIINMWQ	C16-VVVAAAKK	NFYLQVNKINK	RYYASLRHYLNLVTRQRY	AVAKKK	KYIHQNYTKALAGKLV	GGGSSKKK	AKAVGK	IIQKIK	KIISFK	GGVVRR	GKVGSK
Zeta-Potential / mV	6.6	22.2	4.8	2.0	-2.8	-3.2	-1.5	-4.1	-4.2	-0.2	-0.2	0.0
Count Rate / kcps	2602.3	3138.4	2141.6	7.5	6.2	8.4	12.5	43.5	13.2	5.7	35.6	7.5
N-fold ThT-fluorescence	16.2	278	10.1	1.2	1.1	1.0	1.1	1.2	1.1	1.2	1.2	1.2
Fibril Formation	1	1	1	1	0	0	0	0	0	0	0	0
β-sheet / %	52	57	49	1	29	34	10	33	31	38	31	4

	AAVVHHK	ALAAGKK	KGVPGVGK	RRWQWR	LNGGVKK	LLLKK	WEALKK	AGGRVK	VVAAHHH	AQAKAK	wvaaakk	AGAGRRR
Zeta-Potential / mV	-2.6	-3.9	-4.0	0.5	-4.3	-5.2	-3.3	-1.6	-6.1	-2.5	-2.8	-2.4
Count Rate / kcps	61.8	125.5	22.7	25.0	54.3	17.5	16.8	29.9	13.4	121.1	25.3	20.9
N-fold ThT-fluorescence	1.2	1.2	1.2	1.1	1.1	1.2	1.1	1.2	1.2	1.3	1.2	1.7
Fibril Formation	0	0	1	0	0	0	0	0	0	0	0	0
β-sheet / %	40	0	22	0	0	33	31	0	0	29	45	0

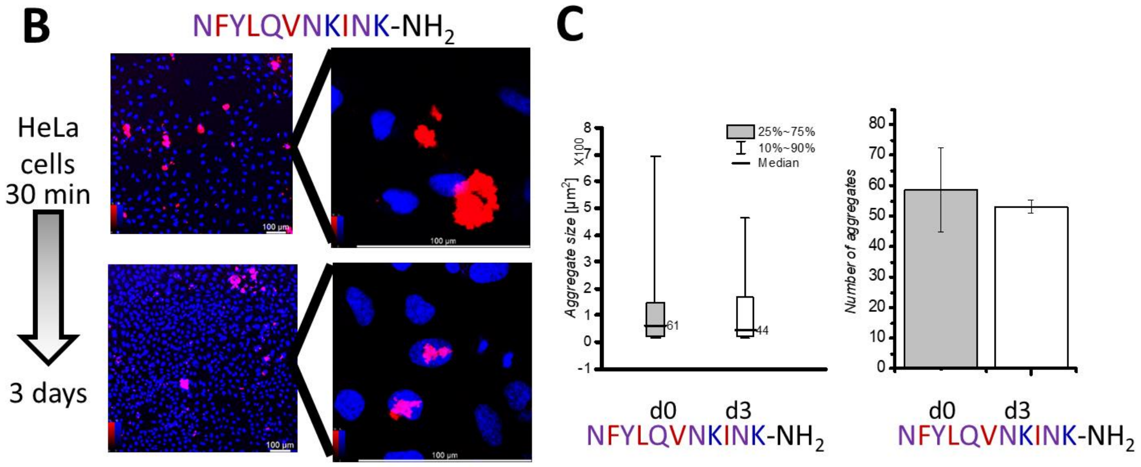

Figure S24 A Infectivity assay showing HIV-1 infection rates of TZM-bl cells observed in the presence of increasing concentrations of peptides EF-C and newly designed PAs (6.5 µM, 1.3 µM, 0.26 µM). The baseline (0 µM) shows infection rate without the addition of peptides. Values above each bar represent the n-fold enhancement compared to 0 µM. Mean values and standard deviations are derived from three repetitions of triplicate infections. P-values for statistical significance were determined by two-way ANOVA test and are indicated with * $p \leq 0.05$, ** $p \leq 0.01$, *** $p \leq 0.001$, NS not significant. RLU s^{-1} are relative light units per second. The table summarizes the respective physicochemical properties of the PAs. PAs assembling into fibrils as determined by TEM (**Figure S27**) are indicated with "1". The β-sheet content is calculated from the FT-IR spectra (**Figure S28**). Properties which were previously found to favor infectivity are colored green. **B** Representative confocal laser scanning microscopy images of Proteostat-labeled PA (red) incubated with HeLa cells (nucleus in blue) after 30 minutes of incubation (top row) and 3 days of incubation (bottom row), scale bar 100 µm. **C** Degradability of Pat46 investigated via the aggregate size and number of aggregates larger than 10 µm² in an area of 1008 µm × 1008 µm. Error bars in aggregate number depict technical triplicate measurements. The percentual degradability was determined by number of aggregates after 3 days (d3, white boxes and bars) relative to the initial number of aggregates after 30 min incubation (d0 black boxes and bars). Box plots show aggregate size distribution with 10-90% shown as whiskers, 25-75% as boxes and the median line.

8 Cell-Viability of PA library

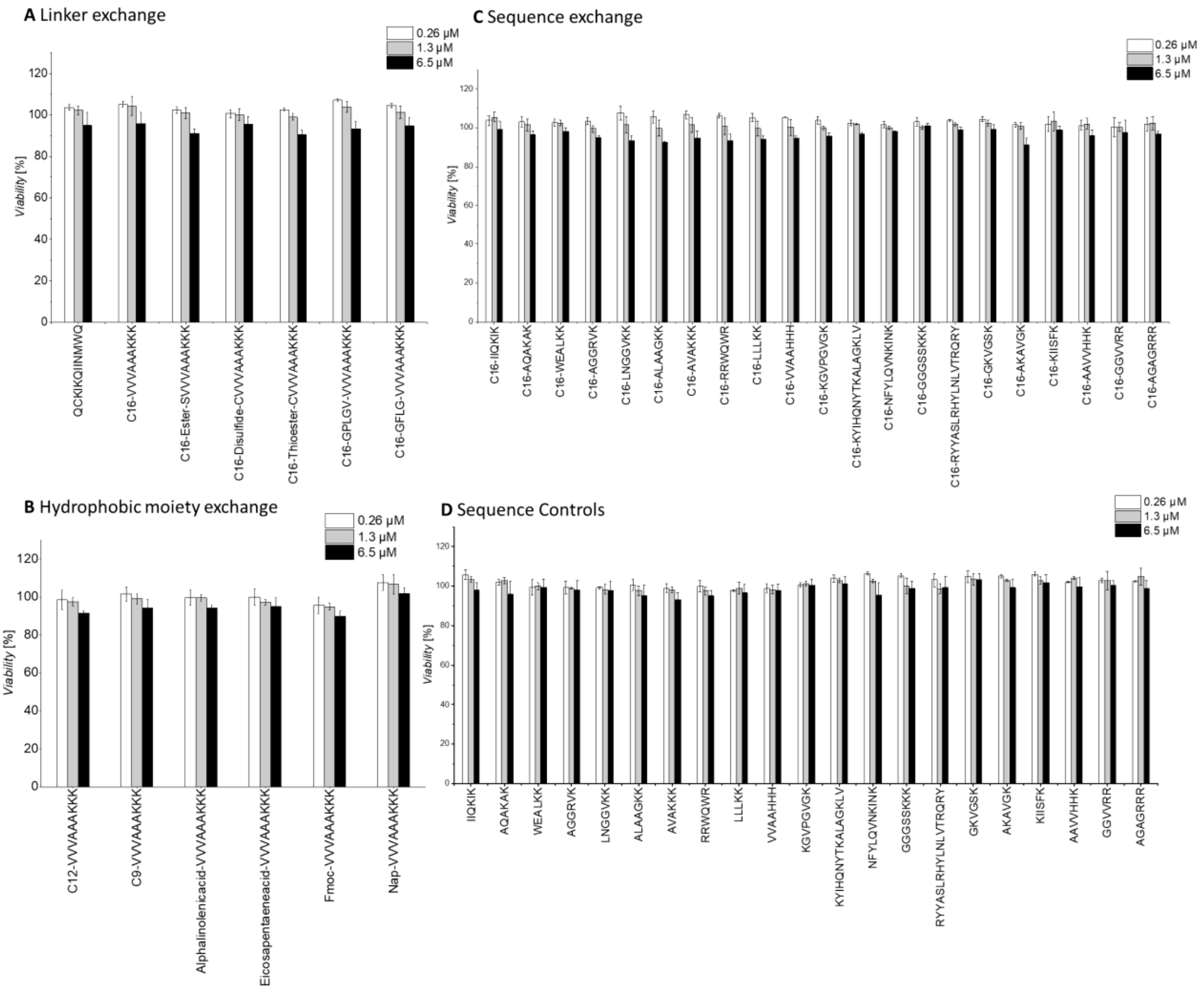

Figure S25 CellTiter-Glo cell viability assay of TZM-bl cells treated with increasing concentrations (0.26, 1.3 and 6.5 µM) of the peptides of biological triplicate with standard deviation.

9 Proteostat Assay of PA library

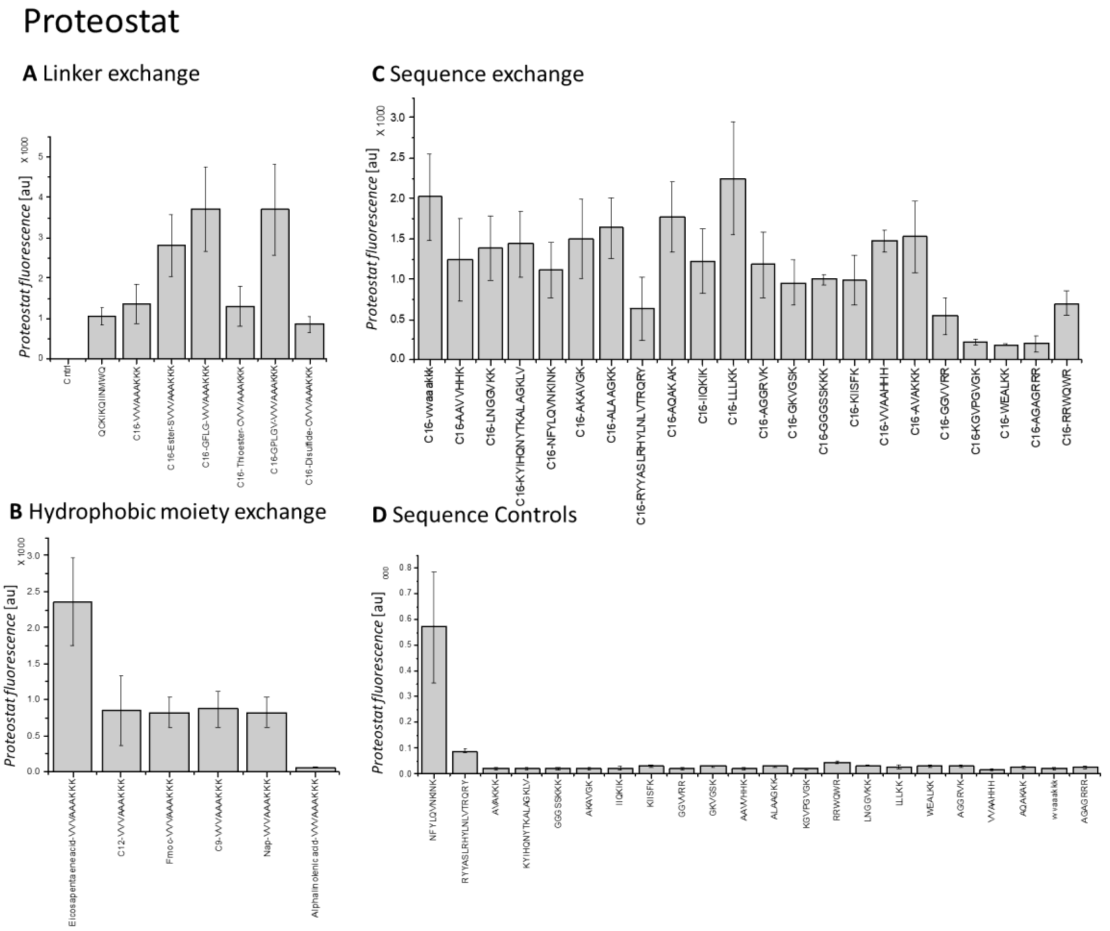

Figure S26 Proteostat Assay on PAs and peptides. **A** Linker exchange includes original sequence PA1 and variants with different linkers. For reference also EF-C and Control (PBS with 10% DMSO) are shown. **B** Exchange of hydrophobic moiety, **C** Exchange of peptide sequence **D** Controls showing peptide sequences without fatty acid residues.

10 TEM of PA library

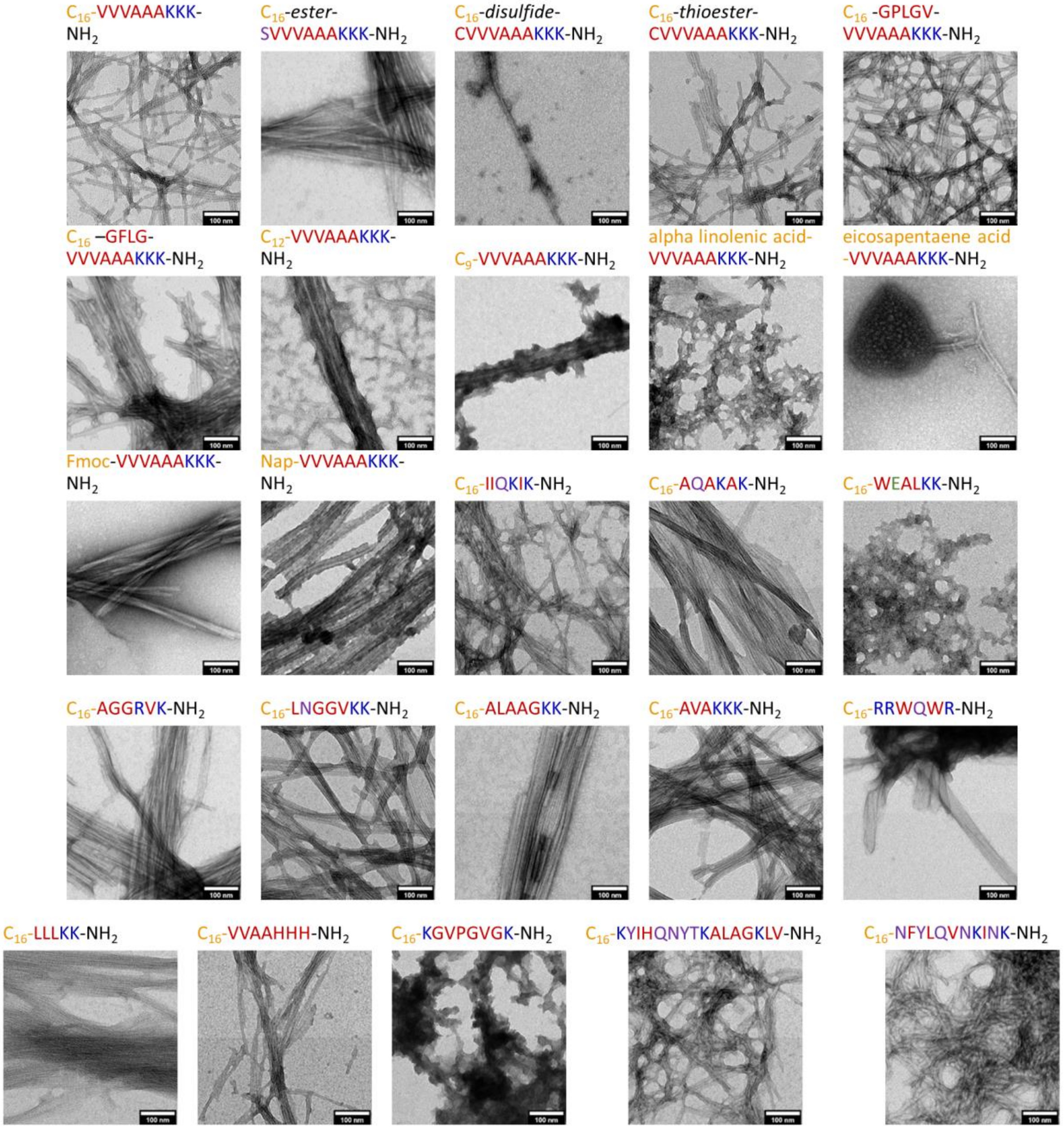

Figure S27 TEM micrographs of the PA and peptide library, scale bar 100 nm. The compounds were diluted from DMSO (10 mg mL^{-1}) in PBS (1 mg mL^{-1}) and incubated for 1d at rt. Color code for sequence representation: Yellow hydrophobic moiety, blue cationic amino acids, red non-polar amino acids, purple polar amino acids, green anionic amino acids.

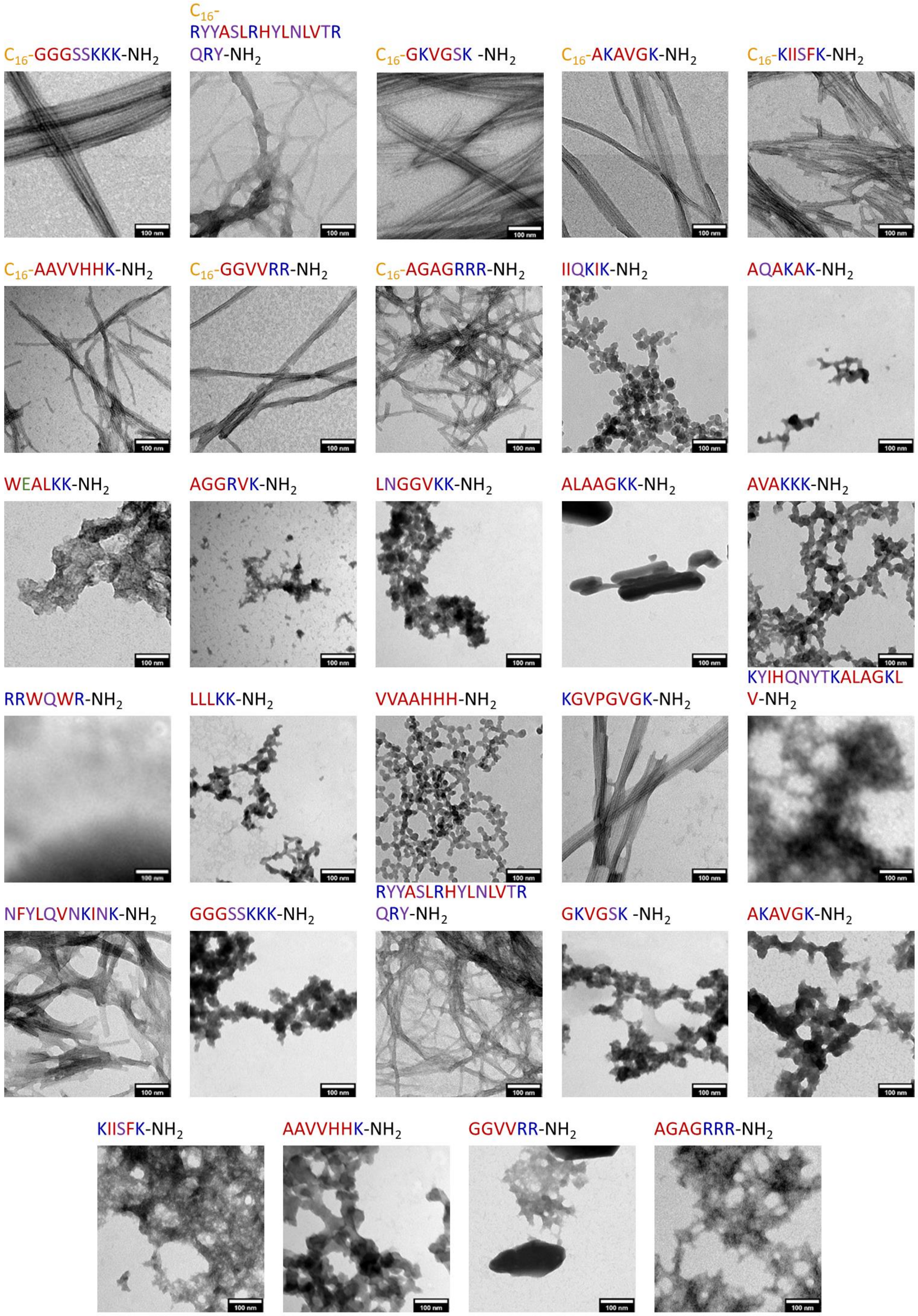

Figure S27 cont. TEM micrographs of the PA and peptide library, scale bar 100 nm. The compounds were diluted from DMSO (10 mg mL^{-1}) in PBS (1 mg mL^{-1}) and incubated for 1d at rt. Color code for sequence representation: Yellow hydrophobic moiety, blue cationic amino acids, red non-polar amino acids, purple polar amino acids, green anionic amino acids.

11 ATR-IR of PA library

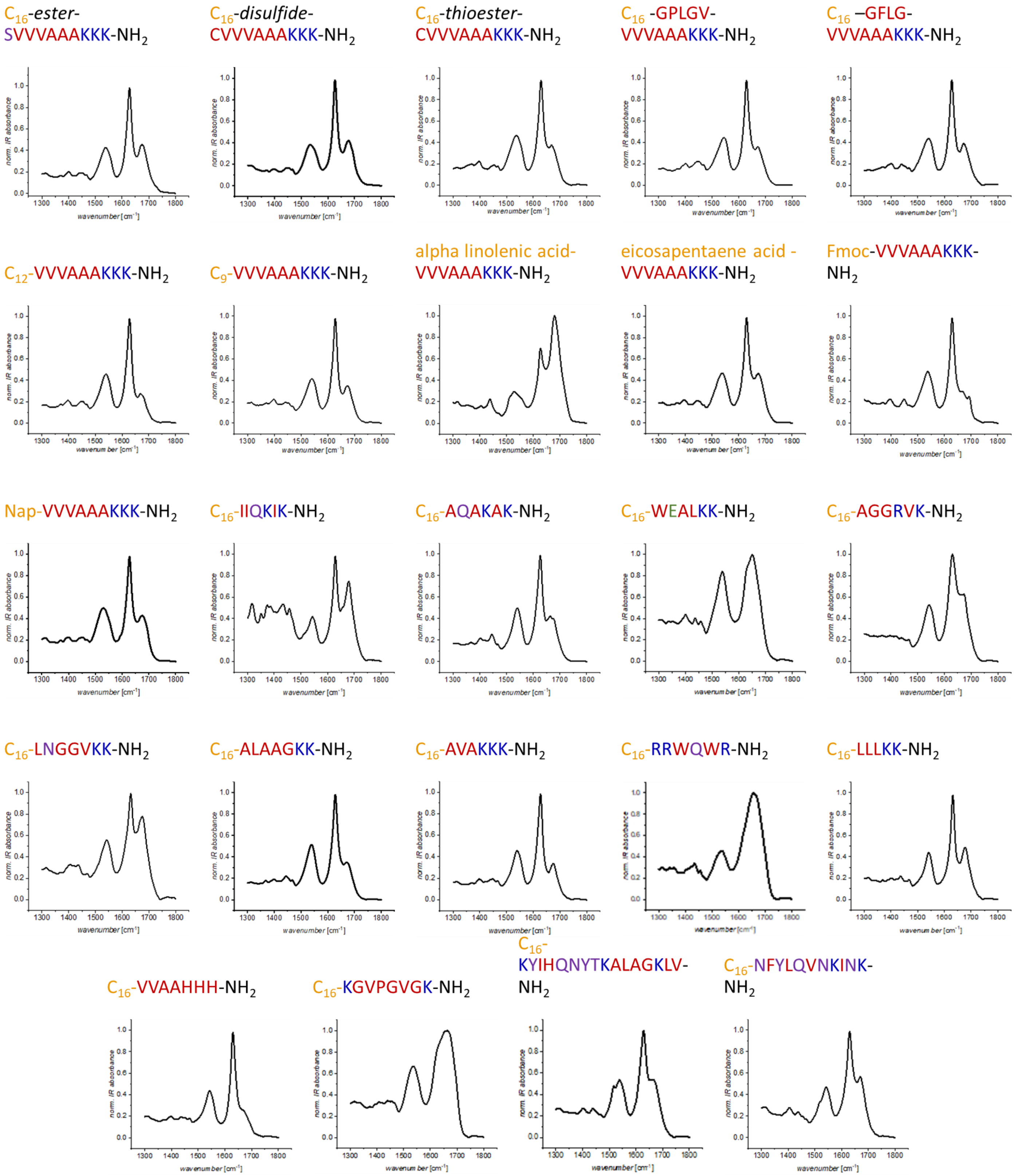

Figure S28 ATR-IR spectra of the PA and peptide library. The compounds were diluted from DMSO (10 mg mL^{-1}) in PBS (1 mg mL^{-1}) and incubated for 1d at rt before lyophilization. Color code for sequence representation: Yellow hydrophobic moiety, blue cationic amino acids, red non-polar amino acids, purple polar amino acids, green anionic amino acids.

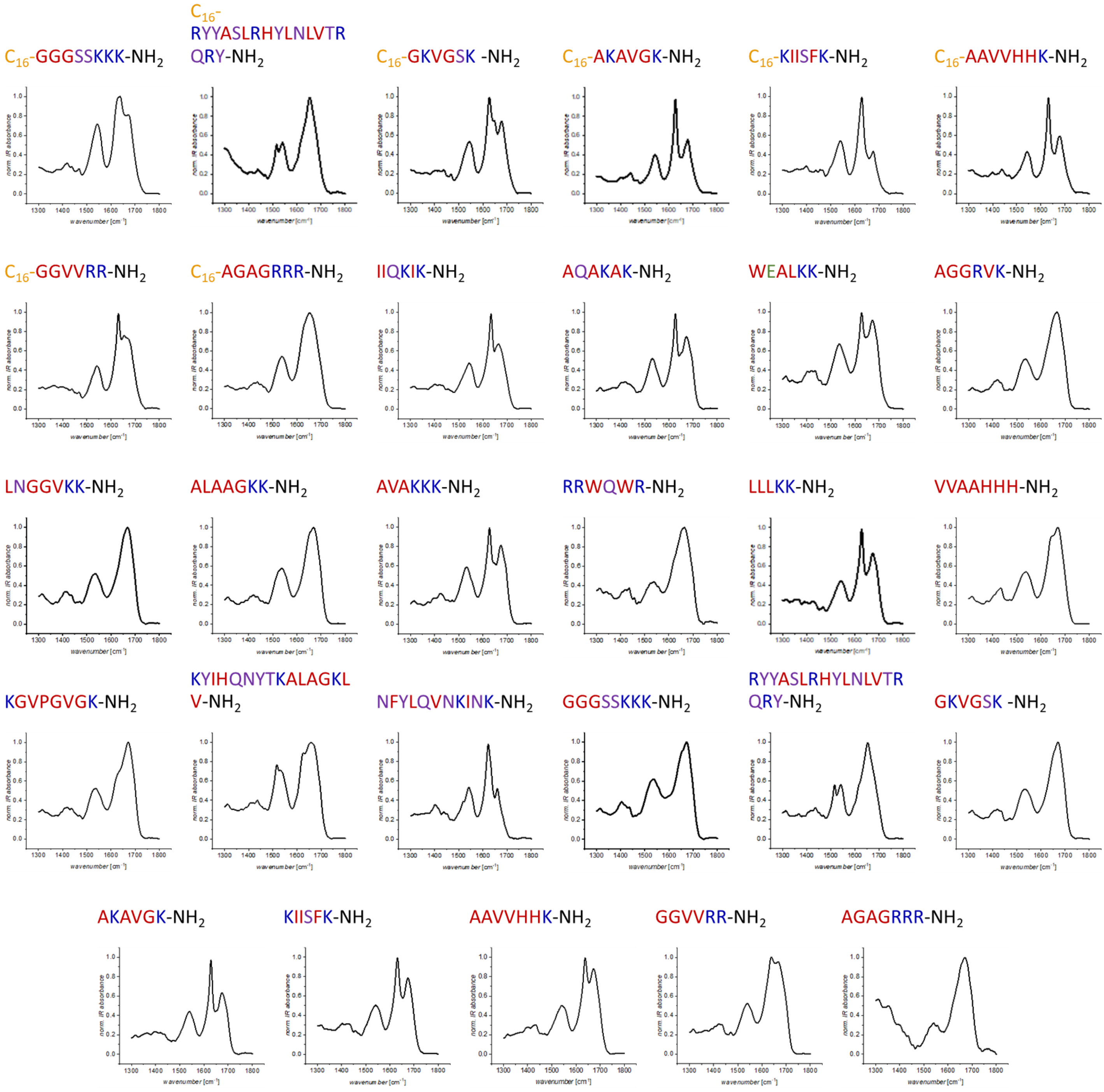

Figure S28 cont. ATR-IR spectra of the PA and peptide library. The compounds were diluted from DMSO (10 mg mL^{-1}) in PBS (1 mg mL^{-1}) and incubated for 1d at rt before lyophilization. Color code for sequence representation: Yellow hydrophobic moiety, blue cationic amino acids, red non-polar amino acids, purple polar amino acids, green anionic amino acids.

12 Chemical structures of PA library

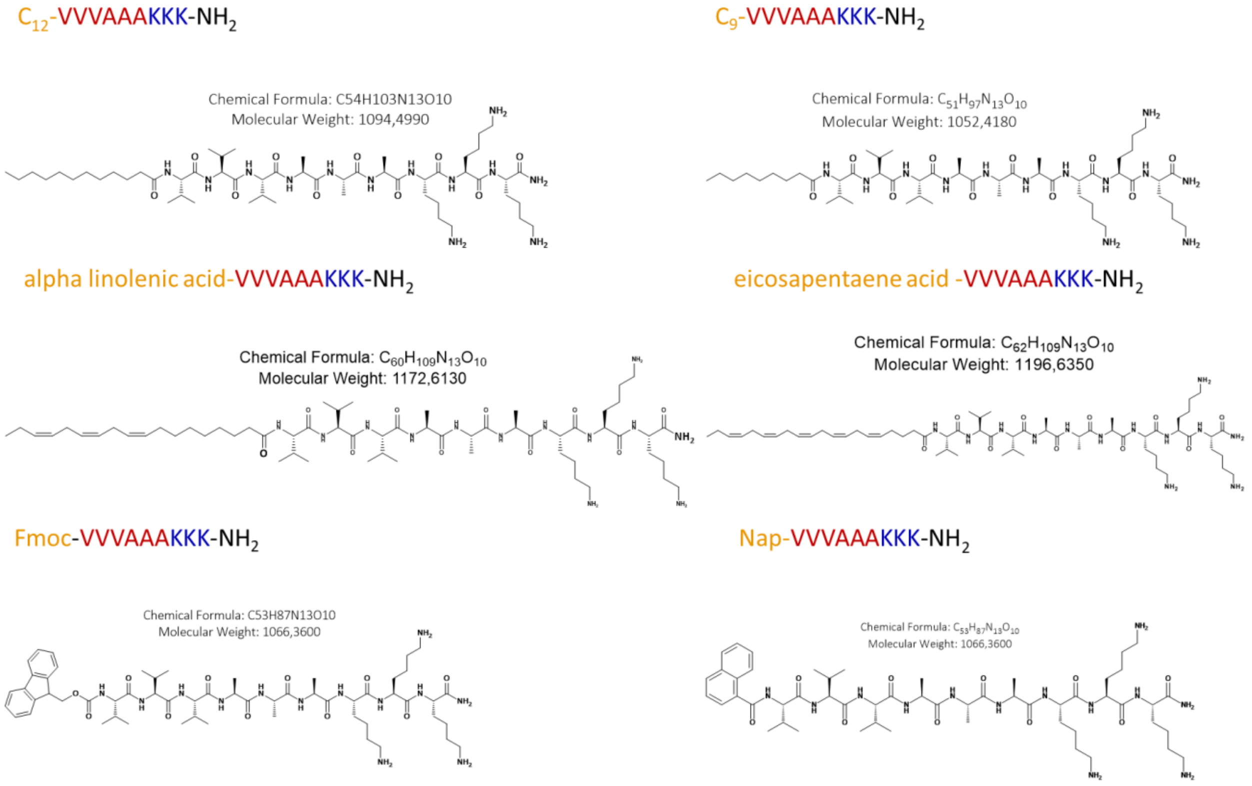

Figure S29 Chemical structure of the PA library with exchange of hydrophobic moiety. The PAs and their respective peptides without hydrophobic moiety were commercially obtained. Color code for sequence representation: Yellow hydrophobic moiety, blue cationic amino acids, red non-polar amino acids, purple polar amino acids, green anionic amino acids.

C_{16}-*ester*-SVVVAAAKKK-NH_2

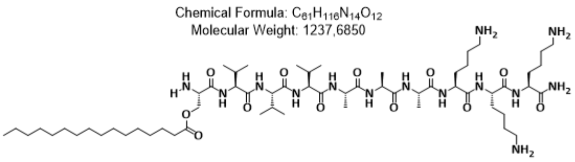

C_{16}-*disulfide*-CVVVAAAKKK-NH_2

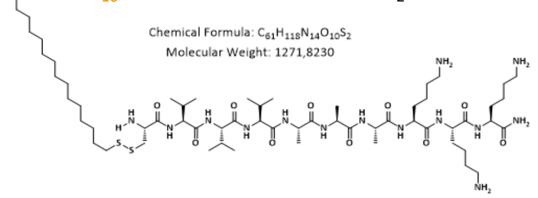

C_{16}-*thioester*-CVVVAAAKKK-NH_2

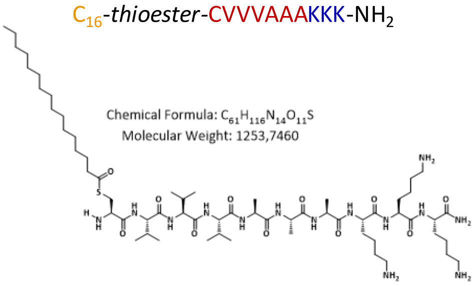

C_{16} -GPLGV-VVVAAAKKK-NH_2

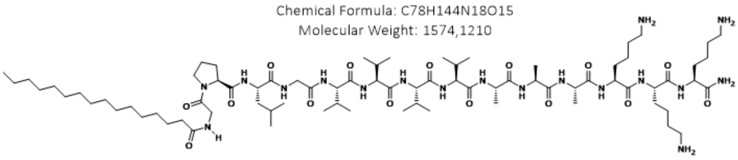

C_{16} -GFLG- VVVAAAKKK-NH_2

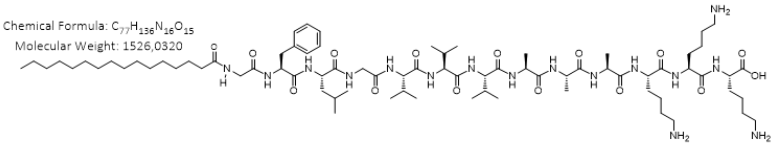

Figure S30 Chemical structure of the PA library with exchange of linker. The PAs and their respective peptides without hydrophobic moiety were commercially obtained. Color code for sequence representation: Yellow hydrophobic moiety, blue cationic amino acids, red non-polar amino acids, purple polar amino acids, green anionic amino acids.

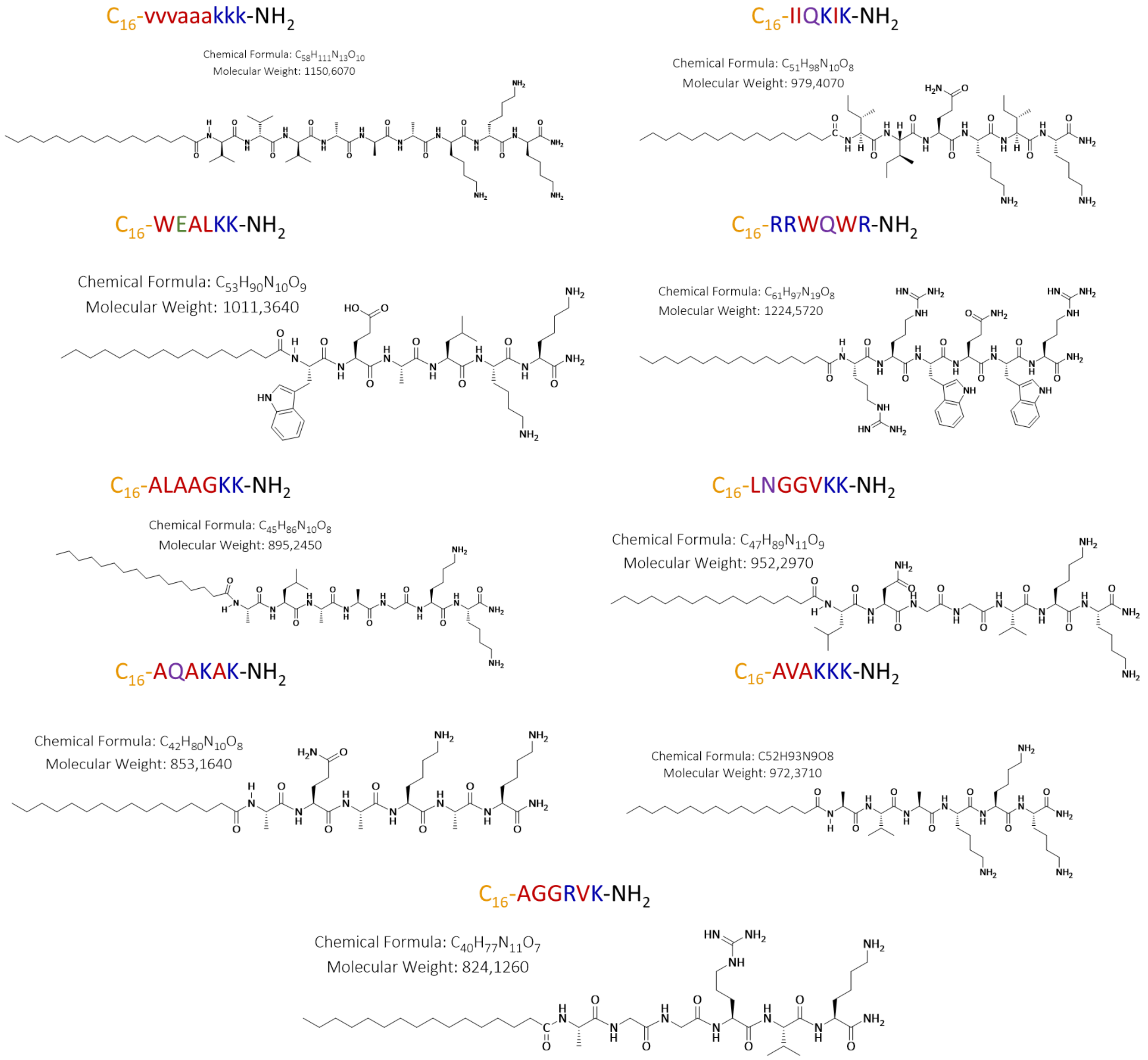

Figure S31 Chemical structure of the PA library with exchange of peptide sequence. The PAs and their respective peptides without hydrophobic moiety were commercially obtained. Color code for sequence representation: Yellow hydrophobic moiety, blue cationic amino acids, red non-polar amino acids, purple polar amino acids, green anionic amino acids.

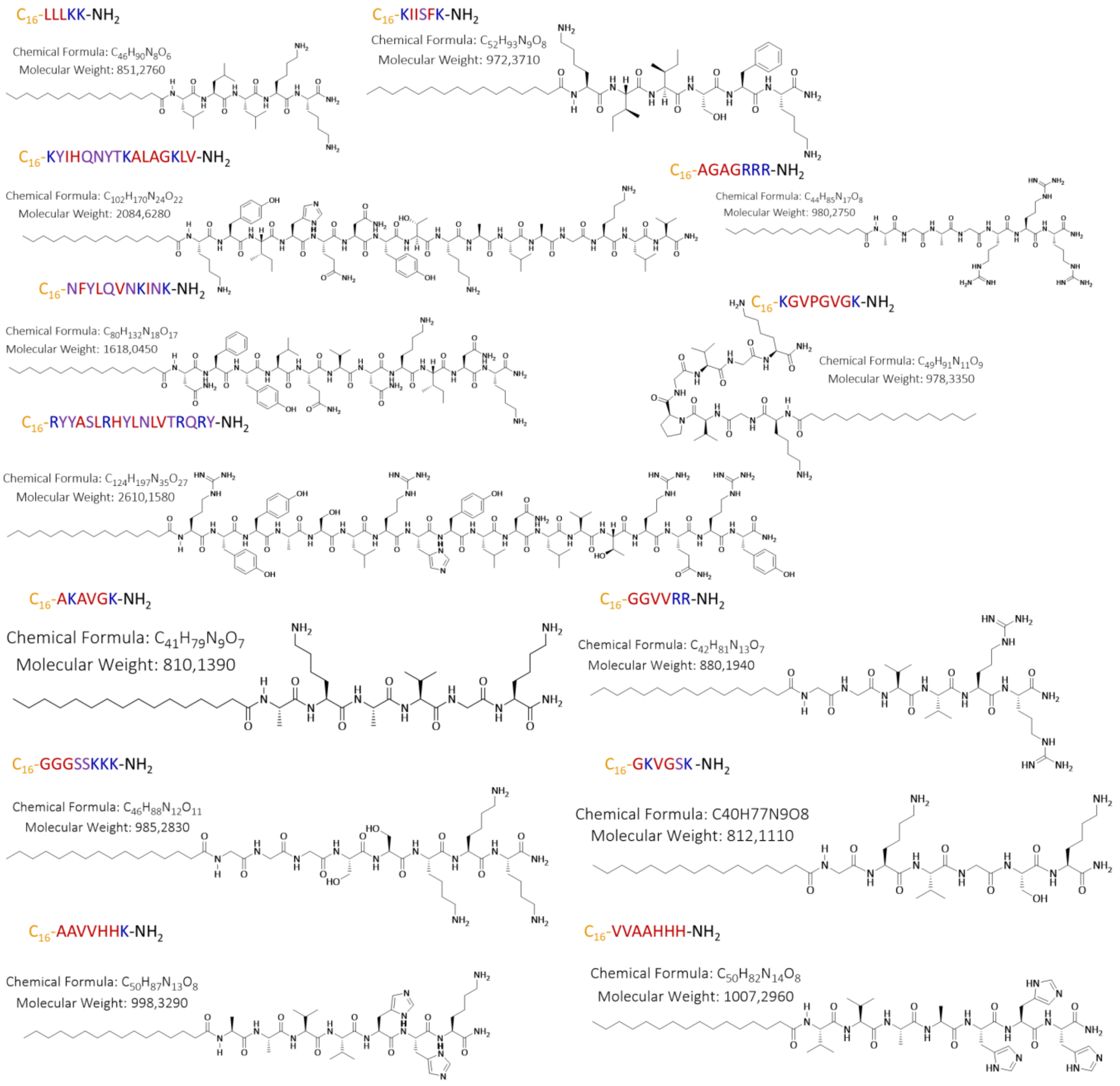

Figure S32 Chemical structure of the PA library with exchange of peptide sequence. The PAs and their respective peptides without hydrophobic moiety were commercially obtained. Color code for sequence representation: Yellow hydrophobic moiety, blue cationic amino acids, red non-polar amino acids, purple polar amino acids, green anionic amino acids.

13 Computational Modeling

13.1 Methods

In order to study the self-assembly of peptides into supramolecular nanofibrils, we performed molecular dynamics (MD) simulations using the MARTINI coarse-grained model version 2.2.[15] We opted to use the MARTINI force field here because we are interested in the microscopic aggregation of the peptides in solution in order to make connections with the even larger scale experiments. However, it is important to mention that the MARTINI force field does not provide information about how the secondary structure of the peptides change over time and we do not use our simulations to determine secondary structures. Therefore, when evaluating fibril-fibril interactions, we assume that the structure of the peptides that form the fibril remain the same. We simulated three different peptide amphiphiles (PAs) that show different aggregation behaviors based on our experimental data: C_{16}-VVVAAAKKK-NH_2 (PA1), C_{16}-FQFKFKC-NH_2 (PA2) and C_{16}-AGGRVK-NH_2. For each of the PAs, the C_{16} tail was represented by 4 beads based on earlier work.[16,17] The rest of the peptide sequence was modeled using standard MARTINI bead types for amino acids for pH of 7.4. The amidated C-terminal of the PAs was modeled using a polar, uncharged standard backbone bead (P5). Using the Packmol[18] software, we prepared the initial configuration of our simulations by randomly distributing 126 PAs in a box with initial size 13 nm × 13 nm × 6.5 nm, which makes a concentration of 0.11 peptides/nm^3 or 0.18 M. This high concentration allows us to accelerate the diffusion of peptides and study the self-assembly process at a reasonable computational cost. Although this concentration is very high compared to the experimental conditions, we argue that there will be similar high concentration regions in experiments before the self-assembly process takes place. Each system was solvated using Polarizable MARTINI water model[19] and neutralized with counterions (Cl^-) in order to account for the total charge on PA molecules. We also added 0.138 M NaCl to mimic experimental conditions. The exact number of water and ions differ slightly for each PA depending on the size and net charge of the PA molecule. For the system with PA1, there are a total of 126 PAs, 6133 water, 476 Cl^- and 98 Na^+ molecules. For PA2, there are 126 PAs, 6288 water, 350 Cl^- and 98 Na^+ molecules. For C_{16}-AGGRVK-NH_2, there are 126 PAs, 6689 water, 350 Cl^- and 98 Na^+ molecules.

We performed all simulations with Gromacs 2019.4[20,21] software according to the following protocol: (1) 10,000 steps minimization using steepest descent method, (2) 1 ns NVT (constant number of molecules, volume and temperature) simulation using 10 fs time step at 298 K, (3) 15 µs NPT (constant number of molecules, pressure and temperature) with 20 fs time step at 298 K and 1 bar pressure. We used the v-rescale coupling method[22] for temperature and Parrinello-Rahman barostat[23,24] for pressure coupling. The compressibility for the pressure coupling was 3×10^{-4} bar^{-1}. Bond constraints were handled using the LINear Constraint Solver (LINCS) algorithm.[25] The Verlet cutoff scheme[26] was used for neighbor searching. Long-range electrostatics were treated with the reaction field method. A cut-off of 1.1 nm was used for evaluation of all non-bonded interactions. For electrostatic interactions, we used the group method with dielectric constant 2.5, which is appropriate for polarizable Martini water model. The neighbor list was updated every 10 steps. Atomic coordinates were saved every 500 ps for the trajectory analysis. Periodic boundary conditions were set in x, y and z directions. All snapshots from simulations were rendered using the Visual Molecular Dynamics (VMD) software.[27]

After 15 µs of self-assembly simulations, we extracted the coordinates of the equilibrated PA fibrils to set up a larger simulation with 4 fibrils to study the aggregation behavior of the fibrils in solution. To

this end, we replicated the equilibrated self-assembled PA for each sequence 4 times and placed them in proximity from each other as shown in **Figure S33**. The initial distance between center of mass of fibrils in the box was about 2 times their diameter. At this distance, the peptides from different fibrils can interact with each other and aggregate or be repelled away based on the nature of their interaction. The initial box size along the length of the fibril (z) was taken directly from the self-assembly simulations (~6.5 nm) and the box size for the other two dimensions (x, y) were set to 18 nm. There was a total of 504 PAs (126 × 4) in each box. The simulation boxes were solvated and ionized as before. The same equilibration protocol was used for these simulations, but the total simulation time was 10 µs. For PA1, there are 13266 water, 1736 Cl^- and 224 Na^+ molecules. For PA2, there are 19366 water, 1301 Cl^- and 293 Na^+ molecules. For C_{16}-AGGRVK-NH_2, there are 11486 water, 1188 Cl^- and 180 Na^+ molecules.

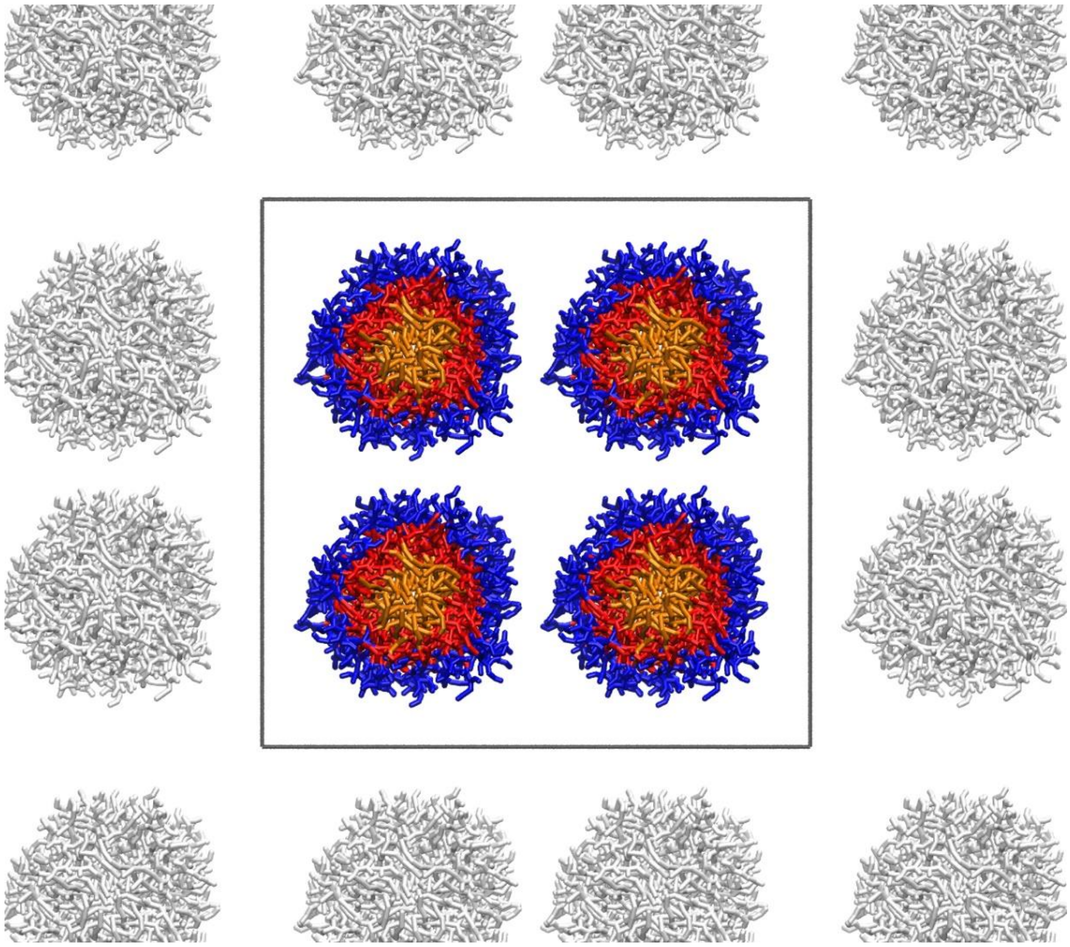

Figure S33 The cross-sectional (top) view of initial configuration of four self-assembled PA fibers for C_{16}-VVVAAAKKK-NH_2 (PA1). The PAs are colored according to the type of residues: C_{16} tail in orange, nonpolar amino acids in red, polar amino acids in purple (none in this PA sequence), and basic amino acids in blue. The periodic images in x and y are shown in white. The simulation box boundaries are drawn in black. Water and ions are omitted for clarity.

To quantitatively represent the aggregation, the distance between the center of mass of fibrils $Dist_{fiber-fiber}$ 1 to 4 over time was measured. Because each PA sequence has a different number of amino acids, the distance was normalized by the diameter of a single fiber D_f to ensure comparability in changes of relative distance. In the initial state (time=0), the $Dist_{fiber-fiber}/D_f$ is > 1.0 because the fibrils are not in contact with each other. If two fibrils are in contact at their surfaces the $Dist_{fiber-fiber}/D_f$ is equal 1.0. If the fibrils aggregate and there is mixing of monomers between the fibrils, the value of $Dist_{fiber-fiber}/D_f$ will be less than 1.

In **Figure S34** the changes of fibril-fibril distance upon multi-fibril simulation are shown and clearly shows the aggregation of PA2 whereas the fibril-fibril distance of PA1 and C_{16}-AGGRVK-NH_2 stay unchanged.

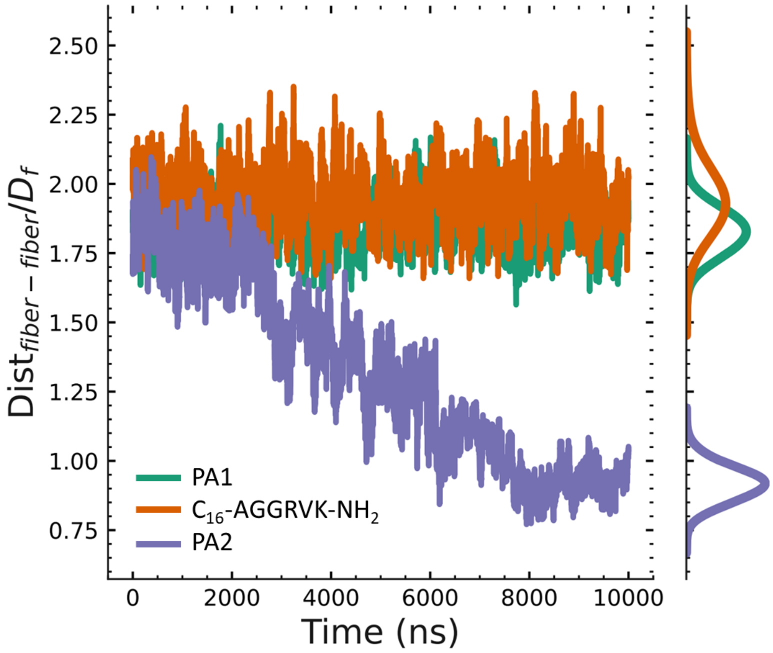

Figure S34 The distance was averaged over 4 fibers, which equals 6 distances.

13.2 Comments on Solvent Accessible Surface Area (SASA)

By calculating the total SASA (normalized by the length of the PA chain) for each simulation, as well as SASA per residue we can learn about the total solvent accessible solvent area of a fibril and how each amino acid in the sequence contributes to this solvent accessibility.

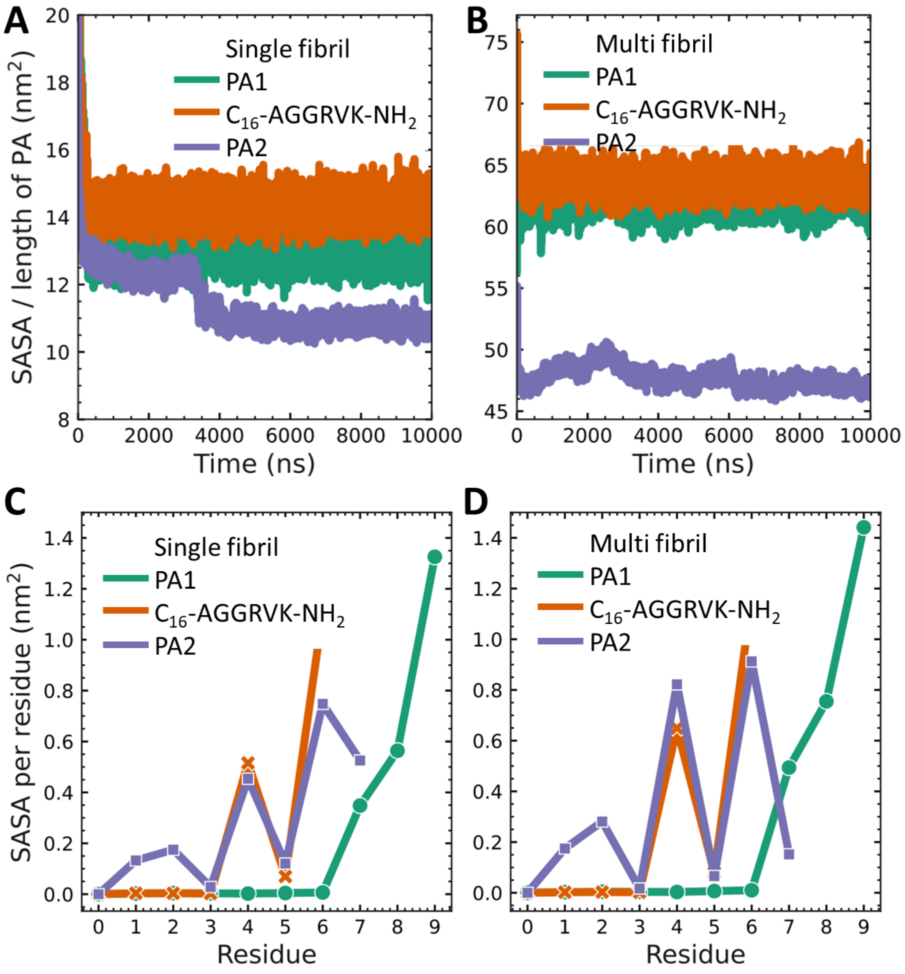

Figure S35 Quantitative evaluation of the solvent accessible surface area (SASA) of single and multiple-fibril simulations. **A** Time-dependent single fibril SASA normalized to the amino acid length of the PA. **B** Time-dependent multi fibril SASA normalized to the amino acid length of the PA**. C** SASA per amino acid residue of the single fibril and **D** of the multi-fibril simulation.

The total SASA shows that both single fibril and multi-fibril simulations have the same trend (**Figure S35A, B**), and the total SASA for PA2 is low compared to the other PAs in multi fibril simulations. This shows the aggregation of the fibrils in PA2.

The SASA per residue shows for example in the case of PA1 that the PA1 is even more hydrophobic than PA2 until the polar, charged lysine residues "KKK "residues that point towards the fibril surface and increase the SASA drastically (**Figure S35C**). For PA2 and C_{16}-AGGRVK-NH_2, the fluctuation in the SASA observed for residues 3-6, is due to the polar and nonpolar amino acids (**Figure S35C**). For both C_{16}-AGGRVK-NH_2 and PA2 (C_{16}-FQFKFKC-NH_2), the residues 4 and 6 are polar and cause an increase of SASA. However, for C_{16}-AGGRVK-NH_2, the polar residue 6 (lysine) is the last residue in the sequence

that results in a polar fibril surface comparable to PA1 (**Figure S35C**). In contrast, for PA2, the SASA decreases for the final nonpolar residue (cysteine), which renders the fibril surface hydrophobic (**Figure S35C**). The contributions of the final amino acids to the increase/decrease of fibril surface hydrophobicity is even more pronounced in multi-fibril simulations (**Figure S35D**).

13.3 Comments on Root Mean Square Fluctuation (RMSF)

The root mean square fluctuation (RMSF) analysis provides quantitative information on how fibrils respond to water vs. presence of other fibrils nearby. This can be evaluated by comparing single and multi-fibril RMSF with each other.

In single fibril simulations, the average RMSF of PA1 and PA2 is similarly low in contrast to the high RMSF of single fibril C_{16}-AGGRVK-NH_2 (**Figure S36A, B**). However, there is a larger deviation for RMSF of PA2 (**Figure S36A**), which means that some monomers are more dynamic (high RMSF) compared to others (low RMSF). The RMSF for PA1 is lower for all residues compared to PA2, which indicates a more stable assembly in solution when there is only a single fibril (**Figure S36A**). However, this observation inverts once multi-fibril interactions are considered (**Figure S36C, D**).

In the multi-fibril scenario, the fibrils already aggregated and reached equilibrium. Now, PA1 and C_{16}-AGGRVK-NH_2 show comparable high RMSF and PA2 a low average RMSF (**Figure S36C, D**) that indicates that the fibrils a PA1 and C_{16}-AGGRVK-NH_2 are less stable and more dynamic than PA2. The high distribution of RMSF for multi-fibril aggregated PA2 indicate that for some parts of the fibril the monomers are more mobile within the aggregated structure than others, but because the aggregated fibril is more stable than not aggregating fibrils the average RMSF for all residues is low.

In other words, fibrils from PA2 aggregate to form a network, in which monomers are still free to move around, but more restricted compared to monomers from PA1 and C_{16}-AGGRVK-NH_2 because PA2 monomers display more of favorable interactions such as low SASA that makes them aggregate. Finite size effects that result from the size of the simulation box play an important role in the network formation. However, we compare different PAs using the same simulation size and only PA2 results in formation of networks, while the other two PA fibrils remain isolated in solution.

For PA1 and C_{16}-AGGRVK-NH_2, the RMSF increases in presence of other fibrils, because there is some interaction between fibrils that is not leading to aggregation, but it is also making them more mobile.

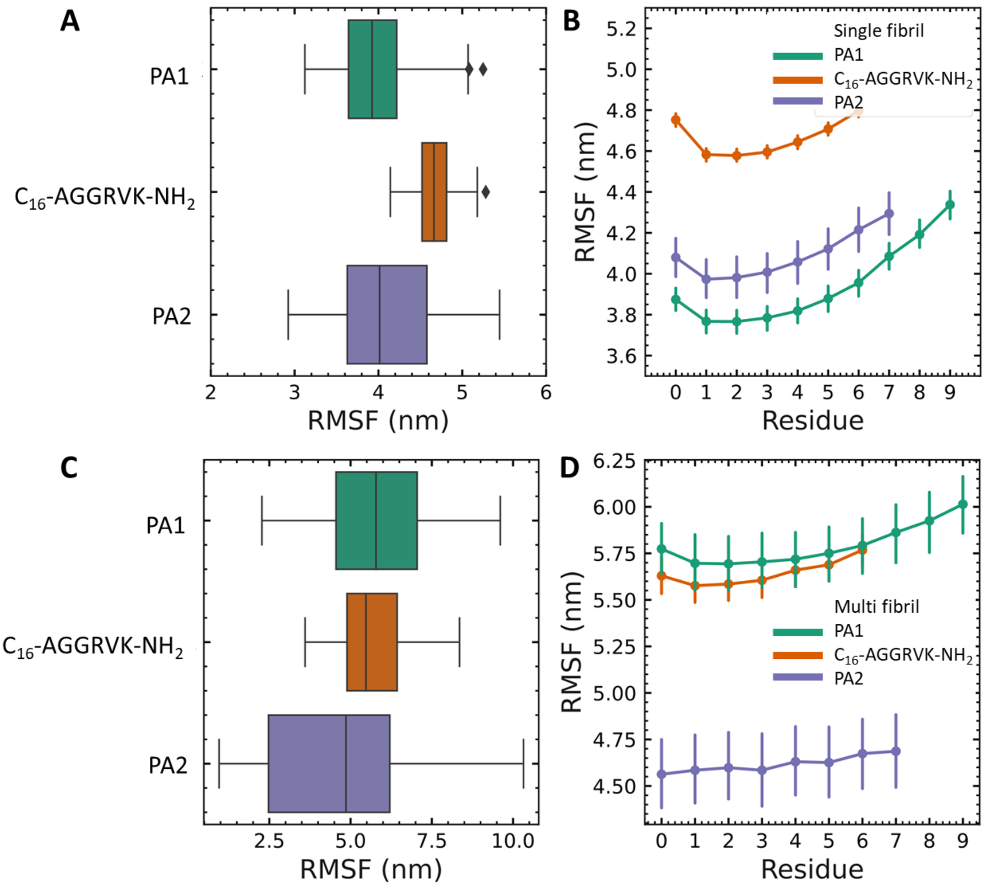

Figure S36 Root mean square fluctuation (RMSF) analysis of single and multi fibril PA simulations. **A** Box plot showing the distribution of RMSF in a single fibril. Solid line represents the median, the boxes show 50% and the whiskers 100% of the data. **B** Mean RMSF per residue in single fibrils, error bars represent standard deviation. **C** Box plot showing the distribution of RMSF in multi fibril simulations. Solid line represents the median, the boxes show 50% and the whiskers 100% of the data. **D** Mean RMSF per residue in multi fibril simulations, error bars represent standard deviation.

14 References

[1] E. Goormaghtigh, V. Cabiaux, J. Ruysschaert, *Eur. J. Biochem.* **1990**, *193*, 409.

[2] D. M. Byler, H. Susi, *Biopolymers* **1986**, *25*, 469.

[3] M. Jackson, H. H. Mantsch, *Crit. Rev. Biochem. Mol. Biol.* **1995**, *30*, 95.

[4] A. Adochitei, G. Drochioiu, *Rev. Roum. Chim.* **2011**, *56*, 783.

[5] V. A. Wagoner, M. Cheon, I. Chang, C. K. Hall, *Proteins Struct. Funct. Bioinforma.* **2014**, *82*, 1469.

[6] R. Sarroukh, E. Cerf, S. Derclaye, Y. F. Dufrêne, E. Goormaghtigh, J. M. Ruysschaert, V. Raussens, *Cell. Mol. Life Sci.* **2011**, *68*, 1429.

[7] E. Cerf, R. Sarroukh, S. Tamamizu-Kato, L. Breydo, S. Derclayes, Y. F. Dufrênes, V. Narayanaswami, E. Goormaghtigh, J. M. Ruysschaert, V. Raussens, *Biochem. J.* **2009**, *421*, 415.

[8] R. Sarroukh, E. Goormaghtigh, J.-M. Ruysschaert, V. Raussens, *Biochim. Biophys. Acta - Biomembr.* **2013**, *1828*, 2328.

[9] A. Gustot, V. Raussens, M. Dehousse, M. Dumoulin, C. E. Bryant, J. M. Ruysschaert, C. Lonez, *Cell. Mol. Life Sci.* **2013**, *70*, 2999.

[10] M. Adachi, M. So, K. Sakurai, J. Kardos, Y. Goto, *J. Biol. Chem.* **2015**, *290*, 18134.

[11] S. Sieste, T. Mack, E. Lump, M. Hayn, D. Schütz, A. Röcker, C. Meier, K. Kaygisiz, F. Kirchhoff, T. P. J. Knowles, F. S. Ruggeri, C. V. Synatschke, J. Münch, T. Weil, *Adv. Funct. Mater.* **2021**, *31*, 2009382.

[12] C. Schilling, T. Mack, S. Lickfett, S. Sieste, F. S. Ruggeri, T. Sneideris, A. Dutta, T. Bereau, R. Naraghi, D. Sinske, T. P. J. Knowles, C. V. Synatschke, T. Weil, B. Knöll, *Adv. Funct. Mater.* **2019**, *29*, 1809112.

[13] K. Ryu, G. J. Lee, J.-Y. Choi, T. Kim, T.-I. Kim, *Adv. Mater. Sci. Eng.* **2015**, *2015*, 1.

[14] R. Shankar, A. Samykutty, C. Riggin, S. Kannan, U. Wenzel, R. Kolhatkar, *Mol. Pharm.* **2013**, *10*, 3776.

[15] D. H. de Jong, G. Singh, W. F. D. Bennett, C. Arnarez, T. A. Wassenaar, L. V. Schäfer, X. Periole, D. P. Tieleman, S. J. Marrink, *J. Chem. Theory Comput.* **2013**, *9*, 687.

[16] O.-S. Lee, V. Cho, G. C. Schatz, *Nano Lett.* **2012**, *12*, 4907.

[17] C. Li, A. Iscen, H. Sai, K. Sato, N. A. Sather, S. M. Chin, Z. Álvarez, L. C. Palmer, G. C. Schatz, S. I. Stupp, *Nat. Mater.* **2020**, *19*, 900.

[18] L. Martínez, R. Andrade, E. G. Birgin, J. M. Martínez, *J. Comput. Chem.* **2009**, *30*, 2157.

[19] S. O. Yesylevskyy, L. V. Schäfer, D. Sengupta, S. J. Marrink, *PLoS Comput. Biol.* **2010**, *6*, e1000810.

[20] H. J. C. Berendsen, D. van der Spoel, R. van Drunen, *Comput. Phys. Commun.* **1995**, *91*, 43.

[21] M. J. Abraham, T. Murtola, R. Schulz, S. Páll, J. C. Smith, B. Hess, E. Lindahl, *SoftwareX* **2015**, *1–2*, 19.

[22] G. Bussi, D. Donadio, M. Parrinello, *J. Chem. Phys.* **2007**, *126*.

[23] M. Parrinello, A. Rahman, *J. Appl. Phys.* **1981**, *52*, 7182.

[24] S. Nosé, M. L. Klein, *Mol. Phys.* **1983**, *50*, 1055.

[25] B. Hess, H. Bekker, H. J. C. Berendsen, J. G. E. M. Fraaije, *J. Comput. Chem.* **1997**, *18*, 1463.

[26] H. Grubmüller, H. Heller, A. Windemuth, K. Schulten, *Mol. Simul.* **1991**, *6*, 121.

[27] W. Humphrey, A. Dalke, K. Schulten, *J. Mol. Graph.* **1996**, *14*, 33.
